# Towards a comprehensive taxonomic revision of the Neotropical dung beetle subgenus *Deltochilum* (*Deltohyboma*) Lane, 1946 (Coleoptera: Scarabaeidae: Scarabaeinae): Division into species-groups

**DOI:** 10.1371/journal.pone.0244657

**Published:** 2021-01-06

**Authors:** Arturo González-Alvarado, Fernando Z. Vaz-de-Mello

**Affiliations:** 1 Programa de Pós-graduação em Ecologia e Conservação da Biodiversidade, Universidade Federal de Mato Grosso, Cuiabá, Mato Grosso, Brazil; 2 Departamento de Biologia e Zoologia, Instituto de Biociências, Universidade Federal de Mato Grosso, Cuiabá, Mato Grosso, Brazil; Nanjing Agricultural University, CHINA

## Abstract

*Deltochilum* Eschscholtz, 1822 is perhaps the most speciose genus of the tribe Deltochilini *sensu* Tarasov & Dimitrov (2016) (Scarabaeidae: Scarabaeinae) and has been traditionally divided into eight subgenera. Among them, the subgenus *Deltohyboma* Lane, 1946, is the most speciose with 47 species, five of which are described here (*D*. *genieri* **sp. nov.**, *D*. *gilli* **sp. nov.**, *D*. *susanae* **sp. nov.**, *D*. *bolivariensis* **sp. nov.** and *D*. *inesae* **sp. nov.**), and at least 165 species still undescribed. Due to the large number of species, and for practical purposes, the subgenus is here divided into 19 species-groups, with *D*. *inesae* **sp. nov.** left as *incertae sedis*. This division into species-groups will help in the reliable identification of species and will aid in the completion of the revision of subgenus. This report is the first part of the taxonomic revision of the subgenus *Deltohyboma*; it is based on the examination of all type specimens and almost 9,800 specimens of which approximately 1,200, mostly males, had their genitalia studied. The 19 species-groups recognized here are based mainly on characters described for the first time for *Deltohyboma*, namely, the state of a) the anterior margin of the clypeus (between the clypeal teeth), b) the internal margin of hypomera, c) the ventral face of the protibia, d) the posterior margin of the metafemur, and e) several new characters resulting from the first detailed study of the aedeagus and the endophallus of the group. Diagnosis, description, geographic distribution, composition and identifications keys (for males as well as males and females) for the species-groups are provided.

## Introduction

Dung beetles (Scarabaeinae) are considered a focal taxon for biodiversity studies, monitoring, conservation and as ecological indicators [1–3] and probably for these reasons, Scarabaeinae is the second most cited subfamily of beetles on Google Scholar [4]. The Neotropical genus *Deltochilum* Eschscholtz, 1822 is a common part of the dung beetle community, mainly in South America. Some studies have found *Deltochilum* to be one of the most abundant genera of the Coleoptera community [5], with some species being some of the most abundant in the Scarabaeinae community [6, 7]. Usually, the number of species of *Deltochilum* at a given locality varies between three [6] to seven [7], but it can reach 10 (pers. obs.). However, identification of the species belonging to the subgenus *Deltohyboma* Lane, 1946, by far the largest subgenus, is practically impossible without reference to type specimens. No comprehensive keys for species identification of *Deltohyboma* are published, and the last taxonomic revision [8, 9] is outdated, with several species being very difficult to separate; characters of the endophallus are often needed to distinguish closely related species. Perhaps this is the reason why most species are identified as “sp.” or “*aff*” in many ecological studies (e.g. [7, 10–13]).

*Deltochilum* is probably the most speciose genus of the tribe Deltochilini (*sensu* [14]) currently including 110 valid species, five new species described here, and at least 165 new species remain to be described. *Deltochilum* is also one of the most heterogeneous groups, evidenced by: a) the early subdivisions of genus made by Burmeister [15] into three groups (I, II, III) based on elytral carinae (characters still used for the classification of the subgenera) of the fewer than 20 species know at the time; b) the seven subgenera formally proposed by Kolbe in [16], for the almost 50 known species at the time, three of these subgenera remain valid (*D*. (*Aganhyboma*) Kolbe, 1893, *D*. (*Calhyboma*) Kolbe, 1893 and *D*. (*Euhyboma*) Kolbe, 1893).

Between the work of Burmeister [15] and the subgeneric classification of Kolbe [16] several authors described new species, commonly one or two species in each contribution. The most prolific authors were Bates, who between 1870 and 1887, described 14 new species [17, 18], followed by Harold [19–23], who added seven new species.

Some years after Kolbe’s [16] paper, in which the type species of his subgenera were not designated, Shipp [24] designated the type species for seven subgenera that he considered valid (treated as genera), corrected Kolbe’s [16] nomenclatural inaccuracies and introduced a new generic name (*Hybomidium*) (see González & Vaz-de-Mello [25] and Silva *et al*. [26] for more details).

In the following 40 years only five new species were described [27–29], before Paulian’s taxonomic revision of *Deltochilum*, published in two consecutive years (1938–1939) [8, 9] where redescriptions of species, descriptions of 15 new species, new subgenera and synonymies were presented. At the same time Balthasar [30] described 13 new species, three of which were synonymised by Génier [31] with species previously described by Paulian [8].

In the revision of Paulian [8], new subgenera were proposed without taking into account the nomenclatural emendations and the designation of type species made by Shipp [24]. Finally Lane [32] amended the nomenclatural inaccuracies made by Paulian [8], synonymised and designated types species for the valid subgenera and erected a new subgenus *Deltohyboma* (see González & Vaz-de-Mello [25] and Silva *et al*. [26] for more details for the other subgenera).

Since Lane [32] nine subgenera were recognized, until Génier [33] who synonymised *D*. (*Telhyboma*) Kolbe, 1893 with *Deltochilum s*. *str*. (see Table 1 for a summary of nomenclatural acts and descriptions of the species groups); currently leaving eight valid subgenera (valid species in parentheses): *Aganhyboma* (27), *Calhyboma* (12), *Deltochilum s*.*tr*. (7), *Deltohyboma* Lane, 1946 (47), *Euhyboma* (1), *Hybomidium* Shipp, 1897 (13), *Rubrohyboma* Paulian, 1938 (1) and *Parahyboma* Paulian, 1938 (2).

**Table 1:** Nomenclatural acts and subgenus- or genus-groups names proposed for *Deltochilum*.

Authorship and year	Genus or Subgenus- names	Subgenus or genus- group category	Type species	Type fixation	Current status	Invalid–authorship invalidation
Eschscholtz, 1822	*Deltochilum*	Genus	*D*. *dentipes* Eschscholtz, 1822	Monotypy	Valid	N/A
Vigors, 1826	*Anamnesis*	Genus	*A*. *macleayii* Vigors, 1826	Monotypy	Invalid	Objective synonym of *Deltochilum*—Shipp [24]⁠
Le Peletier de Saint-Fargeau & Audinet-Serville, 1828	*Hyboma*	Genus	*Ateuchus gibbosa* Fabricius, 1775	Original designation	Invalid	Junior synonym of *Deltochilum* and name preocupied by Hübner, 1816—Shipp [24]⁠
Kolbe, 1893	*Paedhyboma*	Subgenus	*Deltochilum aberrans* Harold, 1868	Monotypy	Invalid	Junior synonym of *Canthon*—Pereira & d’Andretta [34]⁠
Kolbe, 1893	*Calhyboma*	Subgenus	*D*. *burmeisteri* (Harold, 1867) = *D*. *mexicanum* Burmeister, 1848	Subsequent designation by Shipp (1897)	Valid	N/A
Kolbe, 1893	*Euhyboma*	Subgenus	*D*. *brasiliense* (Castelnau, 1840)	Monotypy	Valid	N/A
Kolbe, 1893	*Aganhyboma*	Subgenus	*D*. *trisignatum* Harold, 1881	Subsequent designation by Shipp (1897)	Valid	N/A
Kolbe, 1893	*Meghyboma*	Subgenus	*D*. *dentipes* Eschscholtz, 1822	Subsequent designation by Shipp (1897)	Invalid	Junior synonym of *Deltochilum*—Shipp [24]⁠
Kolbe, 1893	*Telhyboma*	Subgenus	*D*. *orbiculare* Lansberge, 1874	Monotypy	Invalid	Junior synonym of *Deltochilum*–Génier [33]⁠
Shipp, 1897	*Hybomidium*	Genus	*D*. *icarus* (Olivier, 1789), but *D*. *gibbosum* (Fabricius, 1775)is the valid type species	*D*. *icarus* by original designation, invalided by Lane (1946) and designated *D*. *gibbosum* as valid	Valid	N/A
Paulian, 1938	*Tetraodontides*	Subgenus	*D*. *gibbosum* (Fabricius, 1775)	Original designation	Invalid	Junior synonym of *Hybomidium*—Lane [32]⁠
Paulian, 1938	*Eudantylides*	Subgenus	*D*. *carinatum* (Westwood, 1837)	Original designation	Invalid	Junior synonym of *Calhyboma*- Lane [32]⁠
Paulian, 1938	*Rubrohyboma*	Subgenus	*D*. *rubripenne* (Gory, 1831)	Original designation	Valid	N/A
Paulian, 1938	*Parahyboma*	Subgenus	*D*. *furcatum* (Castelnau, 1840)	Original designation	Valid	N/A
Lane, 1946	*Deltohyboma*	Subgenus	*D*. *submetallicum* (Castelnau, 1840)	Original designation	Valid	N/A

In chronological order. N/A = non applicable.

The subgenus *Deltohyboma* was the last to be described, the species currently belonging to it were included (until 1946) (in part) within *Deltochilum s*. *str*. by Kolbe [16], Paulian [8], Balthasar [30] and *D*. (*Hybomidium*) by Shipp [24]. However, the composition of *D*. (*Deltohyboma*) *sensu* Paulian [8] remained unchanged until recently, when Silva *et al*. [26] transferred some species from *D*. (*Deltohyboma*) to *D*. (*Aganhyboma*).

Not long after of the works of Paulian [8, 9] and Balthasar [30], some American scarabaeidologists recognised the need for a taxonomic revision of *Deltochilum* due to its heterogeneity, the amount of divisions (subgenera) and the numerous new species remaining to be described in collections [34–38]. The attempt towards a modern and comprehensive taxonomic revision of *Deltochilum* focused on subgenera resulted in the description of 24 new species [25, 26, 33, 39, 40] and the description of new genus [41].

Here, the taxonomic revision of the most speciose subgenus of *Deltochilum*, *Deltohyboma*, is initiated. Species-groups are proposed, which will make species identifications more manageable and accessible and will aid in the completion of the revision of the subgenus.

## Material and methods

This work is based on the study of almost 9,800 specimens, 1,200 of which had their genitalia extracted (mostly males). The specimens studied here included primary types of all species of *Deltohyboma* (currently valid or otherwise). Specimens examined are from the following collections (curator(s) in parentheses).

**BDGC**: Bruce D. Gill personal collection, Ottawa, Canada (Bruce Gill)

**BMNH**: Natural History Museum, London, United Kingdom (Maxwell Barclay and Malcolm Kerley)

**CMNC**: Canadian Museum of Nature, Ottawa, Canada (François Génier)

**CNCI**: Canadian National Collection of Insects and Arachnids, Agriculture and Agri-Food Canada, Ottawa, Canada (Pat Bouchard and Serge Laplante)

**CECC**: Colección de Escarabajos coprófagos de Colombia, Bogotá, Colombia

(Alejandro Lopera)

**CEMT**: Seção de Entomologia da Coleção Zoológica, Instituto de Biociências,

Universidade Federal de Mato Grosso, Cuiabá, Brazil (Fernando Vaz-de-Mello)

**IAvH**: Colección Entomológica del Instituto Alexander von Humboldt, Villa de Leyva,

Colombia (Jhon Cesar Neita)

**MACN**: Museo Argentino de Ciencias Naturales ‘Bernardino Rivadavia’, Buenos Aires, Argentina (Juan José Martínez)

**MNHN**: Muséum national d’Histoire naturelle, Paris, France (Antoine Mantilleri and

Olivier Montreuil)

**MLUH**: Martin-Luther-Universität, Zentralmagazin Naturwissenschaftlicher Sammlungen, Zoologische Sammlung, Halle, Germany (Karla Schneider)

**MZUSP**: Museu de Zoologia, Universidade de São Paulo, Brazil (Carlos Campaner and

Sonia Casari)

**NMPC**: National Museum (Natural History), Prague, Czech Republic (Jiří Hájek)

**OUMNH**: Hope Entomological Collections, Oxford University Museum of Natural History, Oxford, United Kingdom (Darren Mann)

**RBINS**: Royal Belgian Institute of Natural Sciences, Brussels, Belgium (Alain Drumont)

**RMNH**: Naturalis Biodiversity Centre, Leiden, the Netherlands (Hans Huijbregts)

**SMF**: Forschungsinstitut und Naturmuseum Senckenberg, Frankfurt-am-Main, Germany

(Andrea Hastenpflug-Vesmanis)

**SMTD**: Senckenberg Naturhistorische Sammlungen Dresden, Museum für Tierkunde,

Dresden, Germany (K.D. Klass; O. Jäger)

**ZMHB**: Museum für Naturkunde der Humboldt Universität, Berlin, Germany (Johannes

Frisch and Joachim Willers)

**ZSM**: Zoologische Staatsammlung, Münich, Germany (Michael Balke and Lars Hendrich)

**Fig 1:**
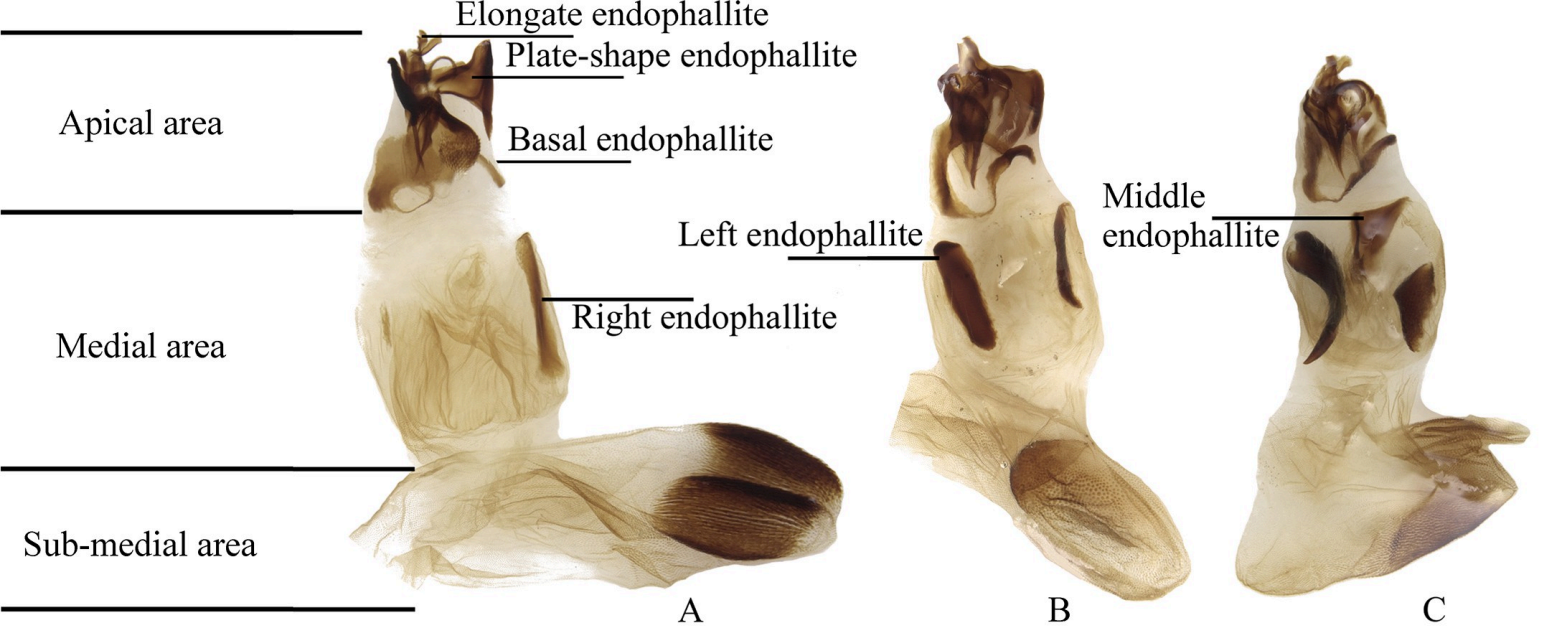
Names used for the endophallus and endophallites. (A) *lindemannae* species-group. (B) *aequinoctiale* species-group. (C) *parile* species-group.

The male genitalia of the greatest possible number of specimens were dissected, including the endophallus. The aedeagus and the endophallus were macerated in a solution of 10% KOH for several minutes depending on the size of the specimen and the condition of the genitalia, following the methodology of Zunino [42] and Medina *et al*. [43]. Names of the structures of the endophallus are based on Medina *et al*. [44] (however, see further discussions and criticisms by Zunino [45]) and Génier [46]. For practical purposes, when the medial area of the endophallus bears two endophallites (Fig 1B), these are named “right” and “left” endophallites according to their position when the endophallus is unnaturally everted, with the plate-shaped endophallite upwards (dorsal) and the basal endophallite ventral. When the median area of the endophallus bears one endophallite (Fig 1A), in the same position, that endophallite is considered to be the “right” one. Rarely, when it bears three endophallites (Fig 1C), these are named “right”, “left” and “middle” endophallite (see Fig 1).

The terms for the external morphology follow Edmonds [47], González & Vaz-de-Mello [25] and Lawrence *et al*. [48]. We follow the terminology of the latter work in using metaventrite and metaventral process instead of the traditional term, metasternum, and for the visible abdominal sternites, we use ventrites. We follow [49] in using the term edge to designate the outermost portion of a structure and the term margin to designate the delimited portion of the edge.

### Distribution data and maps

The distribution of the species-groups is given according to the biogeographical dominions and provinces defined by Morrone [50], helped by the shapefile provided by Löwenberg-Neto [51] on QGIS 3.12 software (under GNU General Public License). The same software was used to construct the distribution maps for the species, which were based on the data provided on the specimen labels and the free vector and raster map data provided, under public domain, by Natural Earth (naturalearthdata.com). Most of the distribution data found in published literature was deemed unusable because the species of *Deltohyboma* were identified as “sp.” or misidentified and because in each locality there are several sympatric species. When such information is used, it is clearly stated in the “Distribution” section of each species.

### Images

Different equipment was used to produce photographic images. For the new species, when possible, the images are of the holotype. This is specified in the plate legend. If the image is of a paratype, this information is also specified in the plate legend, including the locality of that specimen.

The images and the plates were edited and prepared with software under GNU General Public License, GIMP 2.10 and Inkscape 0.92 running on GNU/Linux.

### “Material examined” section

The “material examined” section was prepared using AUTOMATEX [52]. Geographic coordinates, which were used for the construction of the distribution maps, are given between square brackets “[]” for labels without that data. Labels of the type specimens of the species described here are not cited verbatim, but the collection catalogue number for the holotype is provided to enable easy retrieval.

### Nomenclatural acts

The electronic edition of this article conforms to the requirements of the amended International Code of Zoological Nomenclature, and hence the new names contained herein are available under that Code from the electronic edition of this article. This published work and the nomenclatural acts it contains have been registered in ZooBank, the online registration system for the ICZN. The ZooBank LSIDs (Life Science Identifiers) can be resolved and the associated information viewed through any standard web browser by appending the LSID to the prefix “http://zoobank.org/”. The LSID for this publication is: urn:lsid:zoobank.org:pub:70584C6B-3D16-4DC7-9008-497ACEF31896. The electronic edition of this work was published in a journal with an ISSN, and has been archived and is available from the following digital repositories: PubMed Central and LOCKSS.

### Species-groups

Two identification keys are presented for the species-groups proposed here (see “remarks” section of the description of *Deltohyboma*). One for males and females and the other one for males only. The latter is recommended for reliable identification because several species-groups are based, mainly on male secondary sexual characters and, in some cases, the aedeagus provides the best way to verify the identification of the species-group.

The species-groups are organized alphabetically. The names of the species-groups were based on the oldest described species within each group or a new species described here.

A diagnosis, description, composition and distribution are provided for all species-groups. Additionally, in the “distribution” section, apart from the biogeographical dominions and provinces where these are distributed, any sympatric species-group(s), and in which province(s), are also provided.

## Results

### *Deltochilum* (*Deltohyboma*) Lane, 1946

*Deltochilum* (*Deltohyboma*) Lane, [32]: 175; Pereira & Martínez [35]: 121, 122, 191; Martínez [53]: 53; Vulcano & Pereira [54]: 571, 652, 680; Halffter & Matthews [55]: 261; Vulcano & Pereira [56]: 555, 556; Martínez [57]:55; Vaz-de-Mello [58]: 192; González *et al*. [39]: 253, 254; Vaz-de-Mello *et al*. [59]: 5, 26, 33, 41, 44; Krajcik [60]: 88; Boilly & Vaz-de-Mello [61]: 107; González & Vaz-de-Mello [25]: 431; Silva & Vaz-de-Mello [62]: 276; Silva *et al*. [26]: 453; Silva *et al*. [40]: 232; Chamorro *et al*. [63]: 76; Chamorro *et al*. [64]: 95; González *et al*. [41]: 1751.

*Deltochilum* (*Deltochilum s*. *str*.) [cited as *Deltochilum* i. sp.] [in part] Kolbe [16]: 391; Paulian [8]: 240, 242, 243, 268.

*Hybomidium* [in part] Shipp [24]: 195.

#### Type species

*Hyboma submetallicum* Castelnau, 1840 = *Deltochilum submetallicum* (Castelnau, 1840) by subsequent designation by Lane [32], cited as “Ortótipo”.

#### Composition

47 valid species and at least 165 new species to be described.

#### Diagnosis

**Fig 2:**
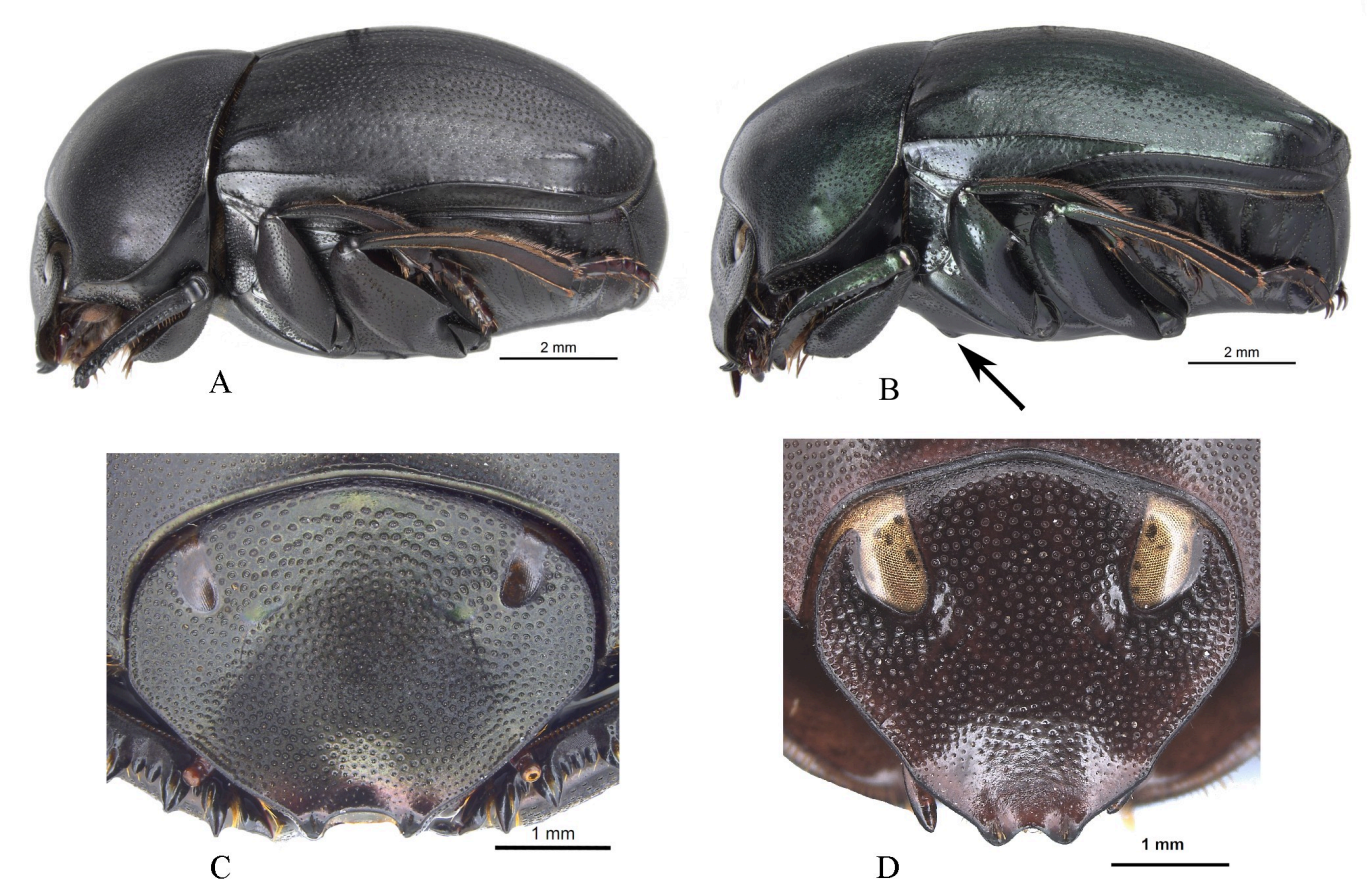
Body lateral view (A-B) and head (C-D). (A) *guyanense* species-group. (B) *aequinoctiale* species-group, arrow showing tubercle on metaventrite. (C) *submetallicum* species-group. (D) *valgum* complex, subgenus *Aganhyboma*.

Within *Deltochilum* the subgenus *Deltohyboma* can be distinguished by the following combinations of characters: head approximately 1.5x as wide as long (Fig 2C); anterior margin of the clypeus with two teeth; carina of the interstria IX variable in length, from almost reaching the basal third of the elytral (Fig 2A) to slightly surpassing the middle of the elytral length (Fig 2B).

It is possible to identify species belonging to *Deltohyboma* using the key for American genera of Scarabaeinae [59]. However, after Silva *et al*. [26] transferred some species of *Deltohyboma* to *D*. (*Aganhyboma*), the species now belonging to *acropyge* and *valgum* complexes of the subgenus *Aganhyboma* (see Silva *et al*., [26]) would still be identified as if they were *Deltohyboma*. However, these species can be correctly identified as belonging to *D*. (*Aganhyboma*) by the head, which is slightly wider than long (Fig 2D).

#### Redescription

**Fig 3:**
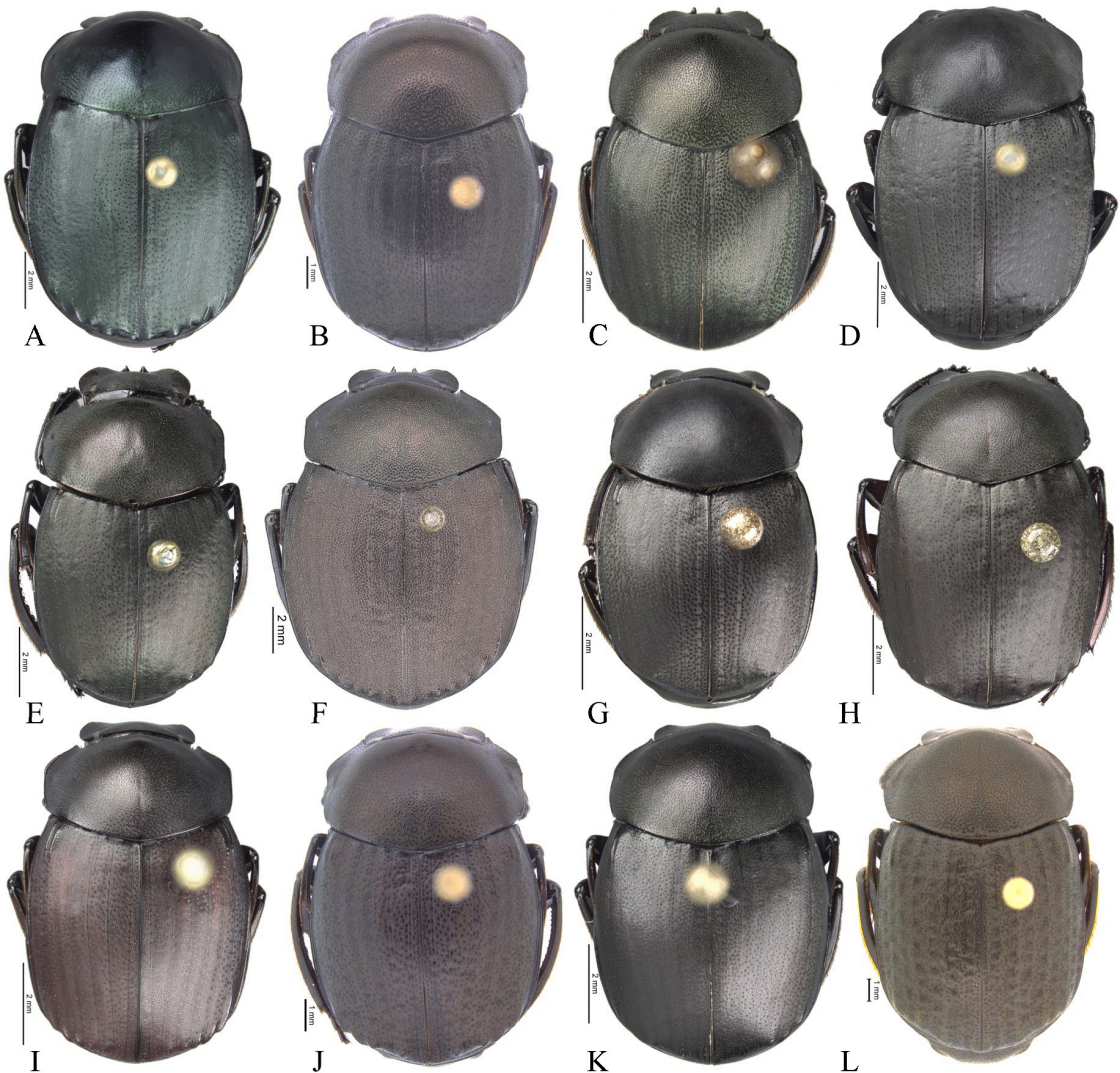
Habitus of *Deltohyboma*. (A) *aequinoctiale* species-group. (B) *aspericolle* species-group. (C) *aspericolle* species-group. (D) *barbipes* species-group. (E) *barbipes* species-group. (F) *bidentatum* species-group. (G) *femorale* species-group. (H) *genieri* species-group. (I) *gilli* species-group. (J) *granulatum* species-group. (K) *guyanense* species-group. (L) *irroratum* species-group.

**Fig 4:**
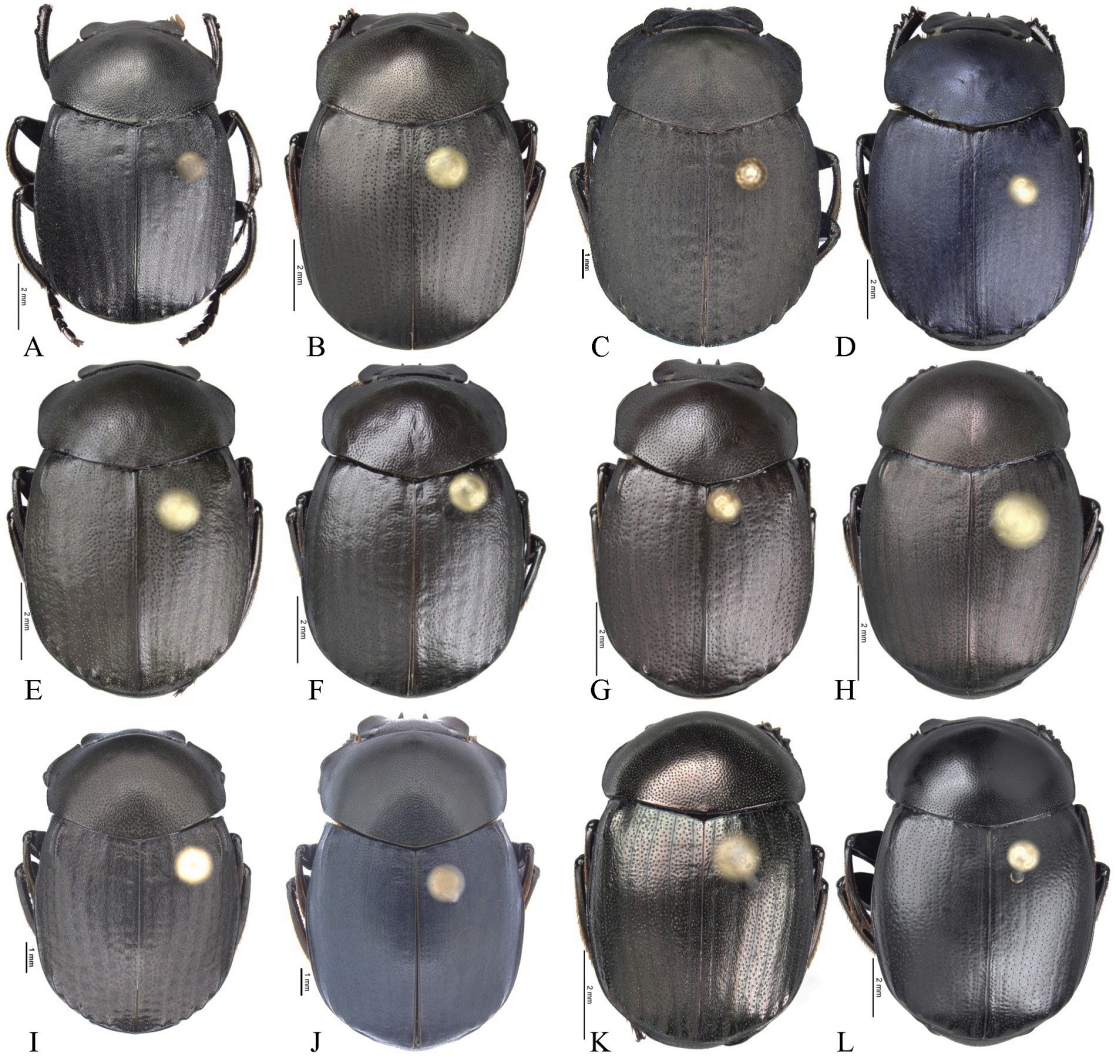
Habitus of *Deltohyboma*. (A) *komareki* species-group. (B) *lindemannae* species-group. (C) *morbillosum* species-group. (D) *parile* species-group. (E) *parile* species-group. (F) *plebejum* species-group. (G) *plebejum* species-group. (H) *septemstriatum* species-group. (I) *sextuberculatum* species-group. (J) *submetallicum* species-group. (K) *susanae* species-group. (L) *Deltochilum inesae* **sp. nov.** (*incertae sedis*).

**Fig 5:**
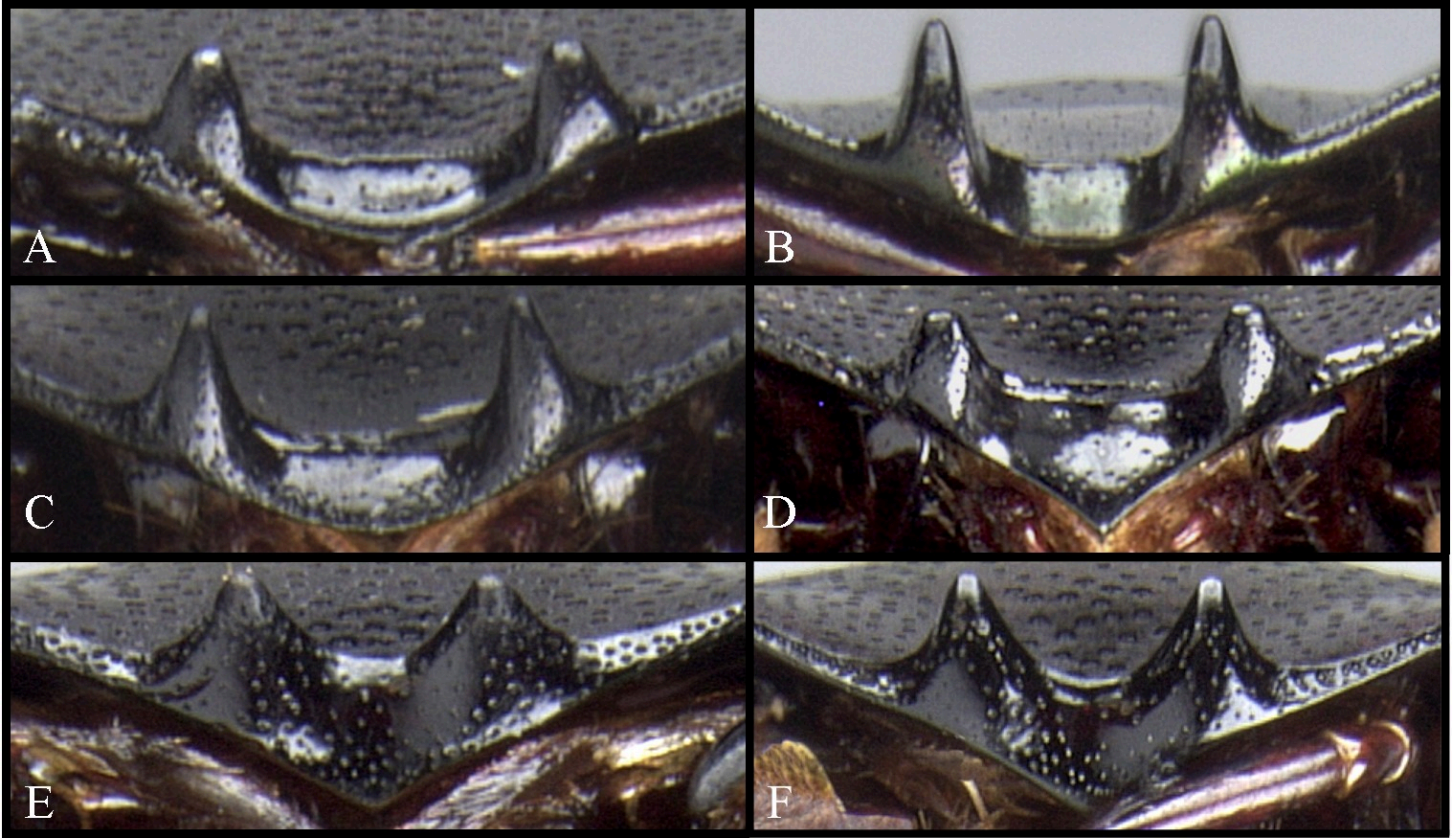
Anterior margin of the clypeus, between clypeal teeth of *Deltohyboma*. (A) *septemstriatum* species-group. (B) *aequinoctiale* species-group. (C) *guyanense* species-group. (D) *submetallicum* species-group. (E) *barbipes* species-group. (F) *barbipes* species-group.

**Body** (Figs 3 and 4). Small to large species, length 6.1–17 mm; humeral width 3.9–9.4 mm. Colour highly variable, but never metallic. Commonly dark brown or black. All punctures umblilicate. Punctures highly variable in sized and density. **Head**. Approximately 1.5x as wide as long. Dorsally, eyes small to large, inter-ocular distance seven to 20 times width of one eye. Anterior margin of the clypeus with two upturned teeth, each tooth bearing dorsal tuft of setae. Clypeal median emargination narrowly or broadly U-shaped. Clypeal teeth separated by less than a basal width of a tooth to seven times basal width of a tooth. Anterior margin of the clypeus, between clypeal teeth, with the following variations: 1) concave and regular, not expanded posteriorly (Fig 5A); 2) concave and expanded posteriorly, but not into triangular shape (Fig 5B and 5C); 3) concave and expanded posteriorly into triangular shape (Fig 5D) or 4) flat and expanded posteriorly into triangular shape (Fig 5E and 5F).

#### Pronotum

**Fig 6:**
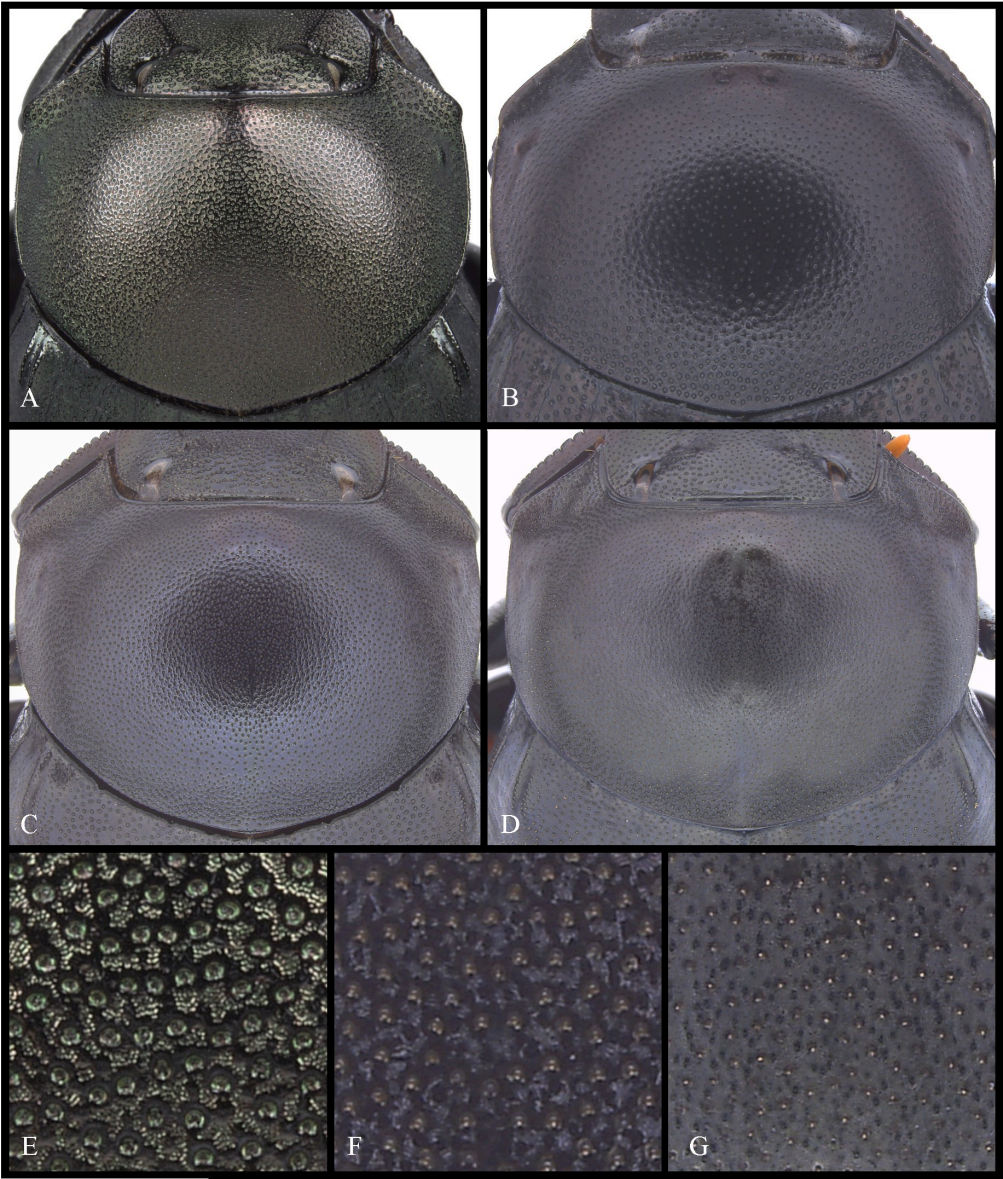
Pronotum (A-D) and microsculpture of pronotal disc showing the shiny points mixed with the punctures (E-G) of *Deltohyboma*. (A) pronotum with irregular shiny points, shiny points contiguous between them and/or with external margin of punctures. (B) pronotum without shiny points. (C) pronotum with irregular shiny points, shiny points contiguous between them and/or with external margin of punctures. (D) pronotum with regular shiny points, shiny points separated between them and from external margin of punctures. (E) pronotal disc of (A). (F) Pronotal disc of (C). (G) Pronotal disc of (D).

**Fig 7:**
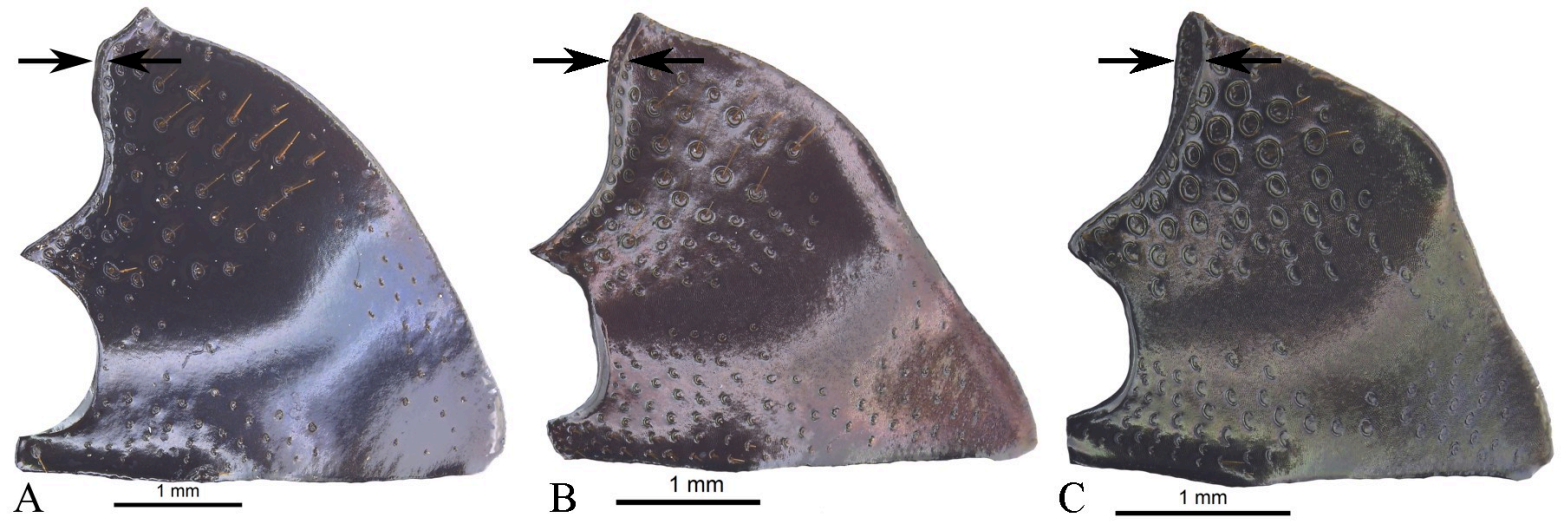
Hypomera of *Deltohyboma*. arrows showing the internal margin of anterior angle. (A) *aequinoctiale* species-group. (B) *guyanense* species-group. (C) *submetallicum* species-group.

Anterior angle acute, edge between anterior and medial-lateral angle concave to almost straight. Medial-lateral angle rounded to strongly projected; edge between medial-lateral and posterior angle subrounded. With (Fig 6A, 6C and 6D) or without (Fig 6B) shiny points mixed with the punctures, if present, well defined (Fig 6D and 6G) or irregular (Fig 6A, 6C, 6E and 6F). Irregular shiny points contiguous between them and/or external margin of punctures (Fig 6A, 6C, 6E and 6F). Shiny points variable in size and quantity. Punctures highly variable in size and density, frequently posterior-basal punctures largest and on disc smallest. **Hypomera**. Hypomeral carina absent. Internal margin with the following variations: 1) regular, not enlarged towards anterior angle (Fig 7A, arrows), 2) enlarged towards anterior angle (Fig 7B, arrows) or 3) strongly enlarged towards anterior angle (Fig 7C, arrows). Anterior punctures largest, medial area almost without punctures and posterior punctures extended and smallest.

#### Metaventrite

**Fig 8:**
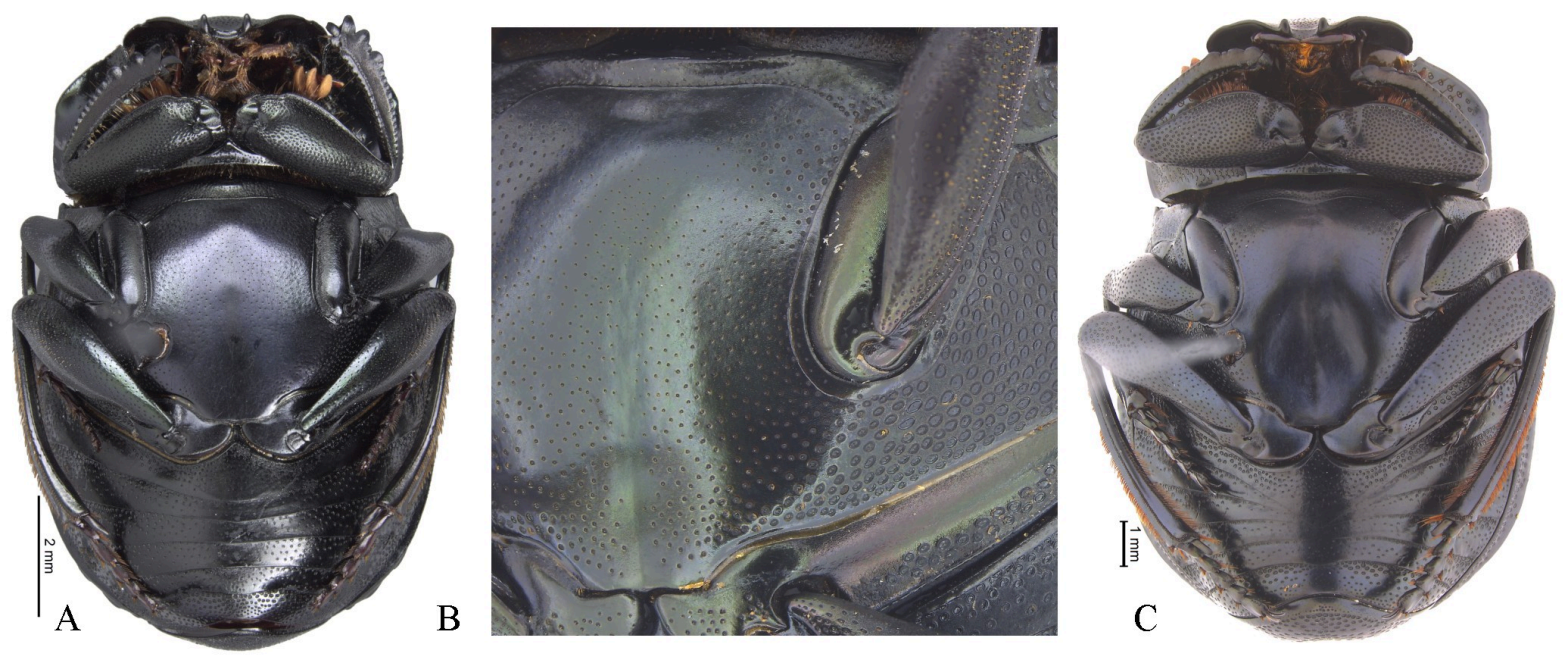
Body, ventral view (A, C), metaventrite (B) of *Deltohyboma*. (A) *guyanense* species-group. (B) *submetallicum* species-group. (C) *irroratum* species-group.

**Fig 9:**
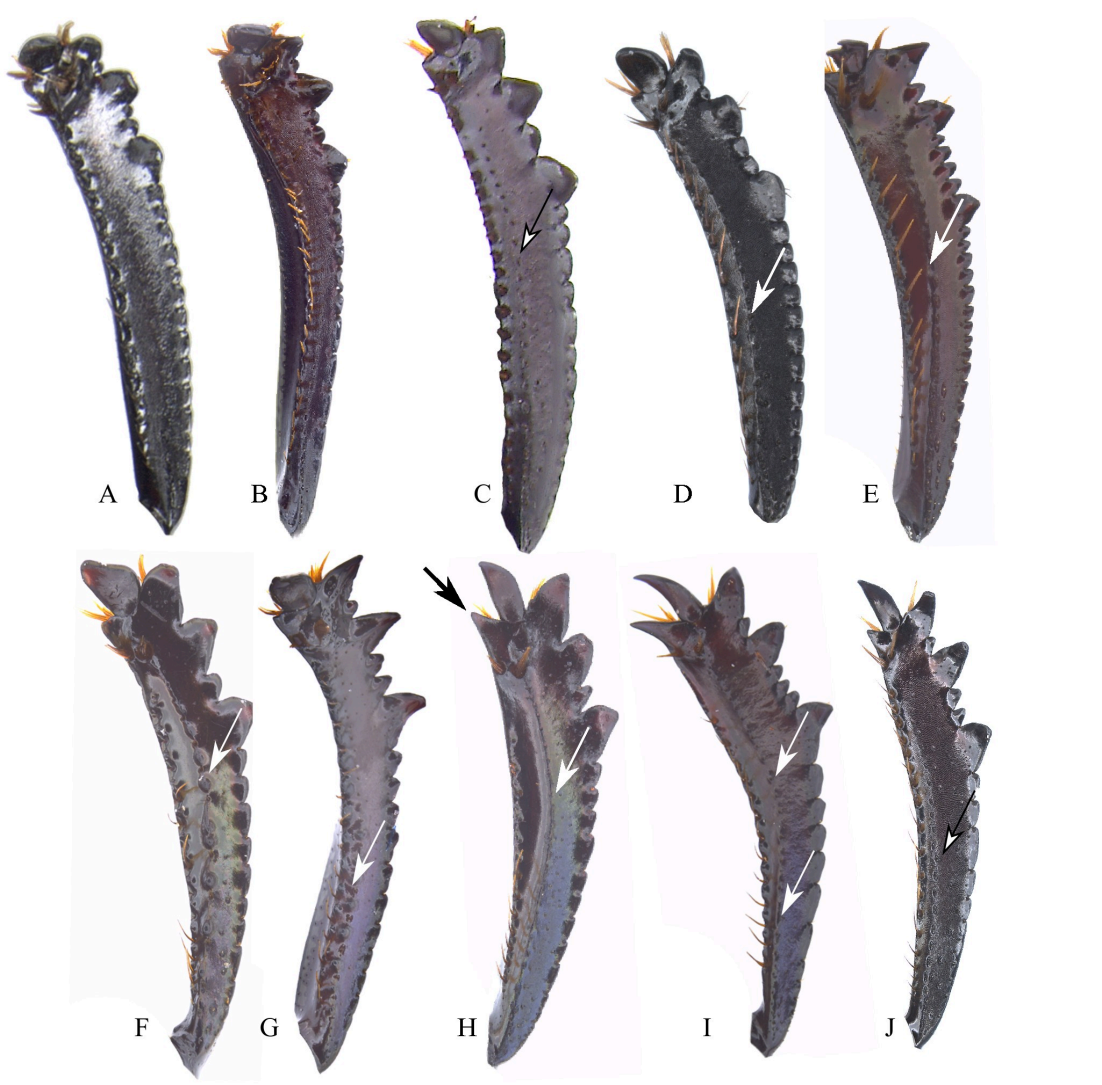
Protibia ventral view of *Deltohyboma*. White arrow showing tubercles (F, G, I) or carina (E, H), white arrow showing weak carina (D), black bordered white arrow showing punctures where the carina is found (C, J). Black arrow showing spiniform projection (H). (A) male without carina or tubercles. (B) male without carina or tubercles. (C) male without carina or tubercles, but with small punctures on where the carina is found. (D) male with weak carina. (E) male with carina. (F) male with tubercles. (G) male with tubercles only basally. (H) female with carina. (I) female with basal and apical carina, medially with tubercles. (J) female without carina or tubercles, but with large punctures where the carina is found.

**Fig 10:**
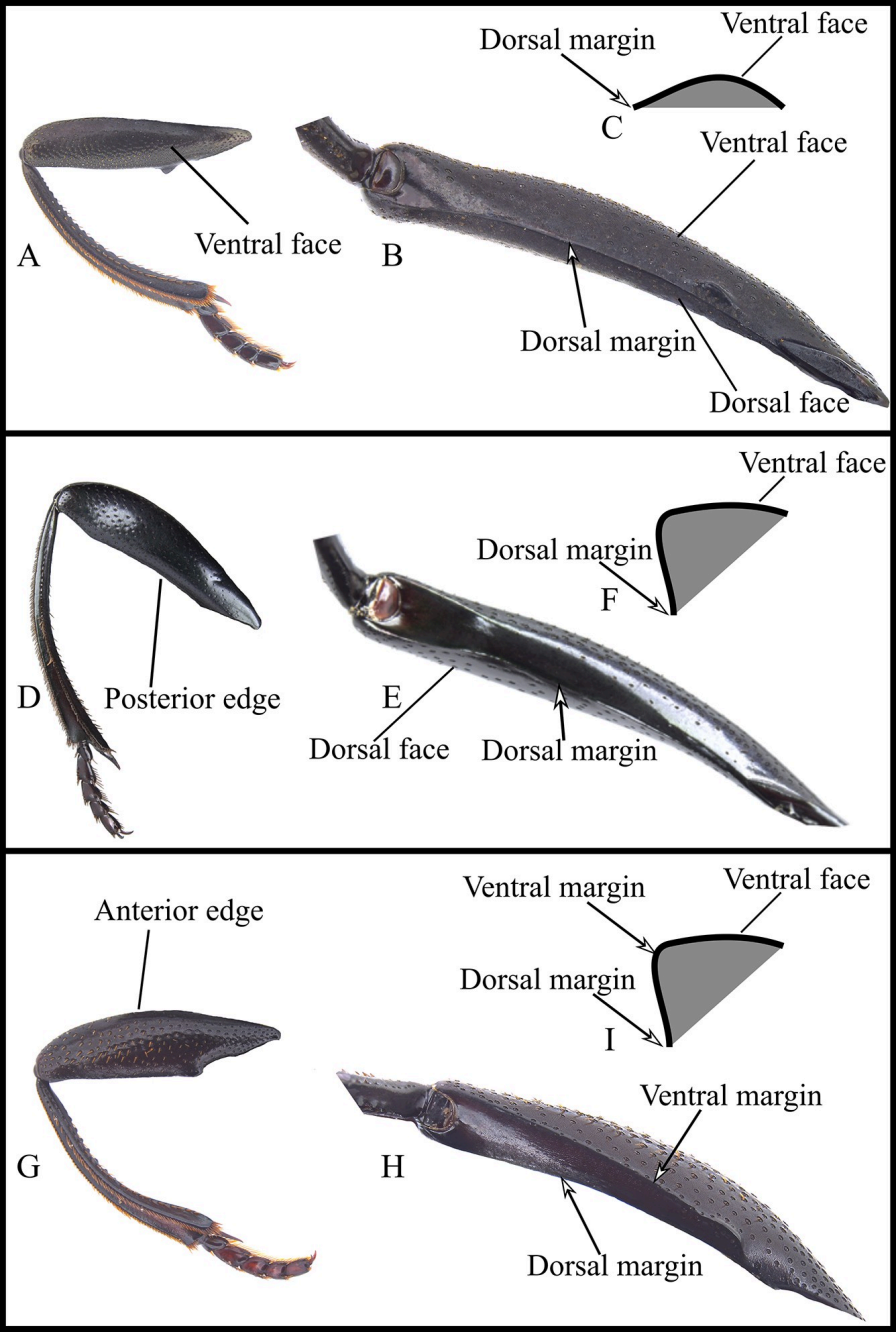
Three types of metafemur of *Deltohyboma*. (A-C) posterior edge with one margin, the posterior-dorsal, the ventral surface is continuous up to that posterior-dorsal margin. (D-E) posterior edge with one margin, the posterior-dorsal, the ventral surface on the posterior-ventral edge forming a decline of approximately 45° to that posterior-dorsal margin. (G-H) posterior edge with two margins, one ventral continued by a decline of 45° to the other, posterior-dorsal one. (A, D, G) ventral view hind leg. (B, E, H) caudal view metafemur. (C, F, I) schematic of transverse cut of metafemur.

**Fig 11:**
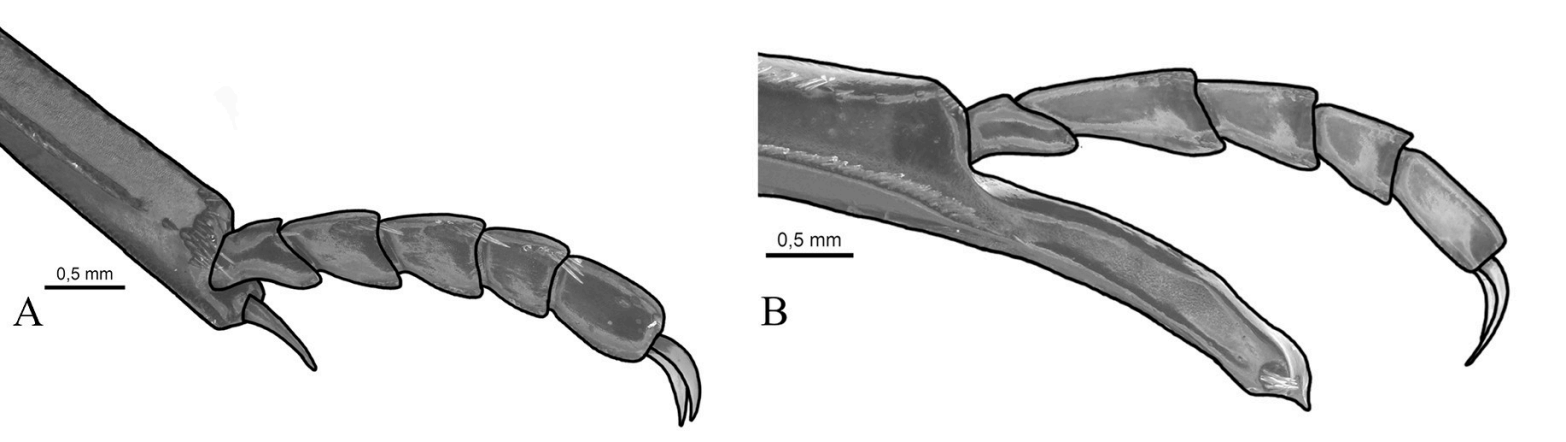
Apex of male metatibia and metatarsomeres of *Deltohyboma*. (A) *irroratum* species-group. (B) *aequinoctiale* species-group.

Disc without (Fig 8A) or with posterior excavation (Fig 8B and 8C), less commonly with two posterior excavations. Excavation, or the most basal excavation, occupying metaventral basal fourth (Fig 8B) to basal half (Fig 8C). If with two excavations, with small or large projected tubercle anterior to the most anterior excavation. If with one excavation, occupying metaventral basal fourth, commonly weak (Fig 8B). Disc with conspicuous, or rarely, inconspicuous punctures at 8x magnifications. Metaventral process commonly with anterior-lateral area bearing dense punctures, larger than disc punctures. Lateral lobes of metaventrite with largest punctures, separated by less than one diameter and equidistantly separated (Fig 8A). Mesepimerum commonly with extended punctures not fully closed and equidistantly separated. Metepisternum with slightly smaller punctures than those on lateral lobes of metaventrite and equidistantly separated. **Legs**. Protibia without tarsus (Fig 9). Protibia with three lateral teeth, in one species male with only two small teeth. Teeth on apical third almost equidistantly separated or proximal tooth more separated than the distal two. External margin with denticles, also between teeth. Internal margin with deep impressed setose punctures. Dorsal surface with two parallel carinae, one almost on the middle, bearing deeply impressed setose punctures, and reaching the apex of protibia; the second most external, curved on proximal tooth and reaching the apex of that tooth. Only in a few species, male internal margin of protibia expanded. Ventral surface of protibia, bearing tubercles (Fig 9F and 9G, arrow), weak carina (Fig 9D, arrow), strong carina (Fig 9H, white arrow), carina and tubercles (Fig 9I, arrows) or none of these (Fig 9A–9C and 9J); sometimes with the carina interrupted by punctures. If without carina or tubercles (Fig 9A), with small (Fig 9C, arrow) or with large (Fig 9J, arrow) punctures where the carina is found. Ventral surface of profemur with dense punctures separated almost equidistantly and before anterior margin, bent downward or not. Anterior margin of profemur with long setae. Mesocoxal axis slightly oblique. Ventral surface of meso- and metafemur with punctures separated almost equidistantly. Posterior edge of metafemur with one dorsal margin (Fig 10A–10F) or two margins (Fig 10G–10I), one ventral continued by a decline of 45° (Fig 10I) to the one other, dorsal one (Fig 10H and 10I). If with one margin with the following variations: 1) ventral surface of metafemur is continuous (Fig 10C) to the posterior-dorsal margin (Fig 10B and 10C) or 2) the ventral surface of metafemur on the posterior ventral edge forming a decline of approximately 45° (Fig 10F) to the posterior-dorsal margin (Fig 10E and 10F). Mesotibia with two spurs, unequal in length. Metatibia with one spur. Meso- and metatarsi flattened, almost identically shaped, however mesotarsi smaller than metatarsi. Metatarsomeres (Fig 11) I and IV subequal in length and shortest. Metatarsomeres III and V subequal in length. Metatarsomere II longest. Metatarsomere V elongated, longer than broad and IV not elongated, almost as long as broad. Metatarsomeres II and III may not be elongated, each almost as long as broad (Fig 11A) or be elongated, each longer than broad (Fig 11B).

#### Elytra

**Fig 12:**
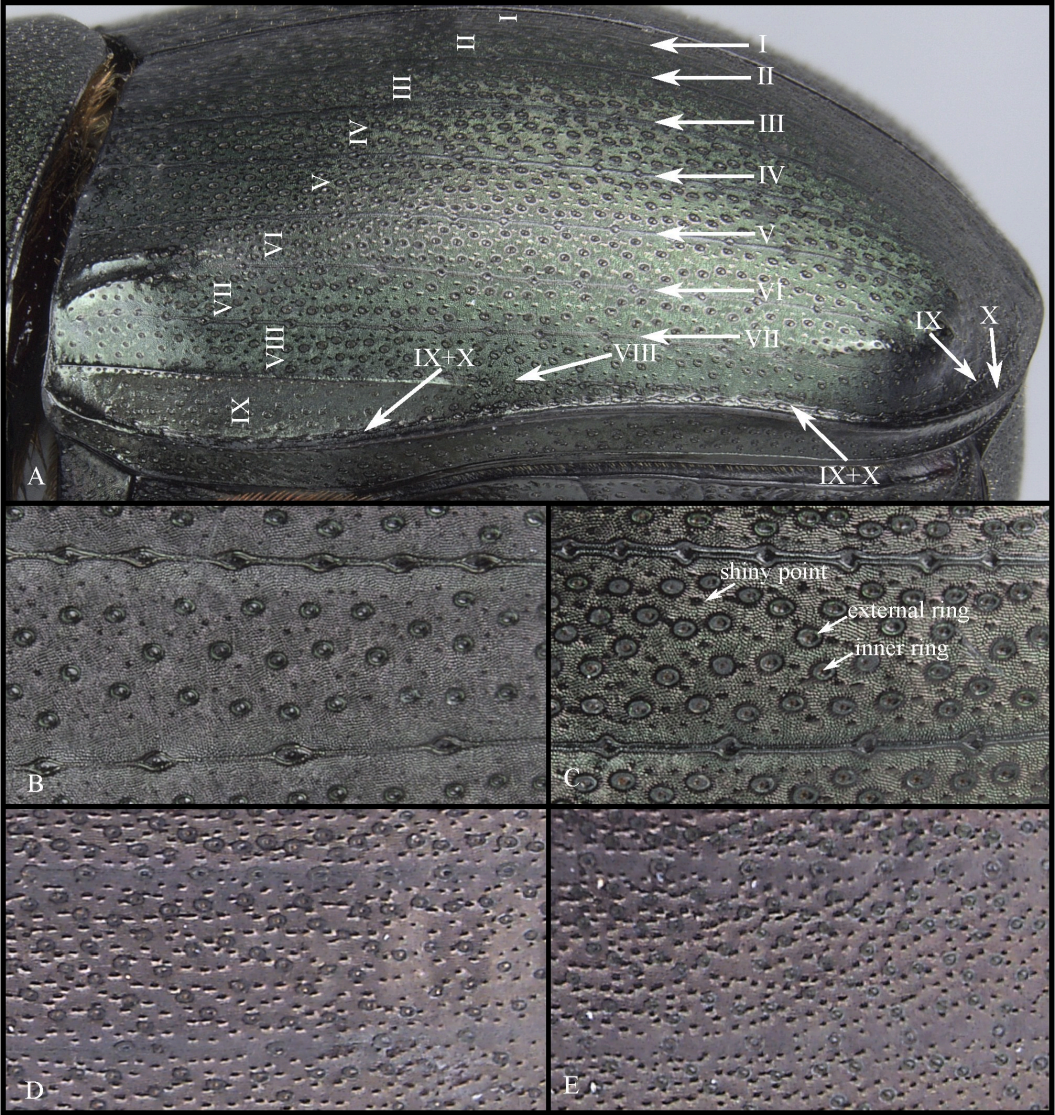
Elytra (A) and microsculpture of elytra, showing shiny points mixed with the punctures (B-E) of *Deltohyboma*. (A) lateral view, Roman numerals correspond to interstriae, Roman numerals with arrows correspond to striae. (B) microsculpture of elytra showing small dispersed shiny points. (C) microsculpture of elytra showing small, abundant shiny points, also external and internal margins of punctures. (D) microsculpture of elytra showing large and dispersed shiny points. (E) microsculpture of elytra showings large, abundant shiny points.

With pseudoepipleuron delimited by a sinuate carina present along whole elytral length (Fig 2A and 2B), carina adjacent to “stria” IX+X (Fig 12A). Humeral area with one (Figs 3F and 4C) or two basal tubercles (Figs 3A–3E, 3G–3L, 4A and 4B and 4D–4L), on interstriae VI or VI-VII respectively. Apically with tubercles, highly variable in quantity and development, but at least two tubercles on interstriae VI-VII or maximum six tubercles, on interstriae II-VII. Apical tubercles with following variation: commonly on interstriae 1) II-VII, 2) III-VII, 3) III, V-VII, 4) V-VII, and less commonly 5) VI-VII and 6) IV-VII. In variation “3” with all tubercles well developed or with III or III and V poorly developed; in variation “4” all well developed or with V poorly developed. Ninth interstria with basal carina, variable in length, reaching basal elytral third, almost reaching, reaching or surpassing a little the middle of the elytral length (Fig 2A and 2B). Very rarely not reaching elytral basal third and almost obliterated. With eleventh striae (including the epipleural one), stria IX apically separated from stria X, laterally fused here call “stria” IX+X (Fig 12A). Striae inconspicuous (Fig 12D and 12E) or conspicuous (Fig 12B and 12C), if inconspicuous with the following variations: 1) inconspicuous I-IX+X; 2) inconspicuous I-VII, conspicuous VIII-IX+X; 3) inconspicuous I-VII, conspicuous only apically VIII and IX+X conspicuous or 4) I-VIII inconspicuous, conspicuous IX+X. If conspicuous narrow or broad, all with almost the same width or III-VII consecutively narrower and more effaced. Stria VIII conspicuous apically only or apically and laterally; if conspicuous laterally not reaching, reaching or slightly surpassing the apex the carina of the ninth interstria, rarely reaching the elytral base. Interstriae with punctures variable in size and density and with shiny points mixed with the punctures (Fig 12B–12E); with a few (Fig 12B and 12C) or abundant (Fig 12D and 12E) shiny points; points variable in size, commonly smaller than inner ring of interstrial puncture (Fig 12B–12D), rarely subequal in size to interstrial puncture (Fig 12E). **Abdomen**. With VI ventrites. Ventrites with sparse punctures, laterally punctures largest and densest than medially. Pygidium almost the shape of a Reuleaux triangle, with one of the angles broader than the other two. Slightly convex. Apical margin wider than lateral ones. Pygidium with subrounded or transversally extended punctures; with or without shiny points mixed with punctures, shiny points few or abundant, minute to small, smaller than inner ring of a pygidial puncture.

#### Male genitalia (Figs 13–17)

**Fig 13:**
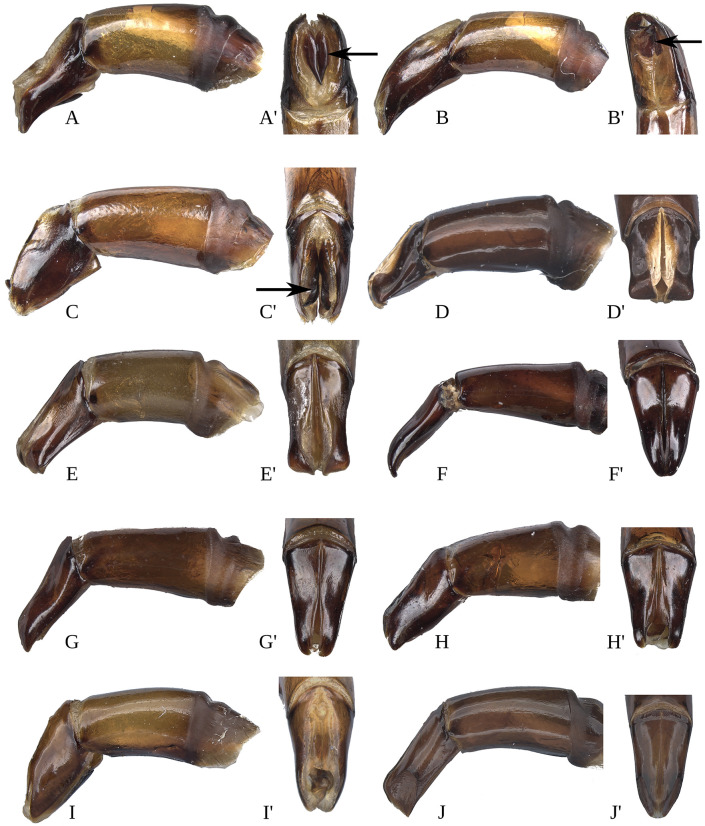
Aedeagus lateral view and paramera dorsal and/or ventral view of *Deltohyboma*. (A) *aequinoctiale* species-group, (A’) same, paramera ventral view, arrow showing the sclerotised paired structures. (B) *aequinoctiale* species-group, (B’) same, paramera ventral view, arrow showing the sclerotised paired structures. (C) *aequinoctiale* species-group, (C’) same, paramera dorsal view, arrow showing the sclerotised paired structures. (D) *aspericolle* species-group, (D’) same, paramera dorsal view. (E) *aspericolle* species-group, (E’) same, paramera dorsal view. (F) *barbipes* species-group, (F’) same, paramera dorsal view. (G) *barbipes* species-group, (G) same, paramera dorsal view. (H) *barbipes* species-group, (H’) same, dorsal view. (I) *bidentatum* species-group, (I’) same, paramera dorsal view. (J) *femorale* species-group, (J’) same, paramera dorsal view.

**Fig 14:**
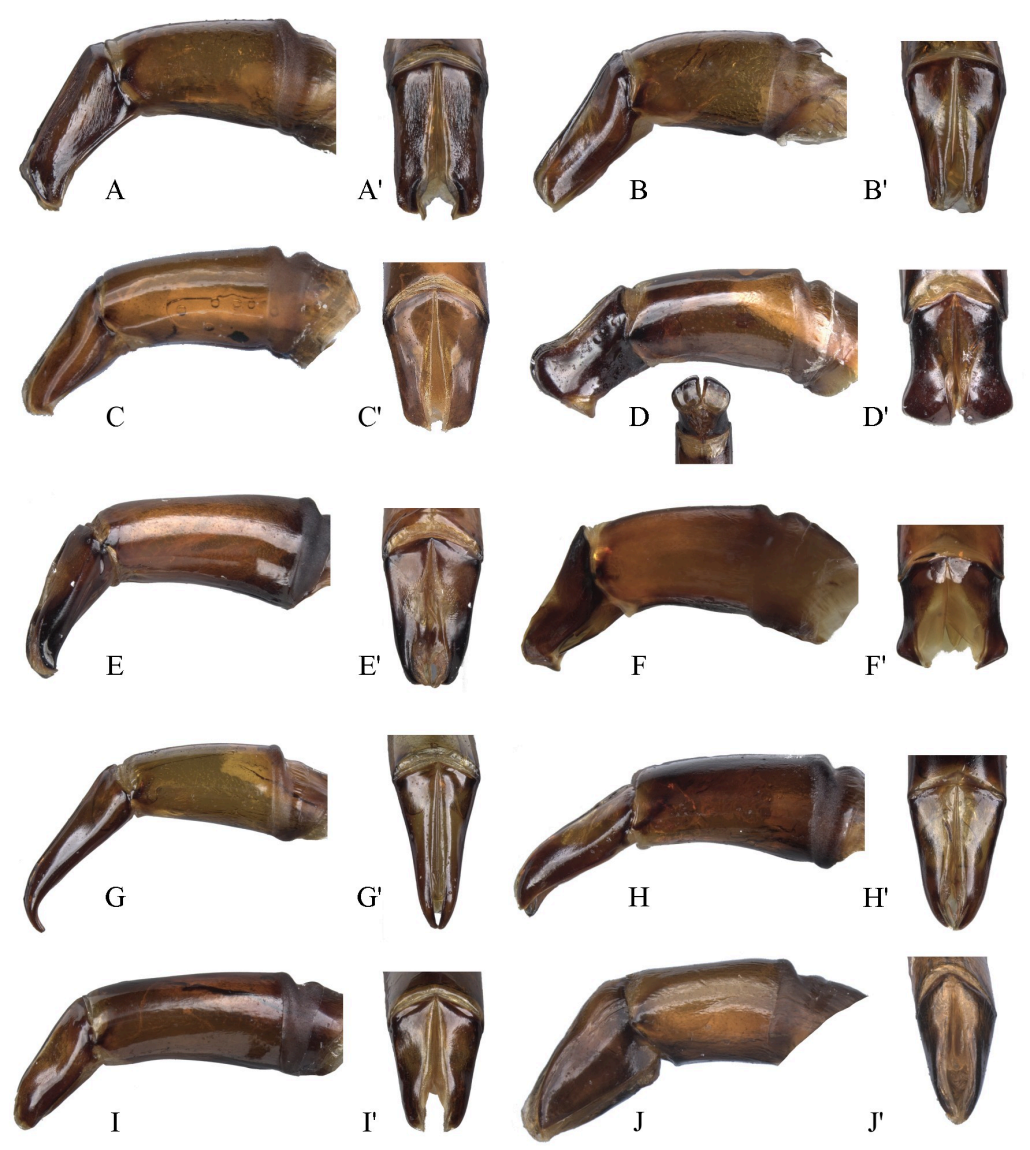
Aedeagus lateral view and paramera dorsal view of *Deltohyboma*. (A) *genieri* species-group, (A’) same, paramera dorsal view. (B) *gilli* species-group, (B’) same, paramera dorsal view. (C) *granulatum* species-group, (C’) same, paramera dorsal view. (D) *guyanense* species-group, (D’) same, paramera dorsal and ventral view. (E) *irroratum* species-group lateral view, (E’) same, paramera dorsal view. (F) *komareki* species-group, (F’) same, paramera dorsal view. (G) *lindemannae* species-group, (G’) same, paramera dorsal view. (H) *lindemannae* species-group, (H’) same, paramera dorsal. (I) *lindemannae* species-group, (I’) same, paramera dorsal view. (J) *morbillosum* species-group, (J’) same, paramera dorsal.

**Fig 15:**
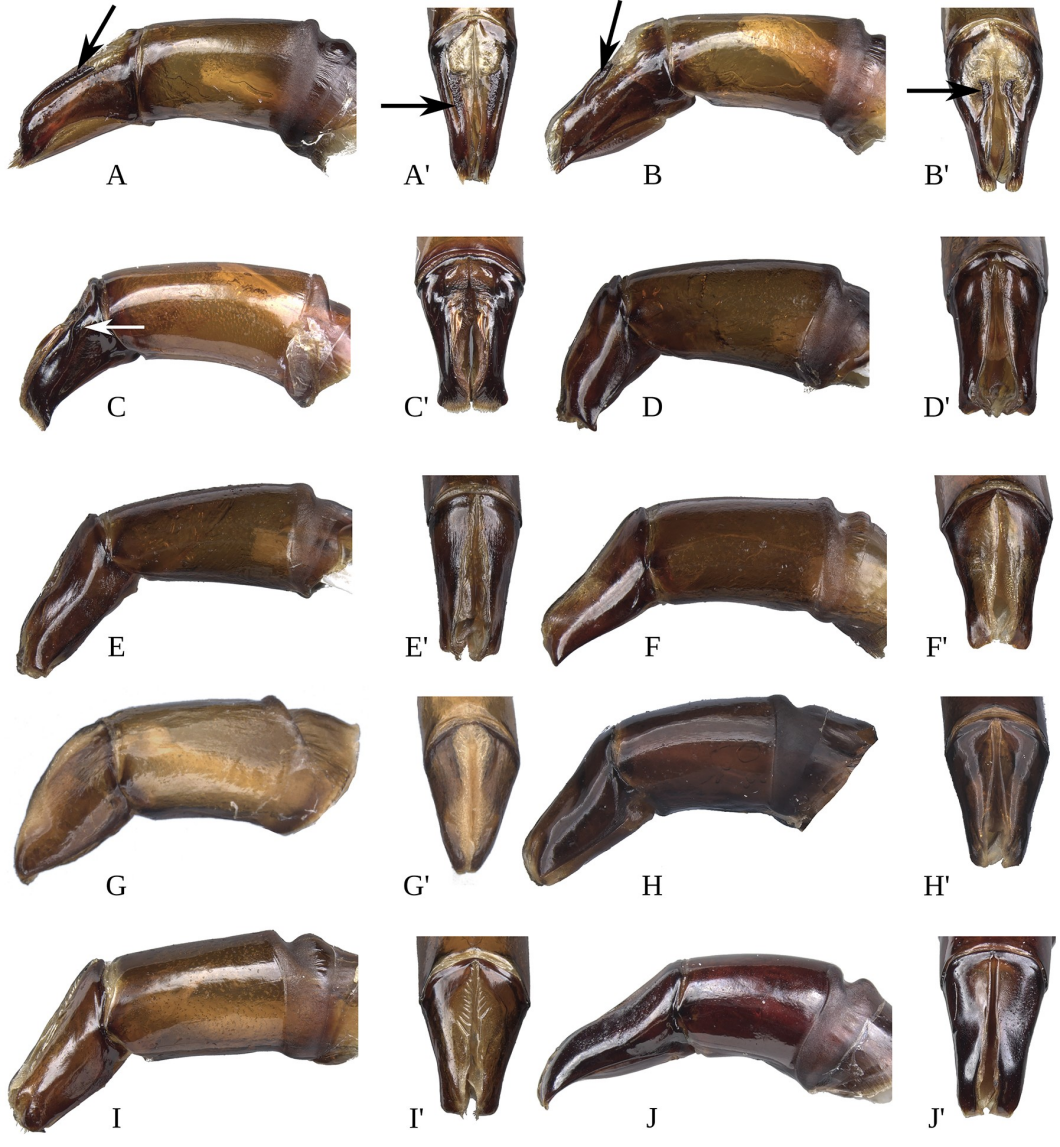
Aedeagus lateral view and paramera dorsal view of *Deltohyboma*. (A) *parile* species-group, (A’) same, paramera dorsal view, arrows showing the sclerotised paired structures. (B) *parile* species-group, (B’) same, paramera dorsal view, arrows showing the sclerotised paired structures. (C) *parile* species-group, arrow showing the strong basal sinuation, (C’) same, paramera dorsal view. (D) *plebejum* species-group, (D’) same, paramera dorsal view. (E) *plebejum* species-group, (E’) same, paramera dorsal view. (F) *septemstriatm* species-group, (F’) same, paramera dorsal view. (G) *sextuberculatum* species-group, (G’) same, paramera dorsal view. (H) *submetallicum* species-group, (H’) same, paramera dorsal view. (I) *susanae* species-group, (I’) same, paramera dorsal view. (J) *Deltochilum inesae* **sp. nov.** (*incertae sedis*), (J’) same, paramera dorsal view.

**Fig 16:**
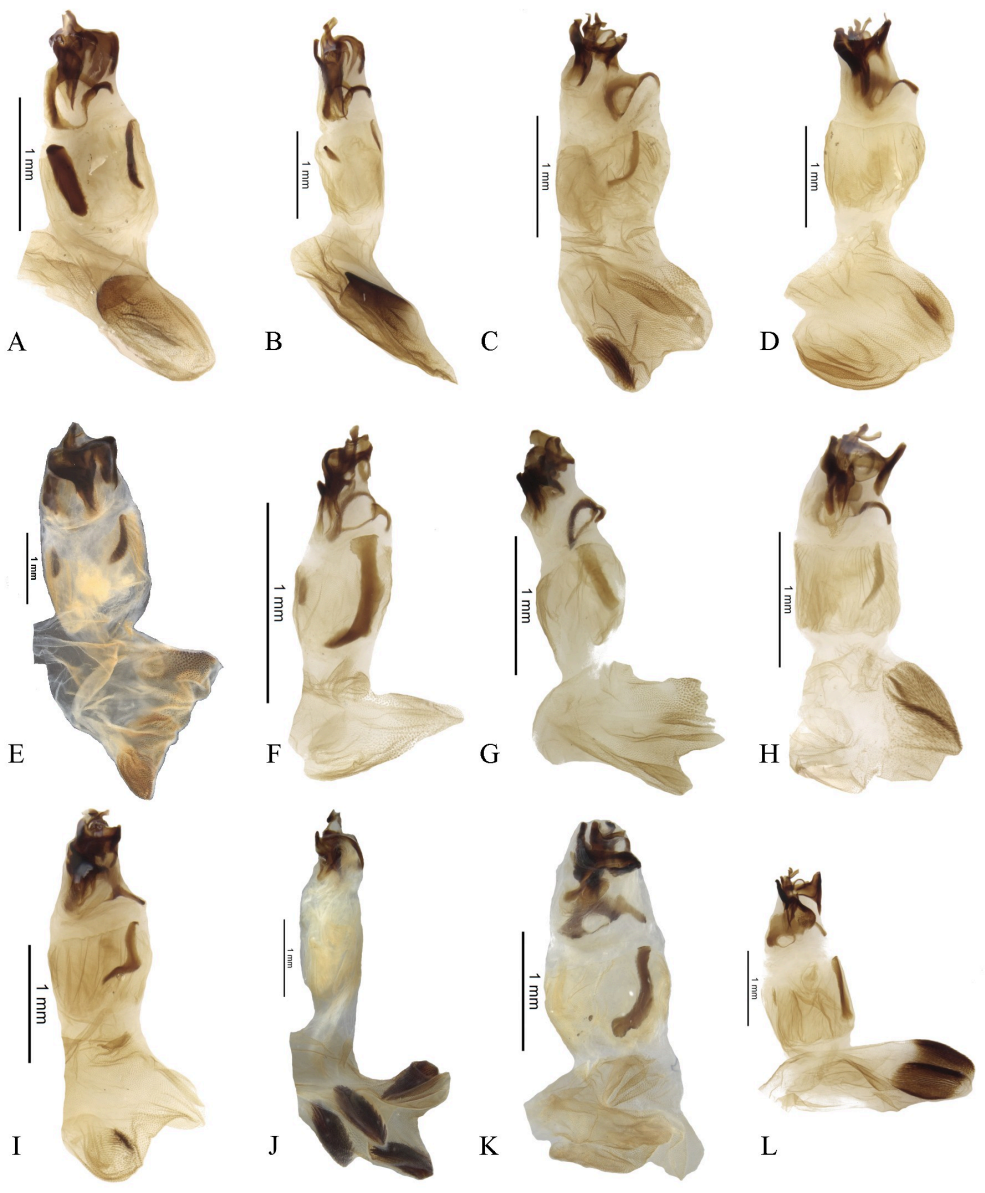
Endophallus of *Deltohyboma*. (A) *aequinoctiale* species-group. (B) *aequinoctiale* species-group. (C) *barbipes* species-group. (D) *barbipes* species-group. (E) *bidentatum* species-group. (F) *femorale* species-group. (G) *genieri* species-group. (H) *gilli* species-group. (I) *guyanense* species-group. (J) *irroratum* species-group. (K) *komareki* species-group. (L) *lindemannae* species-group.

**Fig 17:**
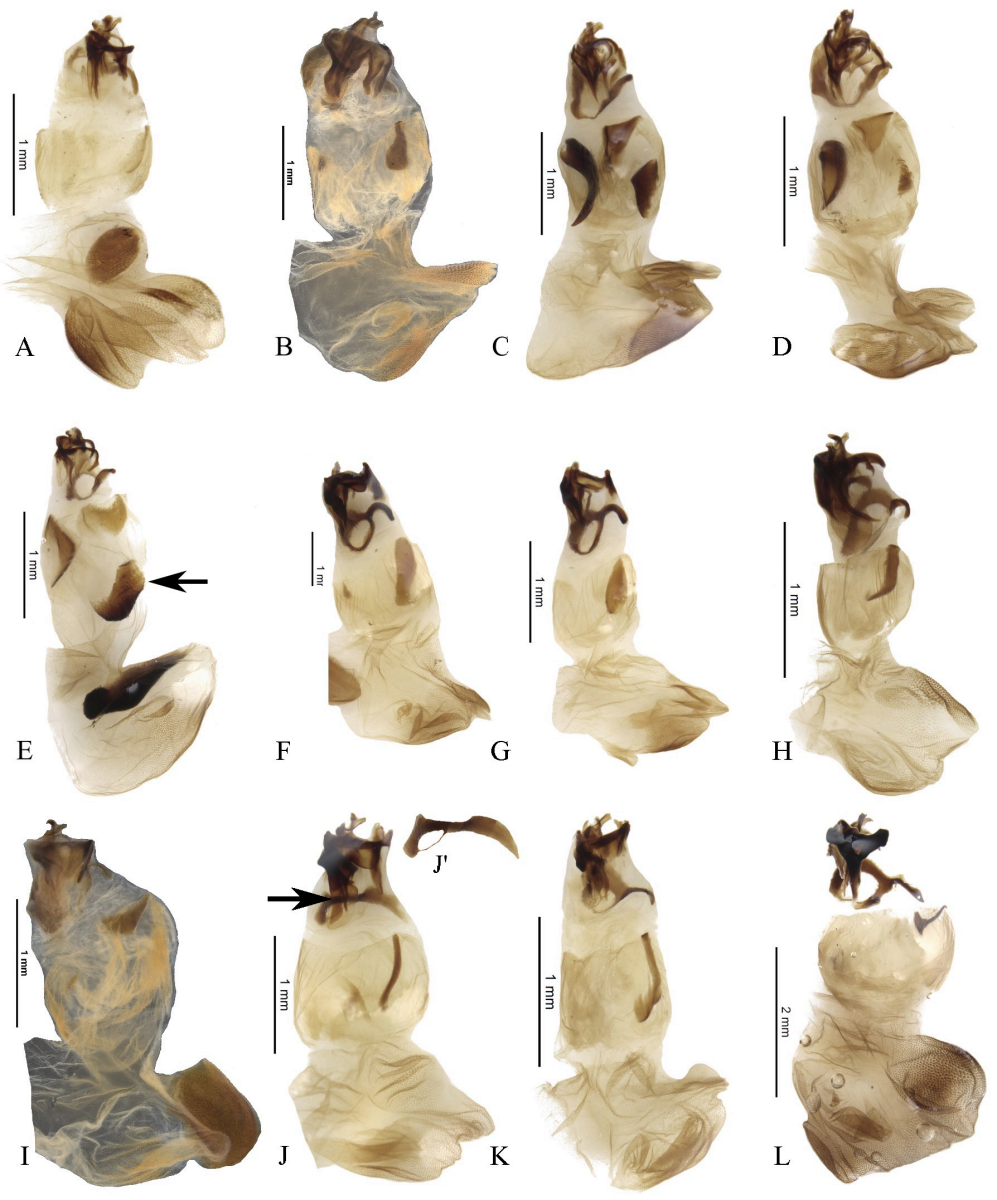
Endophallus of *Deltohyboma*. (A) *lindemannae* species-group. (B) *morbillosum* species-group. (C) *parile* species-group. (D) *parile* species-group. (E) *parile* species-group, arrow showing the raspule on medial area. (F) *plebejum* species-group. (G) *plebejum* species-group. (H) *septemstriatum* species-group. (I) *sextuberculatum* species-group. (J) *submetallicum* species-group, arrow showing the plate-shape endophallite “boot”-shaped, (J’) same, basal circular shape endophallite. (K) *susanae* species-group. (L) *Deltochilum inesae* **sp. nov.** (*incertae sedis*).

Paramera symmetrical. Commonly, paramera subtriangular; subequal or shorter than phallobase. Paramera simple or with dorsal sulcus (Fig 14A and 14A’), apical cleft (Fig 13J and 13J’), dorsal-apical notch thin (Figs 14E’, 14H’, 15I and 15I’), ventral-apical denticles (Fig 15D and 15E), ventral-apical setae (Figs 13A–13C, 15A–15C). Membrane between the paramera with dorsal (Figs 13C’, 15A, 15A’, 15B and 15B’, arrows) and/or ventral (Fig 13A’ and 13B’, arrows) sclerotised paired structures, dorsal sclerotised paired structures fused (Fig 15A, 15A’, Fig 15B and 15B’, arrows) or not (Fig 13C’, arrow) with the paramera. Endophallus always with three apical endophallites (Figs 1A, 16 and 17) 1) elongate, 2) basal with circular-shaped and 3) plate-shape. Medial area of endophallus (Figs 16 and 17) with or without one or two endophallites, rarely with three or raspules in that area. Sub-medial area with or without raspules (one or more). Shape of the endophallites as well as raspules and number of raspules highly variable. **Female genitalia**. Bursa copulatrix simple, pigmented, with horizontal folds or sclerotised. Base of the duct of the spermatheca sclerotised or not. Spermatheca well sclerotised “sickle”-shaped, with two basal spatulate expansions or not.

#### Secondary sexual dimorphism

**Fig 18:**
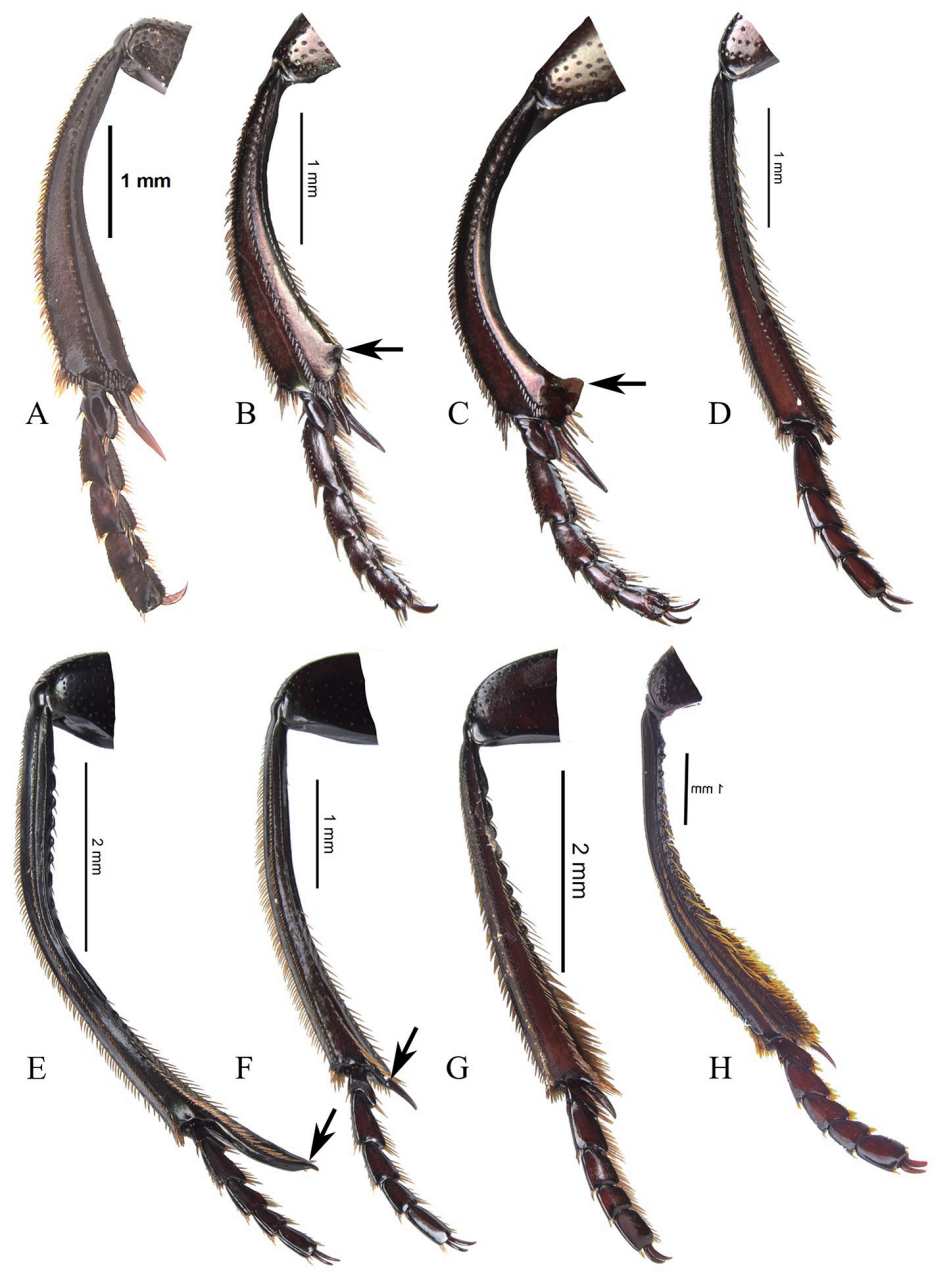
Meso- and metatibiae of *Deltohyboma*. (A) mesotibia female. (B) mesotibia male, arrow showing small denticle. (C) mesotibia male, arrow showing large spatulate expansion. (D) metatibia female. (E) metatibia male with basal internal tubercles, arrow showing spur fused. (E) metatibia male with internal carina, arrow showing spur articulated. (G) metatibia male with internal tubercles occupying almost metatibial length. (H) metatibia male with internal tubercles and large setae.

Male. Remarkably variable. In few species distance between clypeal teeth larger in males than females. Protibial spur broad (Fig 9C), broad and foliaceus (Fig 9F) or broad and apically bifid (Fig 9E). Protibia subequal to that of female or with one or some combination of the following characters: curved, broad apically, more flattened, internal margin expanded, with tubercles. Meso- and/or metatrochanters subequal to that of female or with setae, dentiform process or sinuated. Mesofemur subequal to that of female or on posterior edge with one of the following characters: sinuate, bearing setae, with small or large expansion, with small or large tubercle. Mesotibia more curved and apically broader (Fig 18B and 18C) than those of females (Fig 18A) and apex with a small or large (Fig 18C, arrow) spatulate expansion or with a small denticle (Fig 18B, arrow).

**Fig 19:**
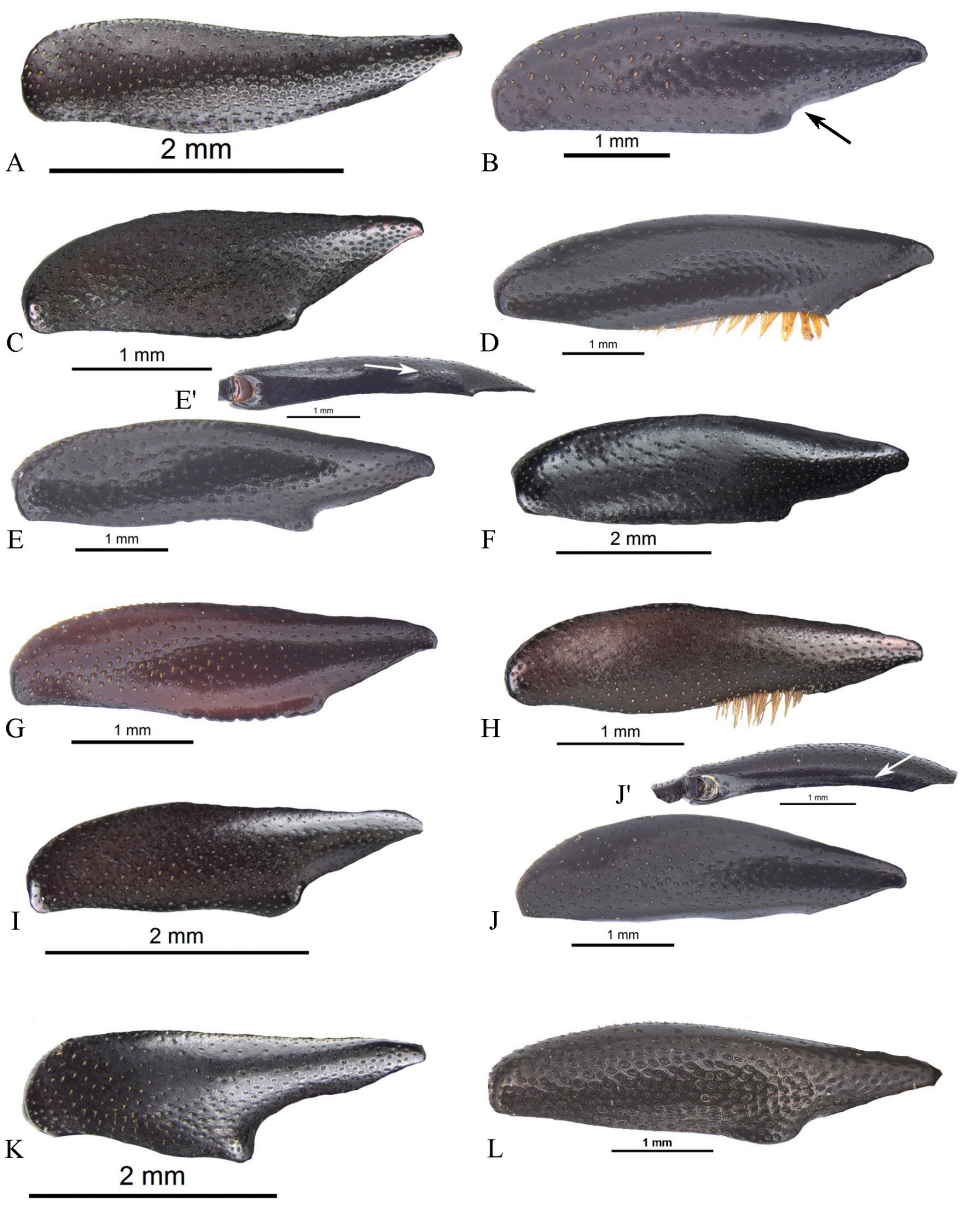
Metafemur ventral view and caudal view, female (A) male (B-L) of *Deltohyboma*. (A) typical female metafemur. (B) *aspericolle* species-group, arrow showing the basal strong steep tapering. (C) *aspericolle* species-group. (D) *barbipes* species-group. (E) *barbipes* species-group, (E’), same in caudal view, arrow showing the denticle on posterior-ventral margin. (F) *barbipes* species-group. (G) *femorale* species-group. (H) *genieri* species-group. (I) *gilli* species-group. (J) *granulatum* species-group, (J’), same in caudal view, arrow showing the weak carina on posterior edge. (K) *guyanense* species-group. (L) *komareki* species-group.

**Fig 20:**
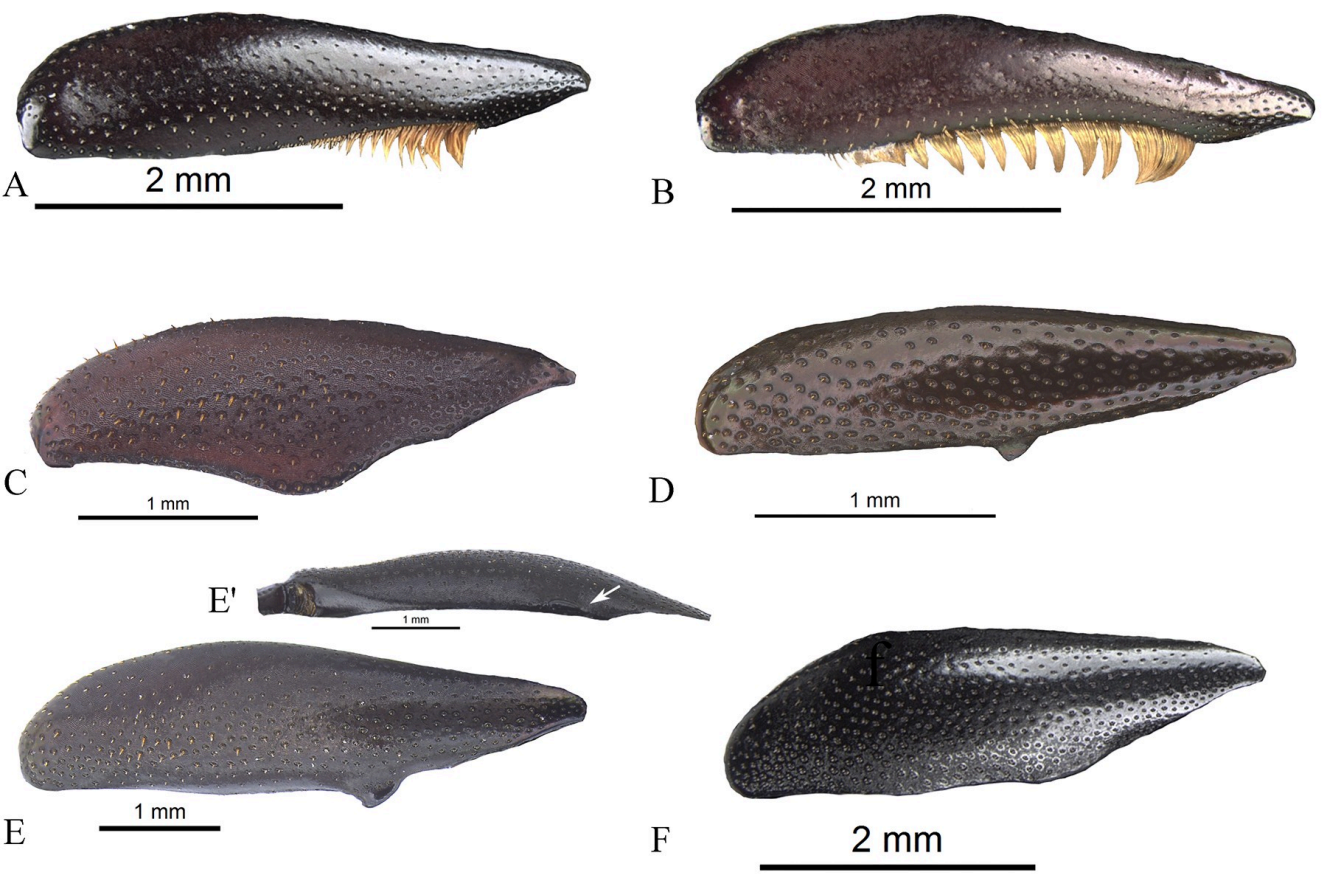
Metafemur ventral view and caudal view male of *Deltohyboma*. (A) *lindemannae* species-group. (B) *lindemannae* species-group. (C) *septemstriatum* species-group. (D) *sextuberculatum* species-group. (E) *submetallicum* species-group, (E’), same in caudal view, arrow showing the denticle on posterior-dorsal margin. (F) *Deltochilum inesae* **sp. nov.** (*incertae sedis*).

**Fig 21:**
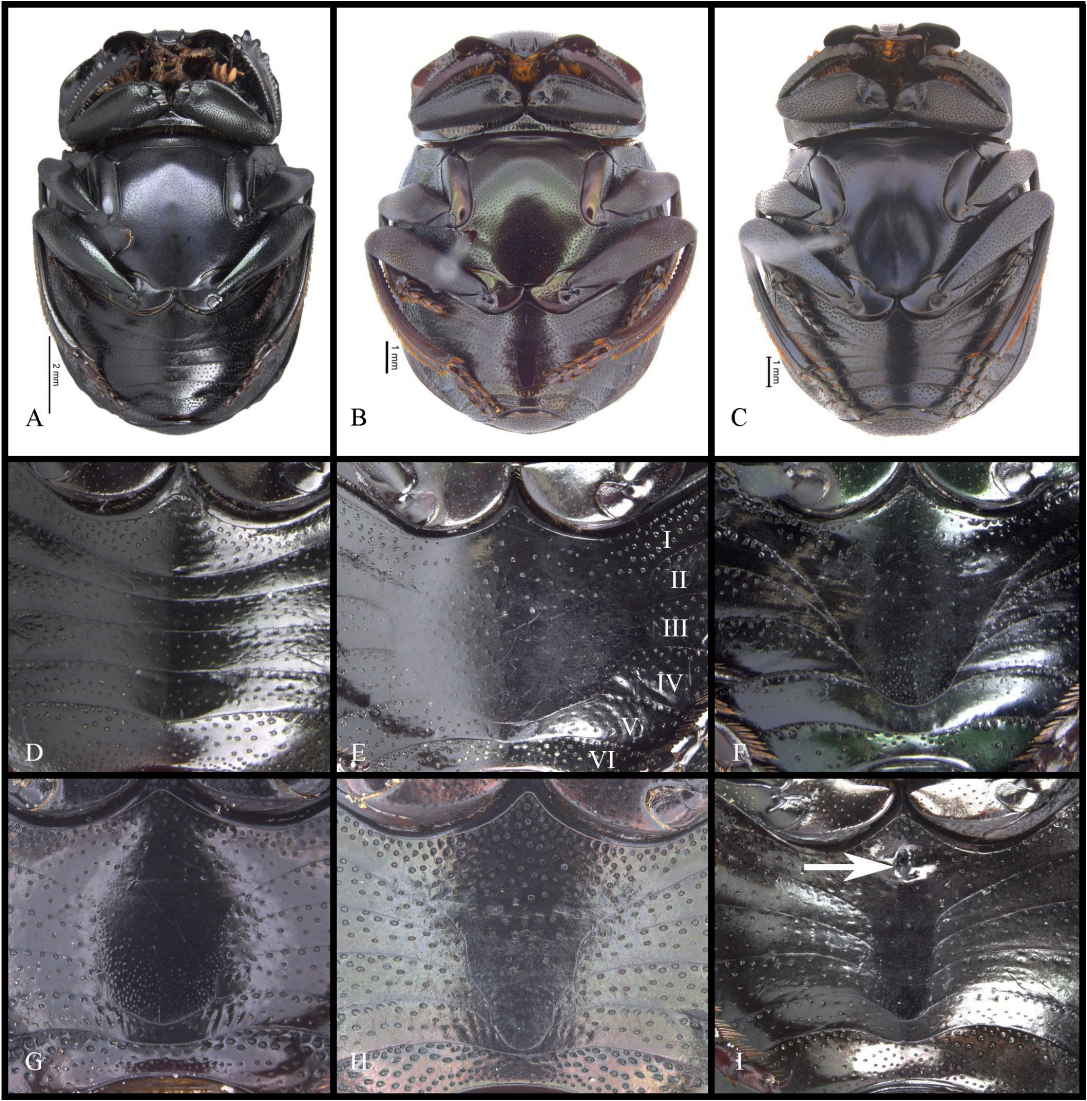
Ventral view and ventrites of *Deltohyboma*. (A) female (B-C) male. Ventrites of males (D-I). (E) Roman numerals correspond to the ventrite number. (I) Arrow showing the circular orifice on ventrite I.

Metafemur (Figs 19 and 20) subequal to that of females (Fig 19A), more curved than females and/or on posterior edge with one of the following characters: setae (Figs 19D, 19H, 20A and 20B), carina (Fig 19J and 19J’), carina and setae, small or large denticle (Figs 19E, 19K and 20E), with basal steep tapering (Fig 19B, arrow), broad basal sub-quadrate expansion (Fig 19I), broad medial serratulate expansion (Fig 19G), broad medial expansion (Fig 20C). Metatibia more curved and apically broader (Fig 18E–18H) than females (Fig 18D) and/or with one or combination of some of following characters: internal margin with tubercles (Figs 15H and 18G), strong carina (Fig 18F), long and dense setae (Fig 18H), insertion of the spur elongated (Figs 11B and 18E, arrow) or not (Figs 11A and 18F–18H), spur fused (Figs 11B and 18E, arrow) or not to insertion (Figs 11A and 18F, arrow). Metaventrite subequal to that of female (Fig 8A) or with stronger posterior excavation than female (Fig 8B and 8C) and in some species, with small (Fig 2B, arrow) or strong tubercle anterior to excavation or with two basal small tubercles. Abdomen with ventrite I medially expanded (Fig 21B, 21C and 21E–21I) (expansion covering margins of other ventrites) or not (Fig 21D). If expanded, reaching from distal margin of ventrite II to surpassing the distal margin of V; expansion variable in shape and width (Fig 21B, 21C and 21E–21I), also with ventrites V and VI strongly narrow medially (Fig 21E–21I). If not expanded or slightly expanded (almost reaching distal margin of ventrite III) ventrites V and VI only slightly narrow medially and V subequal to narrower than VI (Fig 21C and 21D). Ventrite I bearing (Fig 21I, arrow) or not an orifice (Fig 21B–21H); orifice variable in size and shape.

#### Female

Protibial spur thin and spiniform (Fig 9H–9J). Inner apical angle of protibia with spiniform projection (Fig 9H, black arrow) or with spiniform projection thinner and longer than males. Medially, ventrite I not expanded posteriorly (Fig 21A), broader than ventrite II; ventrites II-V almost with the same width, only V slightly broadest. Ventrites V-VI not strongly narrow medially and VI broader than V (Fig 21A). In species with males with posterior excavation on metaventrite, excavation weaker than males.

#### Distribution

*Deltohyboma* is widely distributed in the Neotropical region, absent from the Antillean subregion. Within the South American Transition Zone present only in Paramo province and with few species on Mexican Transition Zone (Chiapas Highlands and Sierra Madre del Sur provinces). Mos species inhabit South America. The northernmost records are in the Mesoamerican dominion, Veracruzan province, approximately 18°N, and the southernmost records are in the Chacoan dominion, Pampean province approximately 32°S.

#### Remarks

*Deltochilum* (*Deltohyboma*) males commonly bear an orifice on ventrite I. This orifice is present in some species-groups and, within those species-groups, some species may bear the orifice whereas others do not. Furthermore, although only observed for a few species, some individuals of a species possess the orifice whereas others do not. Pluot-Sigwalt [65] studied the tegumentary glands in Scarabaeidae, which included two species of *Deltohyboma* that do not bear the orifice; however, one species (*D*. *guyanense* Paulian, 1933) belongs to a species-group in which several species that have orifice. In that species, Pluot-Sigwalt [65] found a high concentration of type G canicules in the region of ventrite I, where the orifice is found. The glandular, sexual and reproductive function of that orifice in *Deltohyboma* remains unknown.

Another relatively common character found in the males is the presence of one or two endophallites in the medial area of endophallus. Only one species-group (*irroratum*) and very few species of *barbipes* species-group do not possess endophallites in that area of endophallus. In the species-groups with two endophallites, it appears that the anterior margin of the clypeus, between clypeal teeth, is concave and slightly expanded posteriorly into a non-triangular shape and the metafemur bears a single margin. Some exceptions can be found (e.g. few species of *plebejum* species-group) where only one medial endophallite is found, or in the *femorale* species-group, which has two endophallites however, the anterior margin of the clypeus is expanded into a triangular shape and the metafemur bears two margins. Otherwise, in all species-groups that have one medial endophallite (having or not the same shape of anterior margin of the clypeus as well as one or two margins in the metafemur) the endophallite is always the one on the right side.

As noted by Cupello *et al*. [66] in Deltochilini genera as well as in other so-called “roller” genera, which lack cephalic or pronotal horns, both strong sexual dimorphism as well as allometric variation in some male structures are uncommon. Several species of *Deltohyboma* present sexually dimorphic allometry, expressed mainly on insertion of the metatibial spur and in tubercles on the posterior edge of the meso- and metafemur. Whether those characteristics have an effect on fitness, perhaps via female stimulation and/or the capacity to make and roll a ball, especially in those species with an elongated insertion of the metatibial spur, remains unknown.

As described in the “Secondary sexual dimorphism” section, there is considerable variation within *Deltohyboma* and, even more interesting, is the fact that quite commonly, species with the same or similar sexual dimorphism have the same type of aedeagus and endophallus, i.e. the same 1) number of medial endophallites, 2) shape of the endophallites and 3) bear or lack raspules. However, in some species with the same sexual dimorphism, e.g. metafemur with setae or with basal steep tapering, the aedeagus is different as well as the endophallus.

Here, we propose 19 species-groups, with a single species left as *incertae sedis*. The species-groups are based on character set (Table 2) described for first time for *Deltohyboma* regarding: the anterior margin of the clypeus (between clypeal teeth); the internal margin of hypomera; the ventral face of the protibia; the posterior margin of the metafemur; secondary sexual dimorphism; and the aedeagus and the endophallus.

**Table 2:** Diagnostic characters for the 19 species-groups here proposed within the subgenus *Deltohyboma* Lane, 1946.

Species-group	Distance between clypeal teeth	Shape of the anterior margin of the clypeus (between clypeal teeth)	Internal margin of hypomera	Ventral surface of the protibia	Posterior edge of metafemur	Male metafemur	Paramera in lateral view	Medial area of endophallus
*aequinoctiale*	At least twice basal width of a tooth	Concave and slightly expanded posteriorly (Fig 5B and 5C)	Not enlarged towards anterior angle (Fig 7A)	With tubercles and/or carina (Fig 9E–9I)	With one margin, ventral surface forming a decline of approximately 45° (Fig 10E and 10F)	Variable	Variable (Fig 13A–13C)	With two endophallites (Fig 16A and 16B)
*aspericolle*	At least twice basal width of a tooth	Concave and expanded posteriorly into triangular shape (Fig 5D)	Strongly enlarged towards anterior angle (Fig 7C)	Without carina or tubercles (Fig 9A–9C and 9J)	With two margins (Fig 10H and 10I)	With basal steep tapering on posterior-ventral margin (Fig 19B arrow, 19C)	Subtriangular with dorsal and ventral edges straight (Fig 13E) or with dorsal edge strongly tapered on apical third followed by broad expansion (Fig 13D).	With one endophallite
*barbipes*	Less than a basal width of a tooth	Flat or slightly concave and expanded posteriorly into triangular shape (Fig 5E and 5F)	Enlarged towards anterior angle (Fig 7B)	Without carina or tubercles (Fig 9A–9C and 9J)	With two margins (Fig 10H and 10I)	Variable	Subtriangular or slender in lateral view (Fig 13F–13H)	With one endophallite (Fig 16C), rarely without endophallites (Fig 16D)
*bidentatum*	Two or 2.5 times basal width of a tooth	Concave and regular, not expanded posteriorly (Fig 5A)	Not enlarged towards anterior angle (Fig 7A)	With tubercles or carina (Fig 9E–9I)	With one margin, ventral surface forming a decline of approximately 45° (Fig 10E and 10F)	More curved than female	Subtriangular,with dorsal and ventral edges straight (Fig 13I)	With two endophallites (Fig 16E)
*femorale*	At least twice basal width of a tooth	Concave and expanded posteriorly into triangular shape (Fig 5D)	Strongly enlarged towards anterior angle (Fig 7C)	Without carina or tubercles (Fig 9A–9C and 9J)	With two margins (Fig 10H and 10I)	With a broad medial serratulate expansion on posterior-ventral margin (Fig 19G)	Subtriangular with dorsal and ventral edges straight (Fig 13J). Apex of paramera with an apical-lateral cleft variable in size (Fig 13J)	With two endophallites (Fig 16F)
*genieri*	At least twice basal width of a tooth	Concave and expanded posteriorly into triangular shape (Fig 5D)	Not enlarged towards anterior angle (Fig 7A)	Without carina or tubercles (Fig 9A–9C and 9J)	With two margins (Fig 10H–10I)	With setae on posterior margin	Subtriangular and with an apical-dorsal sulcus (Fig 14A)	With one endophallite (Fig 16G)
*gilli*	Approximately 1.5 times basal width of a tooth	Concave and expanded posteriorly into triangular shape (Fig 5D)	Strongly enlarged towards anterior angle (Fig 7C)	With a weak carina (Fig 9D)	With two margins (Fig 10H–10I)	With steep tapering and expansion before that steep tapering on posterior-ventral margin (Fig 19I)	Subtriangular, with dorsal and ventral edges straight (Fig 14B)	With one endophallite (Fig 16H)
*granulatum*	Approximately 1.5 times basal width of a tooth	Concave and expanded posteriorly into triangular shape (Fig 5D)	Enlarged towards anterior angle (Fig 7B)	Without carina or tubercles (Fig 9A–9C and 9J)	With one margin, ventral surface continuous to the dorsal margin (Fig 10B and 10C)	With a weak carina on basal third on posterior edge (Fig 19J and 19J’, arrow)	Subtriangular, with dorsal and ventral edges straight (Fig 14C)	With one endophallite
*guyanense*	Twice basal width of a tooth	Concave, regular, slightly expanded or expanded posteriorly (Fig 5A–5C)	Not enlarged towards anterior angle (Fig 7A)	With a weak carina (Fig 9D)	With two margins (Fig 10H and 10I)	With a steep tapering almost medially, forming a broadly dentiform structure on posterior edge (Fig 19K)	Subrectangular in lateral view (Fig 14D). Parameres broadened toward apex in dorsal view (Fig 14D’)	With one endophallite (Fig 16I)
*irroratum*	At least twice basal width of a tooth	Concave and slightly expanded posteriorly (Fig 5B and 5C)	Enlarged towards anterior angle (Fig 7B)	With tubercles or carina (Fig 9E–9I)	With one margin, ventral surface forming a decline of approximately 45° (Fig 10E and 10F)	More curved than female	Slightly slender (Fig 14E)	Without endophallites (Fig 16J)
*komareki*	At least twice basal width of a tooth	Concave and expanded posteriorly into triangular shape (Fig 5D)	Enlarged towards anterior angle (Fig 7B)	With a weak carina (Fig 9D)	With one margin, ventral surface forming a decline of approximately 45° (Fig 10E and 10F)	With a broad basal sub-quadrate expansion on posterior edge (Fig 19L)	Subtriangular, with dorsal and ventral edges straight (Fig 14F)	With one endophallite (Fig 16K)
*lindemannae*	At least twice basal width of a tooth	Concave and expanded posteriorly into triangular shape (Fig 5D)	Strongly enlarged towards anterior angle (Fig 7C)	Without carina or tubercles (Fig 9A–9C and 9J)	With two margins (Fig 10H and 10I)	Variable	Variable (Fig 14G–14I)	With one (Fig 17A) or two endophallites (Fig 16L)
*morbillosum*	At least twice basal width of a tooth	Concave and slightly expanded posteriorly (Fig 5B and 5C)	Enlarged towards anterior angle (Fig 7B)	With carina (Fig 9E and 9H)	With one margin, ventral surface forming a decline of approximately 45° (Fig 10E and 10F)	More curved than female	Subtriangular, with dorsal and ventral edges straight (Fig 14J)	With two endophallites (Fig 17B)
*parile*	At least twice basal width of a tooth	Concave and slightly expanded posteriorly (Fig 5B and 5C)	Not enlarged towards anterior angle (Fig 7A)	With tubercles and/or carina (Fig 9E–9I)	With one margin, ventral surface forming a decline of approximately 45° (Fig 10E and 10F)	Slightly more curved than female and in few species with small expansion	Variable (Fig 15A–15C)	With three endophallites (Fig 17C), two endophallites and one raspule (Fig 17D and 17E) or one endophallite and one raspule.
*plebejum*	Twice basal width of a tooth	Concave and slightly expanded posteriorly (Fig 5B and 5C)	Not enlarged towards anterior angle (Fig 7A)	With tubercles or carina (Fig 9E–9I)	With one margin, ventral surface forming a decline of approximately 45° (Fig 10E and 10F)	More curved than female	Apex with a large or small ventral denticle (Fig 15D and 15E)	With one (Fig 17G) or two endophallites (Fig 17F)
*septemstriatum*	Twice basal width of a tooth	Concave and regular, not expanded posteriorly (Fig 5A)	Strongly enlarged towards anterior angle (Fig 7C)	Without carina or tubercles (Fig 9A–9C and 9J)	With two margins (Fig 10H and 10I)	With a broad medial expansion on posterior-ventral margin (Fig 20C)	Subtriangular, with dorsal and ventral edges straight (Fig 15F)	With one endophallite (Fig 17H)
*sextuberculatum*	At least twice basal width of a tooth	Concave and slightly expanded posteriorly (Fig 5B and 5C)	Enlarged towards anterior angle (Fig 7B)	With tubercles or carina (Fig 9E–9I)	With one margin, ventral surface forming a decline of approximately 45° (Fig 10E and 10F)	With a medial denticle on posterior edge (Fig 20D)	Subtriangular, with dorsal and ventral edges straight (Fig 15G)	With two endophallites (Fig 17I)
*submetallicum*	2.5 times basal width of a tooth	Concave and expanded posteriorly into triangular shape (Fig 5D)	Strongly enlarged towards anterior angle (Fig 7C)	Without carina or tubercles (Fig 9A–9C and 9J)	With one margin, ventral surface continuous to the dorsal margin (Fig 10B and 10C)	With a denticle on basal third of posterior margin (Fig 20E and 20E’, arrow)	Subtriangular, with dorsal and ventral edges straight (Fig 15H)	With one endophallite (Fig 17J)
*susanae*	Two or 2.5 times basal width of a tooth	Concave and expanded posteriorly into triangular shape (Fig 5D)	Strongly enlarged towards anterior angle (Fig 7C)	With carina (Fig 9E and 9H)	With one margin, ventral surface forming a decline of approximately 45° (Fig 10E and 10F)	Not modified	Subtriangular, with dorsal and ventral edges straight (Fig 15I)	With one endophallite (Fig 17K)

In alphabetic order. (For correct identification the use of the identification key to males for the species-groups is recommended, confirmed using the “Description” section).

The dorsal and/or ventral sclerotised paired structures (Figs 13A’, 13B’, 13C’, 15A–15B’, arrows) found between the paramera in some species-groups are described here for the first time for *Deltohyboma*. These structures are easily visible in the hydrated aedeagus; in a dry aedeagus, the same structures may be obscured by the parameres or have the same colour as the membrane. These ventral structures appear to be similar to the “quitinous lobule” described and illustrated by Medina *et al*. [44] for *Eudinopus dytiscoides* (Schreibers, 1802), *Malagoniella astyanax columbica* Harold, 1867, *M*. *astyanax punctatostriata* (Blanchard, 1845), *M*. *puncticollis* (Blanchard, 1845) and *Megathoposoma candezei* Harold, 1873. Nunes & Vaz-de-Mello [67] described a structure between the paramera in ventral view for *Dichotomius* (*Cephagonus*) Luederwaldt, 1929, which they named subgenital plate; that plate seems to be the same structure described by Medina *et al*. [44] as “quitinous small plate” that they observed in *Copris dracunculus* Ferreira, 1959, *C*. *incertus* Say, 1835, *C*. *mesacanthus* Harold, 1878, *Dichotomius bos* (Blanchard, 1846) and *Ontherus sanctaemartae* Génier 1996. It appears that Medina *et al*. [44] considered the “quitinous lobule” to be a different structure to the “quitinous small plate”. Even though the “quitinous lobule” appears to be similar to the structures found in *Deltohyboma*, we decided not to use the same terminology for the structure nor to present it as a new (named) structure. This is because we have not studied the aedeagi of the other genera and are, therefore, not able to hypothesise on potential homologies or state whether it is the same structure, or not. This way, we will not add to a potential list of structure synonymies for the aedeagus within Scarabaeinae.

There is the possibility that, in a future phylogenetic analysis, several of these species-groups proposed here could be recovered together. The phylogenetic relationships of *Deltochilum* within Deltochilini as well as that of its eight subgenera remain almost completely unresolved. *Deltochilum*, with 110 species and an estimated diversity of almost 300 species, is exceptional in its morphological variability. Adding to the fact that almost half of the diversity remains unknown, i.e. undescribed species, a phylogenetic analysis could prove notably difficult to produce.

### Identification key to males and females for the species-groups of the subgenus *Deltohyboma*

1. Medial emargination of clypeus narrowly U-shaped. Clypeal teeth separated by less than a basal tooth width (Fig 5E and 5F). Anterior margin of the clypeus, between clypeal teeth, flat (Fig 5E) or slightly concave (Fig 5F) and expanded posteriorly into triangular shape (Fig 5E and 5F)… ***barbipes* species-group**

1’. Medial emargination of clypeus broadly U-shaped or truncated. Clypeal teeth separated by at least a basal tooth width (Fig 5A–5D). Anterior margin of the clypeus, between clypeal teeth, concave (Fig 5A–5D) and either regular (Fig 5A), slightly expanded (Fig 5B and 5C) or expanded posteriorly into triangular shape (Fig 5D)… 2

2. Anterior margin of the clypeus, between clypeal teeth, expanded posteriorly into triangular shape (Fig 5D) … 3

2’. Anterior margin of the clypeus, between clypeal teeth, regular or slightly expanded, not triangular in shape (Fig 5A–5C) … 12

3. Posterior edge of metafemur with one margin, the posterior-dorsal (Fig 10B, 10C, 10E and 10F); ventral surface of metafemur continuous to the dorsal margin (Fig 10B and 10C) or forming a 45° decline on posterior-ventral edge (Fig 10E and 10F) … 4

3’. Posterior edge of metafemur with two margins; one ventral forming a decline of 45° and the other one dorsal (Fig 10H and 10I) … 7

4. Ventral surface of metafemur continuous to the posterior-dorsal margin (Fig 10B and 10C) … 5

4’. Ventral surface of metafemur forming a 45° decline on posterior-ventral edge (Fig 10E and 10F) … 6

5. Punctures on metaventral disc conspicuous at 8x magnification. Metaventral disc punctures almost half the size of punctures on anterior-lateral area of metaventral process. Internal margin of hypomera strongly enlarged towards anterior angle (Fig 7C, arrows). Striae I-VII conspicuous and narrow, width of each stria approximately 1/28th of the width of each interstria (Fig 4J). Tubercles on interstriae at elytral apex with any of the following variations: 1) III, V-VII; 2) V-VII or 3) VI-VII, if III present small and poor developed and smaller than tubercle on V. Male metafemur with denticle on basal third of posterior-ventral margin (Fig 20E, and 20E’, arrow) … ***submetallicum* species-group**

5’. Punctures on metaventral disc inconspicuous at 8x magnification. Metaventral disc punctures several times smaller than punctures on anterior-lateral area of metaventral process. Internal margin of hypomera slightly enlarged towards anterior angle (Fig 7B, arrows). Striae I-II conspicuous, III-VII narrow and effaced, successively narrower and more effaced (Fig 3J), with VII almost inconspicuous. Tubercles at elytral apex on interstriae III, V-VII, with III large and well developed, of same size or larger than tubercle on V. Male metafemur with weak carina on basal third of posterior edge (Fig 19J and 19J’, arrow) … ***granulatum* species-group**

6. Medial-lateral angle of pronotum rounded (Fig 4K). Ventral face of protibia with carina (as Fig 9E). Metaventral disc with punctures half the size of punctures on anterior-lateral area of metaventral process. Small species, length 7.4–9.4mm and humeral width 4.6–6.2mm. Male mesofemur and metafemur unmodified … ***susanae* species-group**

6’ Medial-lateral angle of pronotum projected. Ventral face of protibia with a weak carina (as Fig 9D). Metaventral disc with punctures slightly smaller than punctures on anterior-lateral area of metaventral process. Medium-sized species, length 10.2-12mm, and humeral width 7.5-8mm. Male metafemur modified: posterior edge with broad basal, sub-quadrate expansion (Fig 19I), that is bifurcated in some specimens. Male mesofemur slightly sinuate basally … ***komareki* species-group**

7. Ventral surface of the protibia with weak carina (as Fig 9D) … ***gilli* species-group**

7’. Ventral surface of the protibia without carina (as Fig 9A–9C and 9J) … 8

8. Eyes small to medium-sized, inter-ocular distance over nine times width of one eye … 9

8’. Eyes large, inter-ocular distance under nine times width of one eye … 10

**Fig 22:**
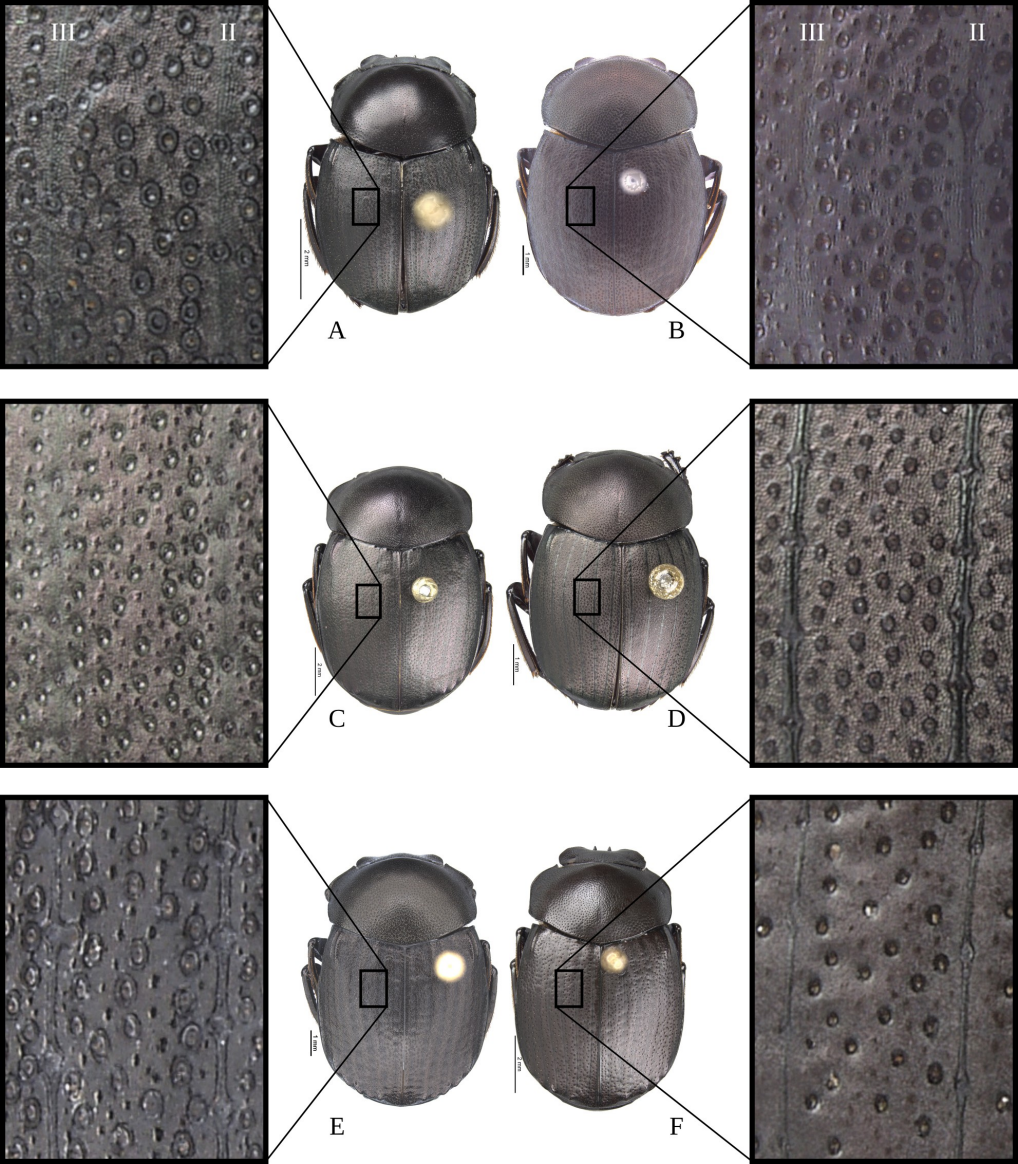
Elytral microsculpture of *Deltohyboma*. Roman numerals correspond to striae. (A) *femorale* species-group. (B) *aspericolle* species-group. (C) *guyanense* species-group. (D) *septemstriatum* species-group. (E) *sextuberculatum* species-group. (F) *plebejum* species-group.

9. Striae I-VII inconspicuous (Figs 3G and 22A). Male metafemur with broad medial serratulate expansion on posterior-ventral margin (Fig 19G). Paramera with apical-lateral cleft (Fig 13J) … ***femorale* species-group**

9’. Striae I-VII conspicuous (Figs 3C and 12A), or I-II conspicuous III-VII successively narrower and more inconspicuous (Figs 3B and 22B), with VII almost inconspicuous. Male metafemur with basal steep tapering on posterior-ventral edge (Fig 19B, arrow). Paramera without apical-lateral cleft (Fig 13D and 13E) … ***aspericolle* species-group**

10. Internal margin of hypomera not enlarged towards anterior angle (Fig 7A, arrows). Paramera with apical-dorsal sulcus (Fig 14A and 14A’). Male metafemur with posterior setae … ***genieri* species-group**

10’. Internal margin of hypomera strongly enlarged towards anterior angle (Fig 7C, arrows). Paramera without apical-dorsal sulcus (Figs 14G–14I and 15J). Male metafemur with or without posterior setae … 11

11. Pronotum with shiny points mixed with punctures (see Fig 6). Male mesofemur unmodified. Male metafemur with or without posterior setae (Fig 20A and 20B), if without setae first ventrite with basal spine and/or expansion of first ventrite almost reaching distal margin of ventrite V. Female with distal margin of fifth ventrite regular or only with a slightly sinuosity. Aedeagus with paramera slightly slender or slender, if slightly slender, paramera almost half the length of phallobase (Fig 14G–14I). Medial area of endophallus with one or two endophallites, if with one not “T”-shaped (Figs 16L and 17A)… ***lindemannae* species-group**

11’. Pronotum without shiny points mixed with punctures (see Fig 6). Male posterior edge of meso- and metafemur with short basal expansion, most acute on mesofemur. Male expansion of first ventrite reaching distal margin of ventrite IV. Female with distal margin of fifth ventrite acutely expanded medially. Aedeagus with paramera lightly smaller than phallobase (Fig 15J). Medial area of endophallus with one “T”-shaped endophallite (Fig 17L) … ***Deltochilum inesae* sp. nov. (*incertae sedis*)**

12. Posterior edge of metafemur with two margins; one ventral, forming a 45° decline and the other one dorsal (Fig 10H and 10I). Ventral surface of the protibia with (Fig 9D) or without weak carina (Fig 9A–9C and 9J), never with tubercles … 13

12’. Posterior edge of metafemur with one margin, the posterior-dorsal; ventral surface of metafemur forming a 45° decline on ventral edge (Fig 10E and 10F). Ventral surface of the protibia with carina or tubercles (Fig 9E–9I) … 14

13. Striae inconspicuous (Figs 3K and 22C). Medium-sized species, length 10.2–13.3mm. Ventral face of the protibia with weak carina (Fig 9D). Posterior edge of male metafemur with strong steep tapering almost medially, forming a broadly dentiform structure (Fig 19K) … ***guyanense* species-group**

13’. Striae conspicuous (Figs 4H and 22D). Small species, length 6.1–7.3mm. Ventral face of the protibia without carina (Fig 9A–9C and 9J). Posterior edge of male metafemur with broad medial expansion (Fig 20C) … ***septemstriatum* species-group**

**Fig 23:**
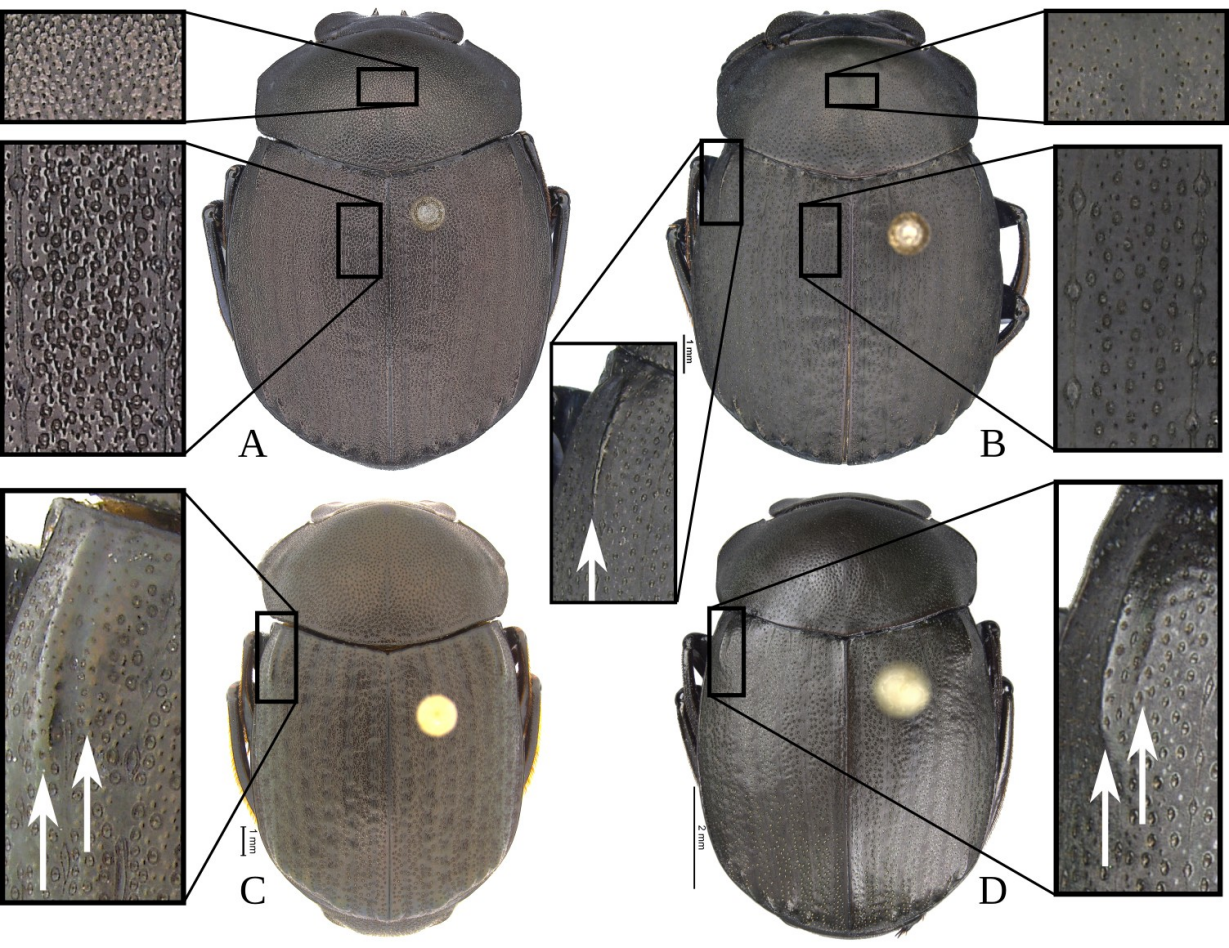
Elytral and pronotal microsculpture (A-B), showing shiny points mixed with the punctures, and detail of elytral humeral region (B-D) of *Deltohyboma*. Arrows correspond to tubercle on interstria VII (B) or tubercles on interstriae VI-VII (C-D). (A) *bidentatum* species-group. (B) *morbillosum* species-group. (C) *irroratum* species-group. (D) *parile* species-group.

14. Elytra with basal tubercle on interstria VII (Figs 3F, 4C and 23A and 23B, arrow) … 15

14’ Elytra with basal tubercles on interstriae VI-VII (Figs 3A, 3L, 4D–4G, 23C and 23D, arrows) … 16

15. Pronotal disc (Fig 23A) and interstriae (Fig 23A) with large, elevated shiny points mixed with punctures. Shiny points on pronotal disc almost contiguous, separated by less than one diameter of a shiny point. Male ventrite I expanded posteriorly reaching or surpassing middle of ventrite V. Well-developed males with elongate insertion of metatibial spur (Fig 11B) … ***bidentatum* species-group**

15’. Pronotal disc (Fig 23B) and interstriae (Fig 23B) with small, not elevated shiny points mixed with punctures. Shiny points on pronotal disc separated by one or more than one diameter of a shiny point. Male ventrite I expanded posteriorly, expansion almost reaching distal margin of ventrite II to reaching distal margin of ventrite III. Males without elongate insertion of metatibial spur (Fig 11A) … ***morbillosum* species-group**

16. Striae broad, width of third stria, in species with the narrowest striae, 1/15th of the distance between striae II and III (Figs 4I and 22E). Metaventrite with a small, weak posterior excavation occupying basal fourth (as Fig 8B) … ***sextuberculatum* species-group**

16’. Striae inconspicuous (Figs 3A, 4D and 4F) or conspicuous (Figs 3L, 4E and 4G); if conspicuous, narrow or broad; if narrow, width of third stria, in species with the broadest striae, 1/20th of the distance between striae II and III (Figs 4G and 22F). If broad, width of third stria on disc between 1/12th and 1/15th of the distance between striae II and III, then metaventrite with strong, large posterior excavation, excavation surpassing or almost reaching the middle of metaventral length (Fig 8C) … 17

17. Metatarsomeres II and III not elongate, each almost as long as broad (Fig 11A) … ***irroratum* species-group**

17’. Metatarsomeres II and III elongate, each longer than broad (Fig 11B) … 18

**Fig 24:**
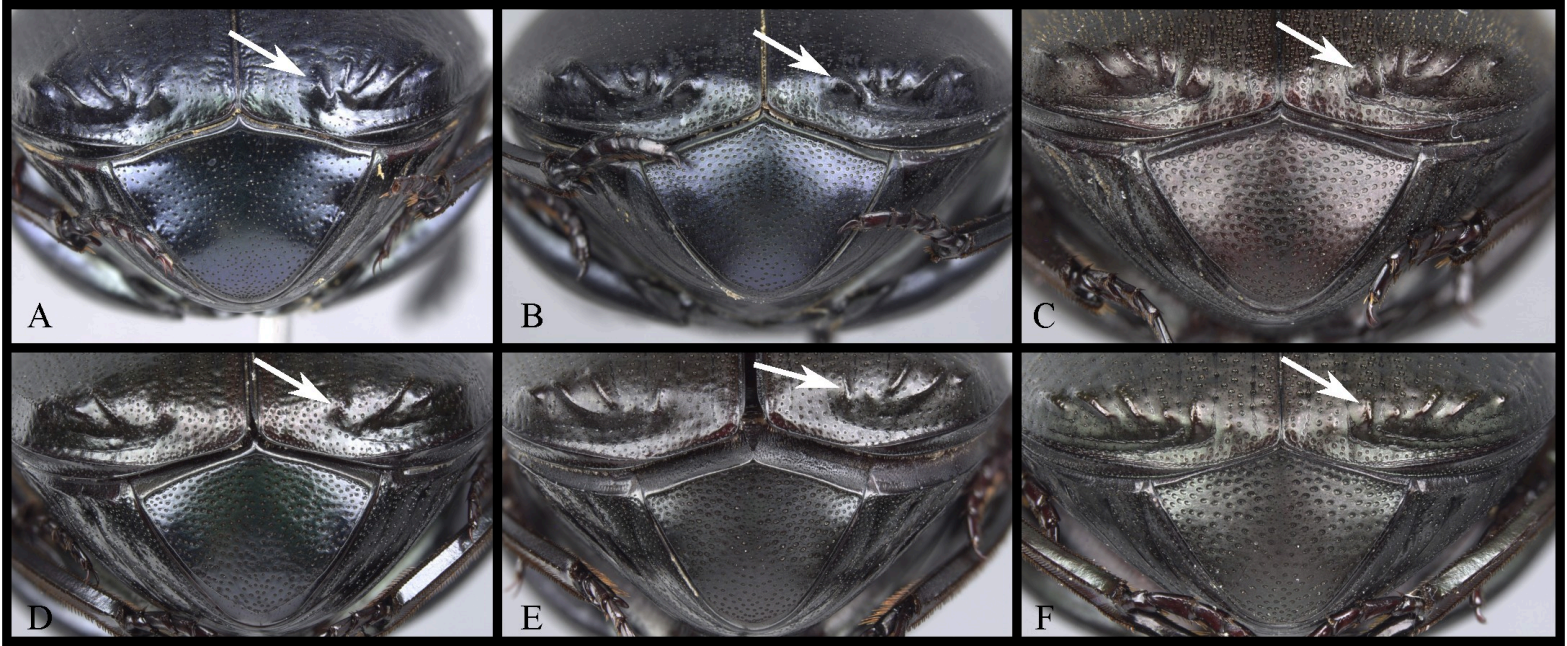
Caudal view of *aequinoctiale*, *parile* and *plebejum* species-groups. Arrow showing the tubercle on interstriae III. (A) *aequinoctiale* species-group. (B-C) *parile* species-group. (D-F) *plebejum* species-group.

18. Tubercles at elytral apex on interstriae with any of the following variations: 1) III, V-VII with all tubercles well developed; 2) III-VII with all tubercles well developed or with IV poorly developed; 3) II-VII with II only slightly elevated, but on all those variations tubercle on III elongate (Fig 24D–24F, arrow). Male with first ventrite expanded posteriorly (Fig 21E). Apex of paramera without setae (Fig 15D and 15E) … ***plebejum* species-groups**

18’. Tubercles at elytral apex on interstriae III-VII with all tubercles well developed or with IV poorly developed, however tubercle on III approximately triangular in shape or transverse (Fig 24A–24C), never elongate. Male with first ventrite expanded (Fig 21F) or not (Fig 21D) posteriorly. Apex of paramera with setae (Figs 13A–13C and 15A–15C) … 19

19. Male with first ventrite expanded posteriorly (Fig 21F). Aedeagus with dorsal (Fig 13C’, arrow) and/or ventral (Fig 13A’ and 13B’, arrows) sclerotised paired structures, separated from paramera. Medial area of endophallus with two endophallites, both subequal in shape (sub-rectangular) and right larger than left (Fig 16A and 16B) … ***aequinoctiale* species-group**

19’. Male with first ventrite not expanded (Fig 21D) or expanded posteriorly. If not expanded aedeagus with dorsal sclerotised paired structures fused with the paramera (Fig 15A–15B’, arrows). If first ventrite expanded, aedeagus without dorsal sclerotised paired structures (Fig 15C), however, both types of aedeagi with medial area of endophallus with one, two or three endophallites (differing in shape), and right endophallite triangular or sub-triangular in shape (Fig 17C–17E) … ***parile* species-group**

### Identification key to males for the species-groups of the subgenus *Deltohyboma*

1. Medial emargination of clypeus narrowly U-shaped. Clypeal teeth separated by less than a basal tooth width (Fig 5E and 5F). Anterior margin of the clypeus, between clypeal teeth, flat (Fig 5E) or slightly concave (Fig 5F) and expanded posteriorly into triangular shape (Fig 5E and 5F)… ***barbipes* species-group**

1’. Medial emargination of clypeus broadly U-shaped or truncated. Clypeal teeth separated by at least a basal tooth width (Fig 5A–5D). Anterior margin of the clypeus, between clypeal teeth, concave (Fig 5A–5D) and either regular (Fig 5A), slightly expanded (Fig 5B and 5C) or expanded posteriorly into triangular shape (Fig 5D)… 2

2. First ventrite medially not expanded posteriorly (Fig 21D) or slightly expanded reaching at most ventrite III … 3

2’. First ventrite expanded posteriorly reaching at least ventrite IV (Fig 21B, 21C and 21E–21I) … 5

3. Elytra with basal tubercle on interstria VII (Figs 4C, 23A and 23B, arrow) … ***morbillosum* species-group**

3’. Elytra with basal tubercles on interstriae VI-VII (Figs 3L, 4D, 4E, 23C and 23D, arrows) … 4

4. First ventrite medially slightly expanded posteriorly reaching distal margin of second or third ventrite. Metaventrite with strong large excavation posteriorly, surpassing middle of metaventral length (Fig 8C). Paramera without dorsal paired sclerotised structures (Fig 14E’). Medial area of endophallus without endophallites (Fig 16J) … ***irroratum* species-group (part)**

4’. First ventrite not expanded posteriorly. Metaventrite with weak, small excavation posteriorly, not reaching middle of metaventral length (Fig 8B). Paramera with dorsal paired sclerotised structures attached to the internal edge of paramera (Fig 15A–15B’, arrows). Medial area of endophallus with two or three endophallites (Fig 17C and 17D) … ***parile* species-group (part)**

5. Metafemur modified, posterior edge with either weak carina, denticle, setae, steep tapering or expansion (Figs 19B–19L and 20) … 6

5’. Metafemur subequal or only more curved than that of females … 19

6. Posterior edge of metafemur with steep tapering medially or basally (Fig 19B, 19C, 19I and 19K) … 7

6’. Posterior edge of metafemur without steep tapering, but with either weak carina (Fig 19J), denticle (Fig 20E), setae (Figs 19H, 20A and 20B) or expansion (Figs 19G and 20C) … 9

7. Posterior edge of metafemur with strong steep tapering almost medially, forming a broadly dentiform structure (Fig 19K). Aedeagus with paramera broadened toward apex and apex of paramera truncate in dorsal view (Fig 14D and 14D’). Paramera flattened in ventral view (Fig 14D) … ***guyanense* species-group**

7.’ Posterior edge of metafemur with steep tapering on basal third, with (Fig 19I) or without (Fig 19B and 19C) expansion before that steep tapering. Aedeagus with different shape … 8

8. Posterior edge of metafemur expanded before the steep tapering (Fig 19I). Paramera with straight dorsal and ventral edges in lateral view (Fig 14B). Apex of paramera rounded in dorsal view (Fig 14B’). Paramera with short and thin apical-dorsal notch (Fig 14B’). Basal endophallite circular-shape with ring very thin and handle strongly broadened medially … ***gilli* species-group**

8’. Posterior edge of metafemur not expanded before the steep tapering (Fig 19B and 19C). Paramera with straight dorsal and ventral edges in lateral view (Fig 13E) or with dorsal edge with a strong tapering on apical third followed by broad expansion in lateral view (Fig 13D). In both, apex formed of paramera truncated in dorsal view (Fig 13D’ and 13E’). Paramera without apical-dorsal notch (Fig 13D’ and 13E’). Basal endophallite circular-shape with ring very thin and handle broadened … ***aspericolle* species-group**

9. Posterior edge of metafemur with setae … 10

9’. Posterior edge of metafemur without setae … 12

10. Anterior margin of the clypeus, between clypeal teeth, slightly expanded, but not triangular in shape (Fig 5B) … ***aequinoctiale* species-group (part)**

10’. Anterior margin of the clypeus, between clypeal teeth, expanded posteriorly into triangular shape (Fig 5D) … 11

11. Internal margin of hypomera regular, not enlarged towards anterior angle (Fig 7A, arrows). Paramera with apical-dorsal sulcus (Fig 14A and 14A’) … ***genieri* species-group**

11’. Internal margin of hypomera strongly enlarged towards anterior angle (Fig 7C, arrows). Paramera without apical-dorsal sulcus (Fig 14G–14I’). Paramera commonly slender in lateral view… ***lindemannae* species-group (part)**

12. Posterior edge of metafemur with short or broad expansion (Figs 19G, 19L, 20C and 20F) … 13

12’. Posterior edge of metafemur with carina or denticle (Figs 19J, 20D and 20E) … 16

13. Posterior edge of meso- and metafemur (Fig 20F) with short basal expansion, more acute on mesofemur. Aedeagus with paramera lightly smaller than phallobase (Fig 15J). Medial area of endophallus with one “T”-shaped endophallite (Fig 17L) … ***Deltochilum inesae* sp. nov. (*incertae sedis*)**

13’. Posterior edge of metafemur with broad expansion (Figs 19G, 19L and 20C); mesofemur unmodified or slightly sinuate basally. Aedeagus with paramera almost half as long as phallobase (Figs 13J, 14F and 15F). Medial area of endophallus with one (Figs 16K and 17H) or two (Fig 16F) endophallites; if with only one not “T”-shaped (Figs 16K and 17H) … 14

14. Posterior edge of metafemur with broad basal sub-quadrate expansion (Fig 19L), that can be bifurcated in some specimens. Mesofemur slightly sinuate basally. Apex of paramera without apical-lateral cleft (Fig 14F’). Internal margins of paramera broadened on basal half in dorsal view and external margins of paramera broadened toward apex in dorsal view (Fig 14F’) … ***komareki* species-group**

14’. Posterior edge of metafemur with broad medial expansion, serratulate (Fig 19G) or not (Fig 20C). Mesofemur not modified. Apex of paramera with (Fig 13J) or without apical-lateral cleft (Fig 15F). If without apical-lateral cleft internal and external margins almost straight in dorsal view (Fig 15F’) … 15

15. Striae inconspicuous (Figs 3G and 22A). Posterior edge of metafemur with broad medial serratulate expansion (Fig 19G). Apex of paramera with apical-lateral cleft (Fig 13J) … ***femorale* species-group**

15’. Striae conspicuous (Figs 4H and 22D). Posterior edge of metafemur with broad medial expansion, not serratulate (Fig 20C). Apex of paramera without apical-lateral cleft (Fig 15F) … ***septemstriatum* species-group**

16. Posterior edge of metafemur with weak basal carina (Fig 19J and 19J’). Medial area of endophallus with one comma-shaped endophallite. Basal circular-shaped endophallite with the ring thin and well sclerotised … ***granulatum* species-group**

16’. Posterior edge of metafemur with denticle (Fig 20D and 20E). Medial area of endophallus with one (Fig 17J) or two endophallites (Figs 16A, 16B and 17I). If with one endophallite, straight (Fig 17J) not comma-shaped and basal circular-shaped endophallite with very small, thin and poorly sclerotised ring … 17

17. Metatrochanter with long, dense setae or with a denticulate process. Aedeagus with paramera with dorsal (Fig 13C’, arrow) and/or ventral (Fig 13A’ and 13B’, arrows) strongly sclerotised paired structures … ***aequinoctiale* species-group (part)**

17’. Metatrochanter unmodified. Aedeagus with paramera without dorsal or ventral strongly sclerotised paired structures (Fig 15G’ and 15H’) …18

18. Posterior edge of metafemur with basal denticle (Fig 20E). Anterior margin of the clypeus, between clypeal teeth, expanded posteriorly into triangular shape (Fig 5D). Medial area of endophallus with one endophallite (Fig 17J). Basal circular-shaped endophallite with very small, thin and poorly sclerotised ring (Fig 17J’) … ***submetallicum* species-group**

18’ Posterior edge of metafemur with medial denticle (Fig 20D). Anterior margin of the clypeus, between clypeal teeth, slightly expanded but not triangular in shape (Fig 5B and 5C). Medial area of endophallus with two endophallites (Fig 17I). Basal circular-shaped endophallite with thin and well sclerotised ring … ***sextuberculatum* species-group**

19. Elytra with basal tubercle on interstria VII (Figs 3F, 23A and 23B, arrow) … ***bidentatum* species-group**

19’ Elytra with basal tubercles on interstriae VI-VII (Figs 3A, 3L, 4B, 4D-4G, 4K, 23C and 23D, arrows) … 20

20. Anterior margin of the clypeus, between clypeal teeth, expanded posteriorly into triangular shape (Fig 5D) … 21

20’. Anterior margin of the clypeus, between clypeal teeth, slightly expanded, but not triangular in shape (Fig 5B and 5C) … 22

21. Width of expansion of first ventrite, on third ventrite, three to four times the distance between clypeal teeth. Aedeagus with subtriangular paramera, dorsal and ventral edges straight in lateral view (Fig 15I). Medial area of endophallus with one comma-shaped endophallite (Fig 17K). Sub-medial area of endophallus without raspules or large scales … ***susanae* species-group**

21’. Width of expansion of first ventrite, on third ventrite, subequal to the distance between clypeal teeth. Aedeagus with lightly slender paramera in lateral view (Fig 14I). Medial area of endophallus with two endophallites. Sub-medial area of endophallus with raspules or large scales … ***lindemannae* species-group (part)**

22. Meso and metatrochanters modified, with dense setae or dentiform process … 23

22’. Meso and metatrochanters unmodified … 24

23. Mesofemur modified with posterior dispersed setae. Aedeagus with paramera lacking dorsal or ventral strongly sclerotised paired structures (Fig 14E’). Medial area of endophallus lacking endophallites (Fig 16J) … ***irroratum* species-group (part)**

23’. Mesofemur modified or not, if modified with small or large dentiform process. Aedeagus with paramera bearing dorsal (Fig 13C’, arrow) and/or ventral (Fig 13A’ and 13B’, arrows) strongly sclerotised paired structures. Medial area of endophallus with two endophallites (Fig 16A and 16B) … ***aequinoctiale* species-group (part)**

24. Elytra with triangular apical tubercle on third interstria (Fig 24B and 24C). Apex of paramera with setae (Fig 15C). Paramera with strong basal sinuation in lateral view (Fig 15C, arrow). Medial area of endophallus with one or two endophallites and with large scales (Fig 17E, arrow) … ***parile* species-group (part)**

24’. Elytra with elongate apical tubercle on third interstria (Fig 24D and 24F). Apex of paramera without setae (Fig 15D and 15E). Paramera without strong basal sinuation in lateral view (Fig 15D and 15E). Medial area of endophallus one or two endophallites, but without large scales (Fig 17F and 17G) … ***plebejum* species-group**

### The *aequinoctiale* species-group

#### Diagnosis

For all species known of *Deltohyboma*, the species belonging to the *aequinoctiale* species-group can only be distinguished via the aedeagus. Despite the aedeagi being highly variable in terms of shape, they always bear either dorsal and/or ventral strongly sclerotised paired structures which arise separately from the paramera (Fig 13A’, 133B’ and 13C’, arrows).

#### Description

Medium to large-sized species, length 11-17mm, humeral width 7.5-9mm. Clypeal median emargination broadly U-shaped or almost truncated. Clypeal teeth separated by at least twice basal width of a tooth. Anterior margin of the clypeus, between clypeal teeth, concave, slightly expanded posteriorly, but not triangular in shape (Fig 5B and 5C). Eyes large, inter-ocular distance seven to eight times width of one eye. Internal margin of hypomera not enlarged towards anterior angle (Fig 7A, arrows). Tubercles at elytral apex on interstriae III-VII with all tubercles well developed or with IV poorly developed; tubercle on III approximately triangular in shape (Fig 24A, arrow) or transverse. Interstriae VI-VII with basal tubercles (Fig 3A, see also Fig 23C and 23D, arrows). Striae inconspicuous (Fig 3A) or conspicuous. If conspicuous very narrow, width of the third stria, in species with the broadest striae, 1/40th of the distance between striae II and III. Metaventrite with one or two, weak or strong posterior excavations, if weak occupying metaventral basal third, if strong may occupy basal third or reach middle of the metaventrite length, bearing a small or large tubercule on anterior edge of excavation or on anterior edge of anterior most excavation. Ventral surface of protibia with tubercles and/or carina (Fig 9E–9I). Posterior edge of metafemur with one margin, the dorsal, where the ventral surface of metafemur forming a decline of approximately 45° on posterior edge (Fig 10E and 10F). **Male**. Protibial spur broad and/or foliaceus. Mesofemur with or lacking a tubercle on basal third or on apical third. Apex of mesotibia on ventral-internal margin with a small spatulate expansion (Fig 18C) or small denticle (Fig 18B). Metafemur not modified or with long setae, small denticle or a carina. Internal margin of metatibia modified or not, if modified with tubercles (see Fig 18E and 18G), strong carina (see Fig 18F), or both. Metatibia with spur insertion elongate (Figs 11B and 18E) or not (Figs 11A and 18F) and with spur articulated; if spur insertion elongate, spur reaching second tarsomere or almost as long as tarsus. Ventrite I expanded posteriorly (see Fig 21), expansion reaching distal margin of ventrite IV; width of the expansion of ventrite I, on ventrite III, from three to five times as wide as distance between clypeal teeth. Aedeagus highly variable in shape (Fig 13A–13C) however, between paramera with dorsal (Fig 13C’, arrow) and/or ventral (Fig 13A’ and 13B’, arrows) strongly sclerotised paired structures variable in shape; those structures are separated by paramera. Apex of paramera with sparse setae (Fig 13A–13C). Medial area of endophallus with two endophallites (Fig 16A and 16B), right endophallite sub-rectangular in shape, small or large, left subequal in shape and commonly smaller than right endophallite. Sub-medial area of endophallus with or without one denticle-shaped endophallite (Fig 16B).

#### Composition

Seven described species, five valid: *D*. *aequinoctiale* (Buquet, 1844), *D*. *spinipes* Paulian,1938, *D*. *gigante* Silva & Vaz-de-Mello, 2014, *D*. *speciosissimum* Balthasar, 1939, *D*. *pretiosum* Harold, 1875, *D*. *erodioides* Harold, 1867 (junior synonym of *D*. *aequinoctiale*), *D*. *haroldi* Kirsch, 1885 (junior synonym of *D*. *pretiosum*) and at least 19 undescribed species.

#### Geographic distribution

**Fig 25:**
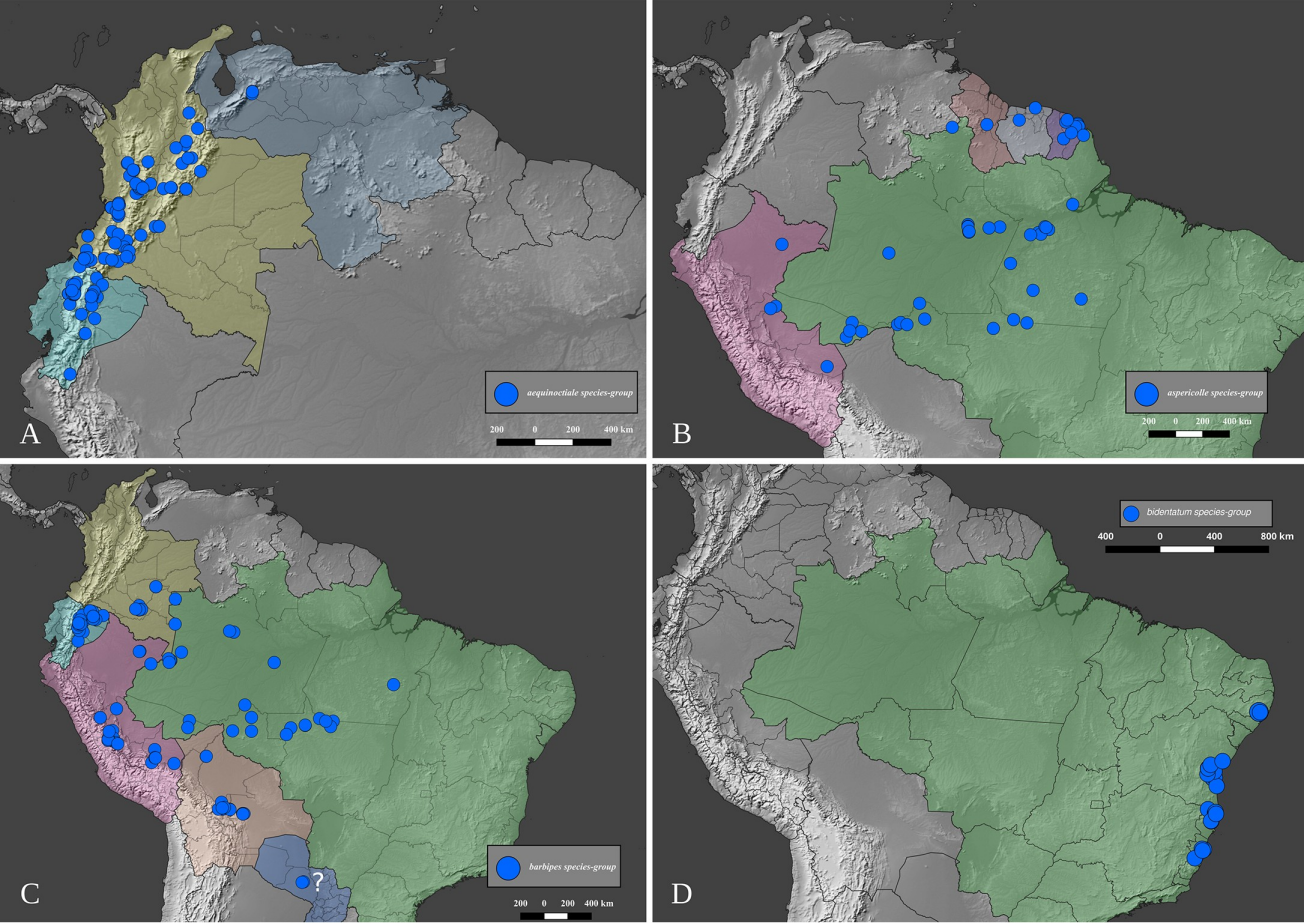
Distribution maps. (A) *aequinoctiale* species-group. (B) *aspericolle* species-group. (C) *barbipes* species-group, “?” showing locality with doubt. (D) *bidentatum* species-group.

The species in this species-group are known to be distributed (Fig 25A), so far, in the following dominions and provinces (in parentheses): Boreal Brazilian (Napo) and Pacific (Sabana, Guajira, Magdalena, Cauca, Chocó-Darién), as well as in the South American transition zone, Paramo province.

This species-group can be found in several localities in sympatry with the following species-groups (Table 3): *barbipes*, *genieri*, *parile* and *plebejum*.

**Table 3:** Sympatry of the *aequinoctiale* species-group with other species-groups by province.

Species-group	Province(s)
*barbipes*	Napo (one locality, Ecuador, Napo, Tena)
*genieri*	Paramo (a few localities)
*parile*	Magdalena, Cauca (several localities)
*plebejum*	Sabana and Paramo (few localities)

#### Remarks

The *aequinoctiale* species-group appears to be closely related to *parile* and *plebejum* species-groups, but can be separated by the elongate tubercle on interstria III (Fig 24D–24F, arrows) at elytral apex in *plebejum*, which is not elongated but triangular in shape (Fig 24B and 24C, arrows) or transverse in *aequinoctiale* species-group. The shape of that tubercle is the same in *aequinoctiale* (Fig 24A, arrow) and *parile* (Fig 24D–24F, arrows) species-groups. However, the most reliably way to separate those species-groups is via the aedeagus. The apex of paramera bear setae in *aequinoctiale* and *parile* species-groups, whereas the setae are absent in *plebejum* species-group.

Commonly, the species in the *aequinoctiale* species-group are larger and more colourful compared to those in the *parile* species-group. In the latter, the species are commonly brown and the colour is almost the same dorsally and ventrally. However, the best way to separate these species-groups is via the males. In the *aequinoctiale* species-group the first ventrite is always expanded posteriorly (Fig 21F), the aedeagus bears strongly sclerotised dorsal and/or ventral paired structures, both separated from paramera (Fig 13A’, 13B’ and 13C’, arrows). Moreover, the medial area of endophallus bears two endophallites (Fig 16A and 16B).

By contrast, in the *parile* species-group, the first ventrite is frequently not expanded posteriorly (Fig 21D) and the aedeagus bears only dorsal paired sclerotised structures which are fused with the paramera (Fig 15A–15B’, arrows). In three undescribed species, the first ventrite is expanded posteriorly and the aedeagus lacks the paired dorsal sclerotised structures. However, despite the variability, the medial area of the endophallus always possesses one of the following three combinations endophallites and raspules: 1. three endophallites (Fig 17C), 2. two endophallites and one raspule (Fig 17D and 17E), 3. one endophallite and one raspule. The right endophallite is always triangular or sub-triangular (Fig 17C–17E).

### The *aspericolle* species-group

#### Diagnosis

This species-group shares the following combination of character states with the *femorale*, *genieri*, *lindemannae* species-groups and *Deltochilum inesae* **sp. nov.** (*incertae sedis*): anterior margin of the clypeus, between clypeal teeth, concave and expanded posteriorly into triangular shape (Fig 5D); posterior edge of metafemur with two margins (Fig 10H and 10I); ventral surface of protibia without carina or tubercles (Fig 9A–9C and 9J). However, males can be easily distinguished from the above mentioned species and the species-groups by male metafemur with basal steep tapering on posterior margin (Fig 19B, arrow and 19C) as well as by the shape of aedeagus (Fig 13D and 13E). It can also be separated from the *femorale* species-group by the inconspicuous striae (Fig 3G), which are conspicuous in *aspericolle* species-group (Fig 3B and 3C) and from *genieri* and *lindemannae* species-groups as well as *Deltochilum inesae* **sp. nov.** (*incertae sedis*) by the size of the eyes. In *aspericolle* species-group the inter-ocular distance is over nine times the width of one eye whereas, for the others, the inter-ocular distance is under nine times the width of one eye.

**Description.** Medium-sized species, length 6.8–11.4mm, humeral width 4.3-7mm. Clypeal median emargination broadly U-shaped. Clypeal teeth separated at least by twice basal width of a tooth. Anterior margin of the clypeus, between clypeal teeth, concave and expanded posteriorly into triangular shape (Fig 5D). Eyes medium-sized, inter-ocular distance ten to 15 times width of one eye. Internal margin of hypomera strongly enlarged towards anterior angle (Fig 7C, arrows). Tubercles at elytral base on interstriae VI-VII (Fig 3B and 3C, see also Fig 23C and 23D, arrows). Tubercles at elytral apex on interstriae with any of following variations: 1) III, V-VII with all tubercles well developed, or 1a) III, V-VII with III poorly developed, or 1b) III, V-VII with III and V poorly developed; 2) V-VII with all tubercles well developed, or 2a) V-VII with V poorly developed; 3) VI-VII all well developed. Striae with following variations: 1) narrow (Fig 3B), width of third stria between 1/20th and 1/45th of distance between stria II and III, or 1a) with striae III-VII subequal in width, or 1b) with striae III-VII consecutively more effaced (Fig 3B) or more discontinuous, in few species with VI or VI-VIII almost inconspicuous; 2) broad (Fig 3C), width of third stria between 1/18th and 1/15th of distance between stria II and III, with striae III-VII subequal in width. Stria VIII with any of following variations: 1) conspicuous apical and laterally, 2) conspicuous apical and laterally but discontinuous either apical or laterally, 3) conspicuous apically only; if conspicuous laterally, not reaching, reaching or surpassing the apex of carina of the ninth interstria. Metaventrite with weak posterior excavation, approximately occupying metaventral basal fourth. Ventral surface of protibia without carina or tubercles (Fig 9A–9C and 9J). Posterior edge of metafemur with two margins (Fig 10H and 10I). **Male**. Protibial spur broad and foliaceus. Mesofemur with slight sinuosity on apical third. Apex of mesotibia on ventral-internal margin with small or large spatulate expansion (Fig 18C, arrow). Metafemur with basal steep tapering on posterior-ventral margin (Fig 19B arrow and 19C). Internal margin of metatibia with small or large tubercles (see Fig 18E, 18G and 18H). Ventrite I expanded posteriorly (see Fig 21); ventrite VI narrow medially. Paramera subtriangular, with dorsal and ventral edges straight in lateral view (Fig 13E) or with dorsal edge strongly tapered on apical third followed by broad expansion (Fig 13D) in lateral view. In both cases, apex of paramera truncated in dorsal view (Fig 13D’ and 13E’). Medial area of endophallus with one endophallite.

#### Composition

*Deltochilum aspericolle* Bates, 1870 and at least ten undescribed species.

#### Geographic distribution

The species in this species-group are known to be distributed (Fig 25B), so far, in the following dominions and provinces (in parentheses): Boreal Brazilian (Guianan Lowlands, Roraima, Pantepui), South Brazilian (Madeira and Rondônia) and South-eastern Amazonian (Xingu-Tapajós).

This species-group can be found in several localities in sympatry with the following species-groups (Table 4): *barbipes*, *femorale*, *granulatum*, *guyanense*, *septemstriatum* and *submetallicum*.

**Table 4:** Sympatry of the *aspericolle* species-group with other species-groups by province.

Species-group	Province(s)
*barbipes*	Xingu-Tapajós, Madeira and Rondônia (some localities)
*femorale*	Madeira (one locality, Rondônia, Porto Velho)
*granulatum*	Madeira and Rondônia (several localities)
*guyanense*	Guianan Lowlands, Roraima, Pantepui, Madeira and Xingu-Tapajós (several localities)
*septemstriatum*	Guianan Lowlands (some localities)
*submetallicum*	Guianan Lowlands and Roraima (some localities)

#### Remarks

Females of species of the *aspericolle* species-group that have narrow and discontinuous or consecutively more effaced striae may appear very similar to those of the *barbipes* species-group. Males of species within the *barbipes* species-group with steep tapering (Fig 19F) on posterior margin of metafemur (lacking setae) may appear very similar to those of the *aspericolle* species-group. However, they are easily separated by the following character states: 1) median emargination of the clypeus broadly U-shaped in the *aspericolle* species-group or narrowly U-shaped in the *barbipes* species-group; 2) clypeal teeth separated by at least twice the basal width of a tooth in the *aspericolle* species-group or less than a basal width of a tooth in the *barbipes* species-group; 3) anterior margin of the clypeus concave (Fig 5D) in the *aspericolle* species-group, whereas that of the *barbipes* species-group is flat or lightly concave (Fig 5E and 5F).

In dorsal view, species of the *aspericolle* species-group that bear wide striae (Fig 3C) may appear similar to species of *septemstriatum* species-group (Fig 4H). These can be distinguished by the anterior margin of the clypeus, between clypeal teeth, which is expanded into a triangular shape (Fig 5D) in the *aspericolle* species-group and regular, not expanded, in the *septemstriatum* species-group (Fig 5A). Males belonging to both species-groups can also be separated via their secondary sexual dimorphism. in the *aspericolle* species-group the posterior femur possesses a steep tapering (Fig 19B arrow, and 19C) on the posterior margin whereas in the *septemstriatum* species-group it bears a broad medial expansion (Fig 20C).

Males of the *aspericolle* species-group bearing a weak steep tapering on the posterior margin of metafemur, can be confused as belonging to the *susanae* species-group, *komareki* species-group, or one species (undescribed) of the *lindemannae* species-group. In such cases, specimens of *aspericolle* species-group can be distinguished from species of *susanae* and *komareki* species-groups by the posterior edge of metafemur, which has two margins in *aspericolle* species-group (Fig 10H and 10I), by contrast it bears a single margin (the dorsal), where the ventral surface of metafemur forms a declivity of approximately 45° on the posterior edge (Fig 10E and 10F) in the other two species-groups. The undescribed species of *lindemannae* species-group can be distinguished by the size of the eyes, which are smaller (inter-ocular distance ten to 15 times the width of one eye) in the *aspericolle* species-group than in that species of the *lindemannae* species-group (inter-ocular distance six to eight times width of one eye).

In species of the *aspericolle* species-group with broad striae (Fig 3C), the paramera (of aedeagus) have a straight dorsal edges in lateral view (Fig 13E) and in species with narrow striae (Fig 3B), the dorsal edges of the paramera bear a strong tapering on the apical third followed by broad expansion in lateral view (Fig 13D). The first species are distributed in the Boreal Brazilian dominion (Guianan Lowlands, Roraima) and the latter in South Brazilian (Madeira and Rondônia) and South-eastern Amazonian (Xingu-Tapajós). Those species could belong to different species-groups, but they are left in the same species-group here due the secondary sexual dimorphism.

### The *barbipes* species-group

#### Diagnosis

For all species currently known of *Deltohyboma*, the species in the *barbipes* species-group can be easily separated by the following combination of character states: clypeal median emargination narrowly U-shaped; clypeal teeth separated by less than a basal width of a tooth; anterior margin of the clypeus, between clypeal teeth, flat or slightly concave, and expanded posteriorly into triangular shape (Fig 5E and 5F) (for further information see “Remarks” section of the *aspericolle* species-group).

#### Description

Medium to large-sized species, length 8–14.5mm, humeral width 5.3–9.1mm. Clypeal median emargination narrowly U-shaped. Clypeal teeth separated by less than a basal width of a tooth. Anterior margin of the clypeus, between clypeal teeth, flat or slightly concave, expanded posteriorly into triangular shape (Fig 5E and 5F). Eyes medium-sized, inter-ocular distance ten to 13 times width of one eye. Internal margin of hypomera enlarged towards anterior angle (Fig 7B, arrows). Tubercles at elytral apex on interstriae III, V-VII with all tubercles well developed or with III poorly developed. Interstriae VI-VII with basal tubercles (Fig 3D and 3E, see also Fig 23C and 23D, arrows). Striae conspicuous and narrow (Fig 3D and 3E), width of third stria, in species with the broadest striae, 1/24th of the distance between striae II and III. Metaventrite with a weak or strong posterior excavation, if weak occupying third basal, if strong may occupying third basal to almost reaching middle of the metaventrite length. Ventral surface of protibia without tubercles or carina (Fig 9A–9C and 9J); in some species, males with carina that bears setae. Posterior edge of metafemur with two margins (Fig 10H and 10I). **Male**. Protibial spur broad and foliaceus. Mesotrochanter not modified or with setae. Metatrochanter with basal sinuation or setae. Apex of mesotibia on ventral-internal margin with a large spatulate expansion (Fig 18C). Internal margin of metatibia with large tubercles (see Fig 18E and 18G), in some species also with long setae (see Fig 18H). Posterior edge of metafemur with steep tapering on basal third (Fig 19F), denticle (Fig 19E) or with long setae on basal third (Fig 19D) or almost reaching the entire femur length. Ventrite I expanded posteriorly reaching (see Fig 21) from distal margin of ventrite IV to almost middle of ventrite VI; width of the expansion of ventrite I, on ventrite III, from four to seven times as wide as distance between clypeal teeth. Aedeagus with paramera subtriangular or slender in lateral view (Fig 13F–13H). Paramera with (Fig 13F’ and 13G’) or without (Fig 13H’) short and thin apical-dorsal notch. Medial area of endophallus with one endophallite with shape of long comma (Fig 16C) or almost straight, rarely without endophallites (Fig 16D). Sub-medial area of endophallus with (Fig 16C and 16D) or without raspules.

#### Composition

Eight described species, six valid: *D*. *aureopilosum* Paulian, 1938, *D*. *barbipes* Bates, 1870, *D*. *batesi* Paulian, 1938, *D*. *fuscocupreum* Bates, 1870, *D*. *sericeum* Paulian, 1938, *D*. *peruanum* Paulian, 1938, *D*. *laevigatum* Balthasar, 1939 (junior synonym of *D*. *peruanum*), *D*. *hypocrita* Balthasar, 1939 (junior synonym of *D*. *sericeum*) and at least ten undescribed species.

#### Geographic distribution

The species in this species-group are known to be distributed (Fig 25C), so far in, in the following dominions and provinces (in parentheses): Boreal Brazilian (Imerí and Napo), South Brazilian (Madeira, Rondônia and Ucayali) and South-eastern Amazonian (Xingu-Tapajós). As well as, in the South American transition zone, Paramo province.

This species-group can be found in several localities in sympatry with the following species-groups (Table 5): *aequinoctiale*, *aspericolle*, *femorale*, *genieri*, *granulatum*, *guyanense*, *lindemannae* and *sextuberculatum* and *D*. *inesae* **sp. nov.** (*incertae sedis*).

**Table 5:** Sympatry of the *barbipes* species-group with other species-groups or species by province.

Species-group	Province(s)
*aequinoctiale*	Napo (one locality, Ecuador, Napo, Tena)
*aspericolle*	Xingu-Tapajós, Madeira and Rondônia (some localities)
*genieri*	Napo and Rondônia (several localities)
*granulatum*	Madeira, Rondônia and Ucayali (several localities)
*guyanense*	Imerí, Napo, Ucayali, Rondônia, Madeira and Xingu-Tapajós (several localities)
*lindemannae*	Rondônia and Ucayali (a few localities)
*sextuberculatum*	Xingu-Tapajós and Madeira (few localities)
*D*. *inesae* **sp. nov.** (*incertae sedis*)	Imerí (a few localities)

#### Remarks

Males of species within the *barbipes* species-group with setae on posterior edge of metafemur (Fig 19D) may appear similar to those of the *lindemannae* (Fig 20A and 20B) and *genieri* (Fig 19H) species-groups. Some species within *barbipes* species-group that have the tubercles at elytral apex on interstriae III, V-VII well-developed, may appear similar to *granulatum* species-group. However, these are easily separated by the mentioned character states in the diagnosis; in *granulatum*, *lindemannae* and *genieri* species-groups, the median emargination of the clypeus is broadly is U-shaped; the clypeal teeth are separated by at least by a basal width of a tooth and the anterior margin of the clypeus is concave (Fig 5D).

Also, males of the *barbipes* species-group with denticle on posterior edge of metafemur (Fig 19E) may appear very similar to those of the *submetallicum* species-group (Fig 20E). However, these are easily separated by the anterior margin of the clypeus is concave (Fig 5D) in *submetallicum* species-group, flat or slightly concave (Fig 5E and 5F) in *barbipes* species-group. As well as, by the posterior edge of metafemur with two margins (Fig 10H and 10I) and the denticle is on ventral margin (Fig 19E’, arrow) in *barbipes* species-group; by contrast the metafemur bears one margin, where the ventral surface is continuous to the dorsal margin (Fig 10B and 10C) and the denticle is on dorsal margin (Fig 20E’, arrow) in *submetallicum* species-group.

### The *bidentatum* species-group

#### Diagnosis

This species-group can be distinguished from all other species-groups (except from *morbillosum* species-group) by having only a carina on elytral base on the seventh interstria (Figs 3F, 23A and 23B, arrow) whereas, for the others, the elytral base have two carinae, one on the interstriae VI and the other one on the interstria VII (Fig 23C and 23D, arrows). It can be separated from *morbillosum* species-group by having large elevated shiny points mixed with punctures on the pronotal disc and on the interstriae (Figs 3F and 23A), that shiny points are small and not elevated in *morbillosum* species-group (Figs 4C and 23B).

#### Description

Medium-sized species, length 12–13.8mm, humeral width 8–9.2mm. Clypeal median emargination broadly U-shaped. Clypeal teeth separated by two or 2.5 times basal width of a tooth. Anterior margin of the clypeus, between clypeal teeth, concave and regular, not expanded posteriorly (Fig 5A). Eyes medium-sized, inter-ocular distance nine to eleven times eye width. Internal margin of hypomera not enlarged towards anterior angle (Fig 7A, arrows). Tubercles at elytral apex on interstriae III-VII. Interstria VII with basal carina (Fig 3F and Fig 23A and 23B, arrow). Carina of ninth interstria not reaching middle of elytral length. Striae I-VIII conspicuous and broad (Fig 3F), width third stria approximately 1/15th or 1/20th of the distance between striae II and III. Metaventrite with one or two weak posterior excavations. Ventral surface of protibia with tubercles or carina (Fig 9E–9I). Posterior edge of metafemur with one margin, the dorsal; ventral surface of metafemur forming a decline of approximately 45° on posterior edge (Fig 10E and 10F). **Male**. Ventral surface of protibia with tubercles. Protibial spur broad and apically bifid (Fig 9E). Apex of on ventral-internal margin of protibia with a small denticle. Insertion of metaspur elongate (Fig 11B) or not (Fig 11A), if not elongate spur articulated; if elongate spur either articulated or fused (Fig 11B, arrow). Spur reaching second tarsomere to longer than tarsus. Metafemur more curved than female. Metaventrite with two weak posterior excavations, the basal one occupying approximately metaventral basal fourth, the other one anterior to that and with a small tubercule on anterior part of most anterior excavation. Ventrite I expanded posteriorly (see Fig 21) reaching or surpassing middle of ventrite V. Width of expansion on ventrite III approximately 2.5x as wide as distance between clypeal teeth. Ventrite VI narrow medially. Paramera subtriangular, with dorsal and ventral edges straight in lateral view (Fig 13I). Paramera with internal edge narrowed on apical third in dorsal view (Fig 13I’). Paramera with long and thin anteapical-dorsal notch (Fig 13I’). Ventral membranes of parameres sclerotised medially. Apex of paramera acute in dorsal view (Fig 13I’). Medial area of endophallus with two endophallites (Fig 16E), right endophallite comma “,”-shaped, apex bent forming a hook and largest than left endophallite. Basal circular shape endophallite with ring very thin.

#### Composition

*Deltochilum bidentatum* Burmeister, 1848 **rev. stat.**, *Deltochilum calcaratum* Bates, 1870 and one undescribed species.

#### Geographic distribution

The species in this species-group are known to be distributed (Fig 25D), so far, in the Parana dominion, Atlantic province. This species-group can currently be found in sympatry with the *irroratum* species-group.

#### Remarks

Harold [68] synonymised *D*. *bidentatum* with *D*. *submetallicum* without an explanation, after which other authors [8, 54, 69, 70] cited the species also as a synonym, perhaps due to the fact that the type specimen has not been found as of yet. One female specimen found in MNHN collection that possesses an old original pin, labelled Bras. int, Dep. Cast (handwritten) / bidentatum. Burm. (handwritten) and that matches the original description is considered a syntype of *D*. *bidentatum*. The designation as lectotype will be made in a further paper (in prep.) with the redescription of this species; however here, we recognize *D*. *bidentatum* as a valid species, as it is clearly not close to *D*. *submetallicum*.

### The *femorale* species-group

#### Diagnosis

For all species known of *Deltohyboma*, species in the *femorale* species-group can be distinguished because they are the smallest species (less than 10mm in length) with striae I-VII inconspicuous (Figs 3G and 22A); also, the males can be distinguished by the unique modification of the posterior femur, it bears a broad medial serratulate expansion (Fig 19G), as well as, by the unique shape of the aedeagus, with paramera with apical-lateral cleft (Fig 13J).

#### Description

Small species, length 6.1–9.1mm, humeral width 4–5.8mm. Clypeal median emargination broadly U-shaped. Clypeal teeth separated at least by twice basal width of a tooth. Anterior margin of the clypeus, between clypeal teeth, concave and expanded posteriorly into triangular shape (Fig 5D). Eyes small to medium-sized, inter-ocular distance nine to 17 times width of one eye. Internal margin of hypomera strongly enlarged towards anterior angle (Fig 7C, arrows). Tubercles at elytral base on interstriae VI-VII (Fig 3G, see also Fig 23C and 23D, arrows). Tubercles at elytral apex on interstriae V-VII or III, V-VII. Striae I-VII inconspicuous (Figs 3G and 22A). Metaventrite with a weak posterior excavation, occupying approximately metaventral basal fifth. Ventral surface of the protibia without tubercles or carina (Fig 9A–9C and 9J). Posterior edge of metafemur with two margins (Fig 10H and 10I). **Male**. Protibial spur broad and foliaceus. Apex of mesotibia on ventral-internal margin with a small denticle (Fig 18B, arrow). Internal margin of metatibia with large tubercles (Fig 18G and 18H). Metafemur with a broad medial serratulate expansion on posterior-ventral margin (Fig 19G). Ventrite I expanded posteriorly (see Fig 21), expansion reaching from the middle to the distal margin of ventrite V; width of expansion on ventrite III from twice narrower to slightly wider than distance between clypeal teeth. Paramera subtriangular, with dorsal and ventral edges straight in lateral view (Fig 13J). Apex of paramera variable, but always with an apical-lateral cleft variable in size (Fig 13J). Medial area of endophallus with two endophallites (Fig 16F), right endophallite at least five times larger than left endophallite. Right endophallite comma “,”-shaped, basally broadened bend and apically broadened and bend forming a hook.

#### Composition

*Deltochilum femorale* Bates, 1870 and at least ten undescribed species.

#### Geographic distribution

**Fig 26:**
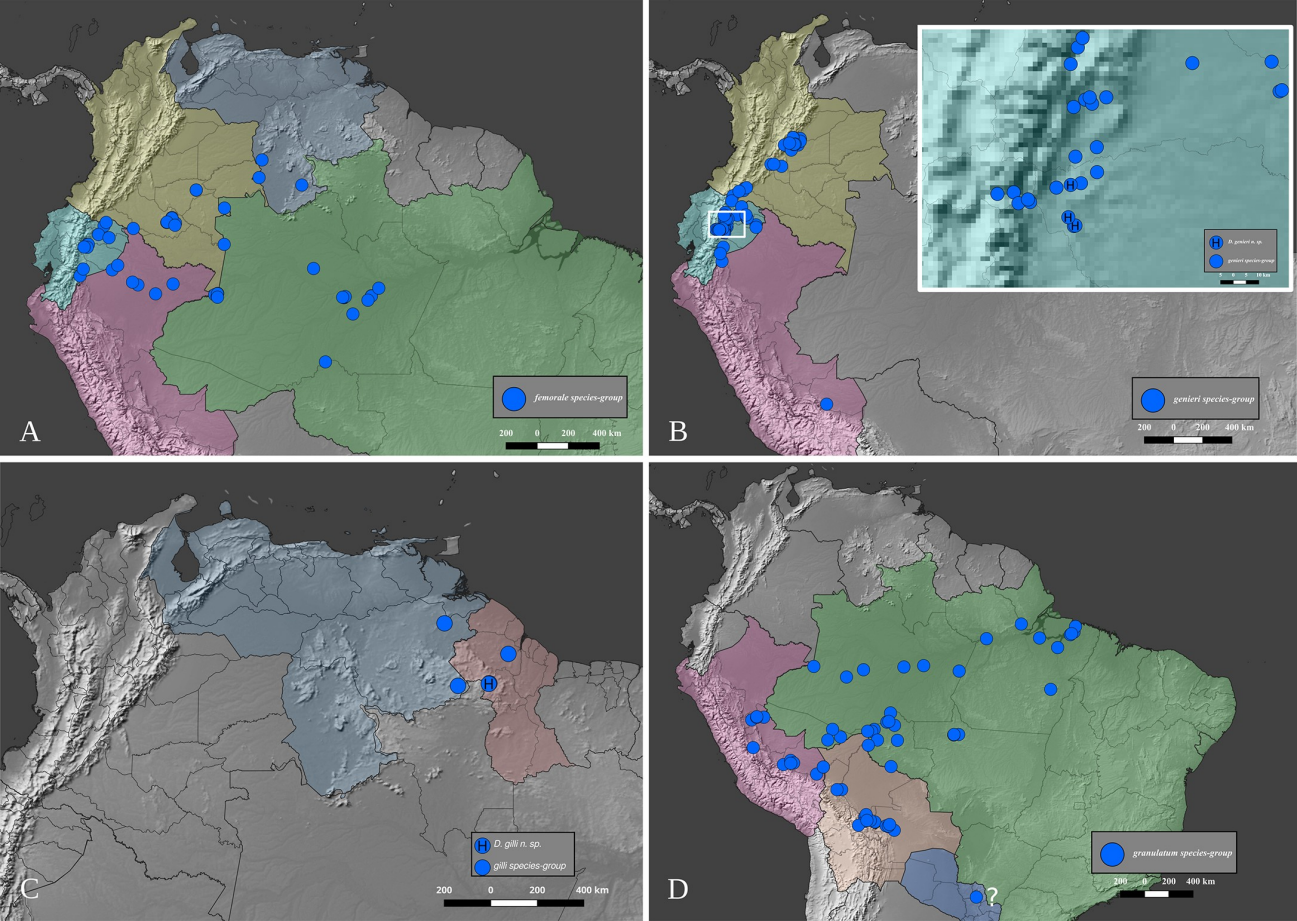
Distribution maps. (A) *femorale* species-group. (B) *genieri* species-group, blue circle with black “H” = holotype and paratypes of *Deltochilum genieri* **sp. nov.** (C) *gilli* species-group, blue circle with black “H” = holotype and paratype of *Deltochilum gilli* **sp. nov.** (D) *granulatum* species-group, “?” showing locality with doubt.

The species in this species-group are known to be distributed (Fig 26A), so far, in the following dominions and provinces (in parentheses): Boreal Brazilian (Pantepui, Imerí and Napo), and South Brazilian (Madeira). As well as, in the South American transition zone, Paramo province.

This species-group can be found in several localities in sympatry with the following species-groups (Table 6): *aspericolle*, *barbipes*, *genieri*, *granulatum* and *guyanense*; also, with *D*. *inesae* **sp. nov.** (*incertae sedis*).

**Table 6:** Sympatry of the *femorale* species-group with other species-groups or species by province.

Species-group	Province(s)
*aspericolle*	Madeira (one locality, Rondônia, Porto Velho)
*barbipes*	Imerí, Napo and Madeira (several localities)
*genieri*	Paramo (a few localities)
*granulatum*	Madeira (few localities)
*guyanense*	Imerí, Napo and Madeira (several localities)
*D*. *inesae* **sp. nov.** (*incertae sedis*)	Imerí (few localities)

### The *genieri* species-group

#### Diagnosis

For all species known of *Deltohyboma*, species in the *genieri* species-group only can be distinguished by the aedeagus, the paramera bear an apical-dorsal sulcus (Fig 14A) (for further information see “Remarks” section of the *barbipes* species-group).

#### Description

Small to Medium-sized species, length 7.5–10.5mm, humeral width 4.8–6.8mm. Clypeal median emargination broadly U-shaped or almost truncated. Clypeal teeth separated by at least twice basal width of a tooth. Anterior margin of the clypeus, between clypeal teeth, concave, expanded posteriorly into triangular in shape (Fig 5D). Eyes large, inter-ocular distance seven to nine times width of one eye. Internal margin of hypomera not enlarged towards anterior angle (Fig 7A, arrows). Tubercles at elytral apex on interstriae III, V-VII with all tubercles well developed or with III poorly developed, or on V-VII. Interstriae VI-VII with basal tubercles (Fig 3H, see also Fig 23C and 23D, arrows). Striae conspicuous and narrow (Fig 3H), width of third stria, in species with the broadest striae, 1/25th of the distance between striae II and III. Metaventrite with a very weak posterior excavation occupying fourth basal. Ventral surface of protibia without tubercles or carina (Fig 9A–9C and 9J). Posterior edge of metafemur with two margins (Fig 10H and 10I). **Male**. Protibial spur broad and foliaceus. Apex of mesotibia on ventral-internal margin with a large spatulate expansion (Fig 18C). Basal third of mesofemur with a sinuation and with or without setae. Metafemur modified, with small to long setae on basal third (Fig 19H) or almost reaching the entire femur length, or with small setae and a small carina on basal third. Internal margin of metatibia with large tubercles (see Fig 18E and 18G). Ventrite I expanded posteriorly (see Fig 21), expansion reaching from distal margin of ventrite IV to almost distal margin of ventrite V; width of the expansion of ventrite I, on ventrite III, from slightly narrower to three times as wide as distance between clypeal teeth. Expansion basally with (Fig 21I, arrow) or without orifice. Aedeagus with paramera subtriangular in lateral view and with an apical-dorsal sulcus (Fig 14A). Medial area of endophallus with one endophallite (Fig 16G).

#### Composition

*Deltochilum genieri* **sp. nov.** and at least 15 undescribed species.

#### Geographic distribution

The species in this species-group are known to be distributed (Fig 26B), so far, in the following dominions and provinces (in parentheses): Pacific (Sabana), Boreal Brazilian (Imerí and Napo) and South Brazilian (Rondônia.). As well as, in the South American transition zone, Paramo province.

This species-group can be found in several localities in sympatry with the following species-groups (Table 7): *aequinoctiale*, *barbipes*, *femorale*, *guyanense* and *plebejum*.

**Table 7:** Sympatry of the *genieri* species-group with other species-groups by province.

Species-group	Province(s)
*aequinoctiale*	Paramo (a few localities)
*barbipes*	Napo and Rondônia (several localities)
*femorale*	Paramo (a few localities)
*guyanense*	Imerí, Napo, and Paramo (several localities)
*plebejum*	Sabana and Paramo (few localities)

#### Remarks

Despite the internal margin of hypomera is regular, not enlarged towards anterior angle (Fig 7A, arrows) in the *genieri* species-group, and it is strongly enlarged towards anterior angle (Fig 7C, arrows) in the *lindemannae* species-group; when it is possible, the best way to separate these species-group is by the aedeagus. The paramera in the *lindemannae* species-group has not an apical-dorsal sulcus (Fig 14G–14I).

### *Deltochilum genieri* sp. Nov

urn:lsid:zoobank.org:act:2058DF85-8255-49A5-8227-17C71E532BE9

(Figs 3H, 14A-14A’, 16G and 26B blue circles with “H”, 27)

### Material examined

#### Holotype

**♂**, **ECUADOR: Pastaza:** Llandia, 17 km N Puyo. remnant rainforest, [1°21'3"S], [77°58'4"W], 1000m, 19.vii.1994, F. Génier coll., carrion trap (CMNC) [WSD00040001]. [aedeagus and endophallus extracted].

#### Paratypes

**ECUADOR: Pastaza:** 22 km SE Puyo. forest, [1°37'11"S], [77°50'40"W], 900m, 2**♂♂**, 12-16.vii.1976, S. Peck coll., carrion trap (CMNC), 2**♂♂**, 12-16.vii.1976, S. Peck coll., dung trap (CMNC), 22km SE Puyo, [1°37'11"S], [77°50'40"W], 900m, **♂**, 12-16.vii.1976, S Peck coll., for.car.tps. 42–43 (BDGC), **♂**, 12-16.vii.1976, S Peck coll., for.dng.tps. 49–41 (BDGC), 25 km NNE Puyo. forest, [1°18'19"S], [77°52'59"W], 1000m, **♂**, 4-13.vii.1976, S. Peck coll., dung trap (CMNC), 9 km ESE Veracruz. forest, [1°33'49"S], [77°53'8"W], 900m, **♂**, 22-24.viii.1975, R. Webster coll., dung trap (CMNC), Llandia, 17 km N Puyo. remnant rainforest, [1°21'3"S], [77°58'4"W], 1000m, 2**♀♀**, 19.vii.1994, F. Génier coll., carrion trap (CMNC), 2**♂♂**, 19.vii.1994, F. Génier coll., feces trap (CMNC), 3**♂♂**, 20.vii.1994, F. Génier coll., feces trap (CMNC).

#### Description

**Fig 27:**
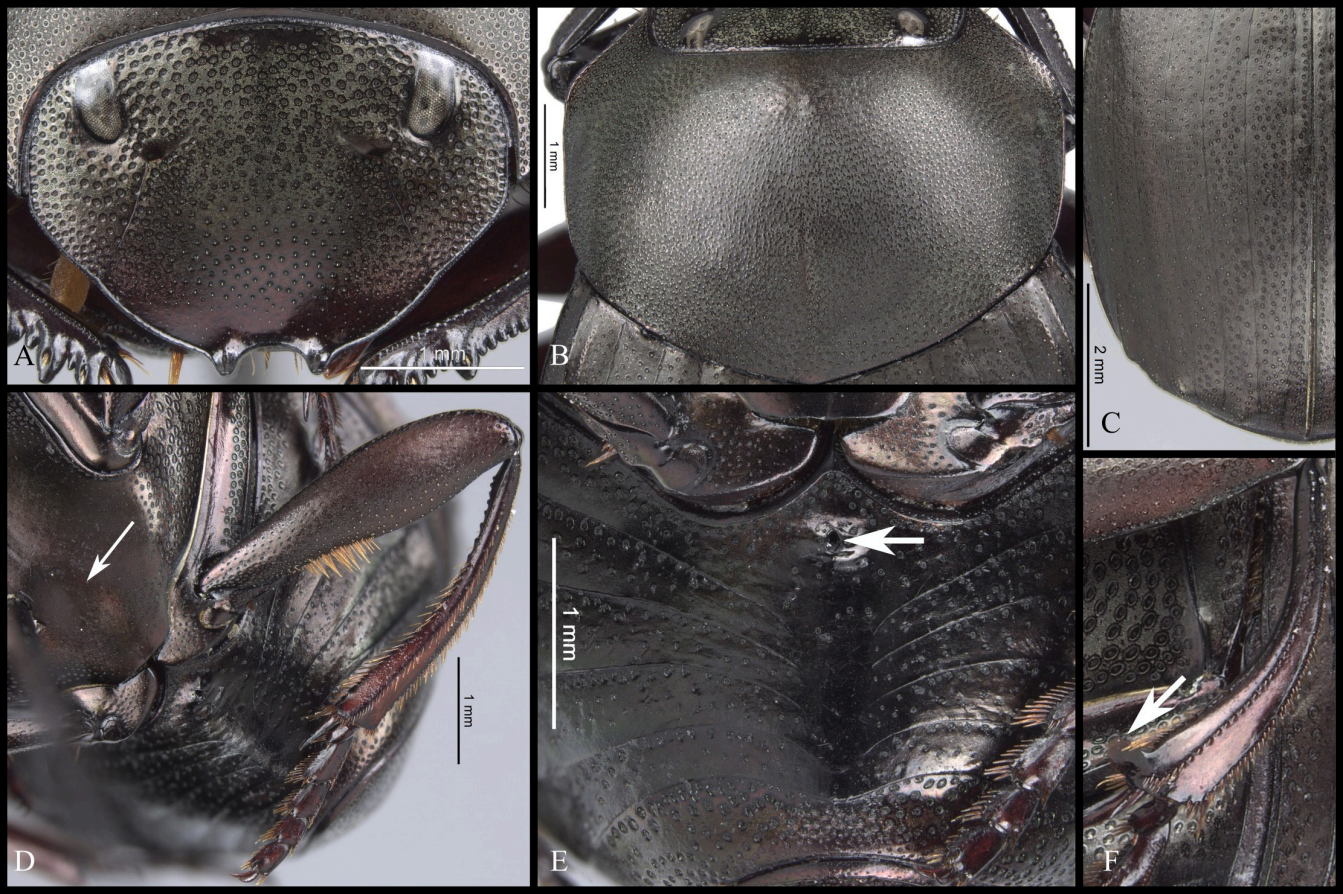
External morphology of the *Deltochilum genieri* sp. nov. Holotype, (A) head. (B) pronotum. (C) elytra. (D) ventral view, arrow showing the metaventral excavation. (E) ventrites, arrow showing the circular orifice of ventrite I. (F) mesotibia, arrow showing the spatulate expansion.

Holotype male, length 8.2mm, humeral width 5mm. Dark green with some red reflections dorsally (Figs 3H, 27B and 27C). Black ventrally, with shiny red reflections on hypomera, metaventrite, metaventral process, mid- and hind legs and ventrite VI. **Head** (Fig 27A). Inter-ocular distance seven times width of one eye. Clypeal median emargination broadly U-shaped. Clypeal teeth separated approximately by 2 times basal width of a tooth. Anterior margin of the clypeus, between clypeal teeth, concave and expanded posteriorly into triangular shape. Punctures on frons separated by less than one diameter of each puncture and subequal in size to head disc. Disc punctures separated by one or less than one diameter of each puncture. Punctures from disc towards anterior area successively smaller. Genal punctures subequal in size to disc punctures and separated by less than one diameter. **Pronotum** (Fig 27B). Edge between anterior and medial-lateral angle almost straight. Medial-lateral angle of pronotum rounded. Disc punctures half size to anterior-lateral ones. Basal punctures smaller than anterior-lateral ones. Disc with shiny points subequal to discal punctures. **Hypomera**. Internal margin not enlarged towards anterior angle. **Elytra** (Fig 27C). Carina of the ninth interstria not reaching middle of elytral length. Interstriae VI and VII with basal tubercles almost identical in size with approximately three times smaller than ninth carina. Elytral apex o interstriae III, V-VII with tubercles. Striae I-VII conspicuous and very thin, width third stria on disc approximately 1/50th of the distance between striae II and III. First stria twice as wide as second. Stria VIII conspicuous apical and laterally and not reaching carina of the ninth interstria. Stria VIII laterally twice wide to seventh stria. Punctures on interstriae approximately separated by one diameter, on intestria II slightly denser. Punctures of third interstria on disc occupying about 1/9th of the distance between striae II and III. **Metaventrite** (Fig 27D). Disc with few deep posterior excavation, occupying metraventral basal third (Fig 27D, arrow). Disc with few and small punctures, conspicuous punctures at 8x magnification. Disc punctures four times smaller than punctures on anterior-lateral area of metaventral process. Anterior-central and anterior-lateral areas of metaventral process with punctures almost contiguous. Anterior-medial area of metaventral process with few and small punctures. **Legs**. Protibial spur broad and foliaceus. Ventral surface of protibia without carina or tubercles. Mesotrochanter not modified. Metatrochanter with a tuft of setae (Fig 27E). Femora with punctures separated by less than one diameter. Mesofemur with a sinuosity basal on posterior margin. Posterior edge of metafemur with two margins. Metafemur with setae on basal third of posterior margin (Fig 27D). Setae anterior to posterior-ventral margin. Apex of mesotibia on ventral-internal margin with large spatulate expansion (Fig 27F, arrow). Metatibial spur articulated and larger than first metatarsomere. Internal margin of metatibia with large tubercles, tubercles occupying almost all metatibial length (Fig 27D). **Abdomen** (Fig 27E). Ventrite I expanded posteriorly, expansion reaching the distal margin of fourth ventrite. Width of expansion of ventrite I, on ventrite III, slightly narrower than distance between clypeal teeth. Margins of expansion between ventrites II-IV almost parallel. Apex of expansion rounded. Ventrite I on middle with a circular orifice (Fig 27E, arrow). Distal margin of ventrite V slightly sinuated. Ventrite VI narrowed medially. **Pygidium**. Discal punctures slightly extended transversely and separated by less than one diameter. **Genitalia**. Paramera subtriangular and straight dorsally, curved ventrally in lateral view (Fig 14A). Apex of paramera truncated in dorsal view (Fig 14A’). Paramera with an apical-dorsal sulcus occupying 1/5th of parameral length (Fig 14A). Medial area of endophallus (Fig 16G) with one endophallite with rectangular shape. Basal circular shape endophallite with ring thin. Sub-medial area of endophallus with scales.

#### Female

Profemur with spur thinner than male and spiniform. Meso- and metafemur not modified. Apex of mesotibia narrower than male and without spatulate expansion. Metasternal disc with posterior excavation smaller than male. Punctures on metasternal disc smaller than male. Medially ventrite V slightly narrower than ventrite VI. Ventrite VI only slightly narrowed medially.

#### Etymology

A patronym, noun in the genitive case, for François Génier, an excellent and prolific scarabaeoidologist and taxonomist. Collector of the Holotype and part of type series as well as of several specimens of the species-group that gives its the name (*genieri* species-group). See also the “Acknowledgments” section.

**Remarks.** There is some variation in terms of size and density of the punctures on the posterior area of the pronotum and on the interstriae.

**Known distribution** (Fig 26B, blue circles with “H”)

ECUADOR. **Pastaza**: 25 km NNE Puyo. Llandia, 17 km N Puyo. 9 km ESE Veracruz. 22 km SE Puyo.

### The *gilli* species-group

#### Diagnosis

This species-group shares the following combination of character states with the *aspericolle*, *femorale*, *genieri*, *lindemannae* species-groups and *Deltochilum inesae* **sp. nov.** (*incertae sedis*): anterior margin of the clypeus, between clypeal teeth, concave and expanded posteriorly into triangular shape (Fig 5D) and posterior edge of metafemur with two margins (Fig 10H and 10I). However, it can be distinguished by the ventral surface of the protibia, which has a weak carina (Fig 9D), that it is not present (Fig 9A–9C and 9J) in the species and the species-groups mentioned above as well as the shape of aedeagus (Fig 14B).

Furthermore, it can be easily separated from the *femorale* species-group by the body size 6.1–9.1mm in length, whereas it is 8–8.4mm in length in the *gilli* species-group; as well as by their secondary sexual dimorphism, in which the posterior femur has a steep tapering on the posterior margin with an expansion before the steep tapering in the *gilli* species-group (Fig 19I) whereas it bears a broad medial serratulate expansion in the *femorale* species-group (Fig 19G).

Otherwise, it can be easily separated from *D*. *inesae* **sp. nov.** (*incertae sedis*) by the pronotum, which has shiny points in the *gilli* species-group and completely absents in *D*. *inesae* **sp. nov.** (*incertae sedis*). Also, males of *D*. *inesae* **sp. nov.** (*incertae sedis*) the posterior edge of mesofemur has an acute basal expansion, whereas the mesofemur in the *gilli* species-group is regular, without expansion.

#### Description

Medium-sized species, length 8–8.4mm, humeral width 5.1–5.2mm. Clypeal median emargination broadly U-shaped. Clypeal teeth separated approximately by 1.5 times basal width of a tooth. Anterior margin of the clypeus between clypeal teeth concave and expanded posteriorly into triangular shape (Fig 5D). Eyes large, inter-ocular distance seven to nine times width of one eye. Internal margin of hypomera strongly enlarged towards anterior angle (Fig 7C, arrows). Pronotal disc with shiny points well defined or irregular, separated between them, contiguous or separated to punctures. Tubercles at elytral base on interstriae VI-VII (Fig 3I, see also Fig 23C and 23D, arrows). Tubercles at elytral apex on interstriae III-VII or III, V-VII. Striae I-VIII inconspicuous even apically, only in some parts striae slightly visible and very narrow or I-VII conspicuous (Fig 3I) and narrow, width of third stria 1/33th or 1/40th of the distance between striae II and III. Metaventrite with a weak posterior excavation, occupying approximately the metaventral basal fourth. Ventral surface of protibia with a weak carina (Fig 9D). Posterior edge of metafemur with two margins (Fig 10H and 10I). **Male**. Protibial spur broad and foliaceus. Mesofemur modified, with a slight sinuosity on apical third. Apex of mesotibia on ventral-internal margin with a small or large spatulate expansion (Fig 18C, arrow). Metatrochanter modified or not, if modified with an expansion on distal third. Metafemur on basal third of posterior edge with steep tapering and expansion before that steep tapering (Fig 19I). Internal margin of metatibia with small or large tubercles (see Fig 18E, 18G and 18H). Ventrite I expanded posteriorly (see Fig 21); expansion reaching from the middle of ventrite IV to almost the distal margin of ventrite V; width of expansion on ventrite III variable, narrower to wider than distance between clypeal teeth. Paramera subtriangular, with dorsal and ventral edges straight in lateral view (Fig 14B). Apex of paramera rounded in dorsal view (Fig 14B’). Paramera with short and thin apical-dorsal notch (Fig 14B’). Medial area of endophallus with one endophallite (Fig 16H). Basal circular shape endophallite with ring very thin and handle strongly broadened medially.

#### Composition

*Deltochilum gilli* **sp. nov.** and four undescribed species.

#### Geographic distribution

The species in this species-group are known to be distributed (Fig 26C), so far, in the Boreal Brazilian dominion, Guianan Lowlands and Pantepui provinces.

This species-group can currently be found in sympatry with *lindemannae* species-group in few localities of the Pantepui province and with *septemstriatum* species group in one locality (Guyana, Cuyuni-mazaruni, Takutu Mountains) of the Guianan Lowlands province.

#### Remarks

In dorsal view, species of the *gilli* species-group may appear very similar to those of the *genieri* (Fig 3H) and the *lindemannae* (Fig 4B) species-groups; but these are easily separated by the punctures on head; the punctures on head disc are subequal or slightly larger than punctures between clypeal teeth in the *genieri* and the *lindemannae* species-groups, whereas the punctures on disc are at least two times larger than those between clypeal teeth in the *gilli* species-group. Males of these species-groups can also be separated by their secondary sexual dimorphism, in which the posterior femur has a steep tapering on the posterior margin in the *gilli* species-group (Fig 19I). Whereas it is regular, without steep tapering, and bearing setae in the *genieri* species-group (Fig 19H) and in the most species known in the *lindemannae* species-group (Fig 20A and 20B).

The secondary sexual dimorphism in the *gilli* species-group is very similar to that of the *aspericolle* species-group (Fig 19B and 19C) by male having a steep tapering on the posterior margin. However, these are easily separated by the size of the eyes. In the *aspericolle* species-group the inter-ocular distance is over nine times width of one eye whereas, in the *gilli* species-group, the inter-ocular distance is under nine times the width of one eye.

#### *Deltochilum gilli* sp. Nov

urn:lsid:zoobank.org:act:FE0A1150-C13E-4735-B69E-3ED6CF923C5E

(Figs 26C blue circle with “H” and 28)

### Material examined

#### Holotype

**♂**, **GUYANA: District 8:** Mount Wokomung. 1° forest, 5°06'34.8"N, 59°49'15.3"W, 1234m, 27.x-1.xi.2004, B Hubley coll., Pitfall trap (human dung) (BDGC). [aedeagus and endophallus extracted]. It will be deposited at (CNCI) (Bruce Gill pers. comm.).

#### Paratype

**♀, GUYANA: District 8:** Mount Wokomung. 1° forest, 5°06'34.8"N, 59°49'15.3"W, 1234m, 27.x-1.xi.2004, B Hubley coll., Pitfall trap (human dung) (BDGC).

#### Description

**Fig 28:**
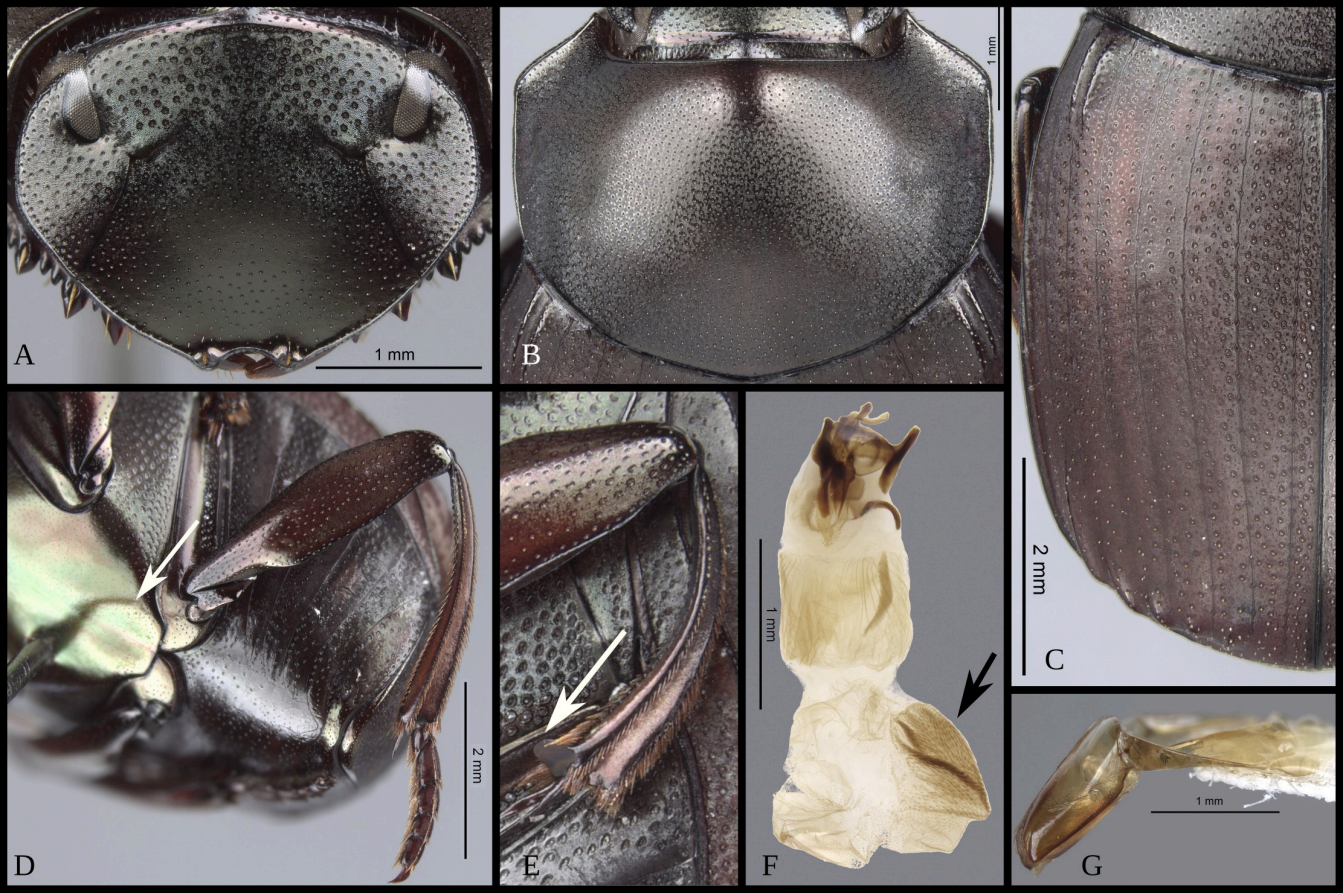
External morphology of the *Deltochilum gilli* sp. nov. Holotype, (A) head. (B) pronotum. (C) elytra. (D) ventral view, arrow showing the metaventral excavation. (E) mesotibia, arrow showing the spatulate expansion. (F) endophallus, arrow showing elongate scales on sub-medial area. (G) aedeagus lateral view.

Holotype male, length 8mm, humeral width 5.1mm. Brown dorsally (Fig 28), with some light brown elytra. Brown ventrally, with shiny red and green reflections on metaventral disc, meso- and metafemora and ventrite VI. **Head** (Fig 28A). Dorsal inter-ocular distance approximately nine times width of one eye. Clypeal median emargination broadly U-shaped. Clypeal teeth separated approximately by 1.5 times basal width of a tooth. Anterior margin of the clypeus, between clypeal teeth, concave and expanded posteriorly into triangular shape. Punctures on frons separated by one or less than one diameter of each puncture. Punctures on head disc separated by one diameter of each puncture. **Pronotum** (Fig 28B). Edge between anterior and medial-lateral angle subconcave. Medial-lateral angle rounded. Punctures almost with the same size, basal punctures only slightly larger than discal punctures. Punctures on the disc separated by one diameter. Shiny points on disc well defined and separated from punctures. **Hypomera**. Internal margin strongly enlarged towards anterior angle. **Elytra** (Fig 28C). Carina of the ninth interstria reaching middle of elytral length. Striae I-VII conspicuous. First stria almost twice as wide as second stria. Striae III-VII subequal in width. Width third stria approximately 1/33th of the distance between striae II and III. Stria VIII conspicuous apical and laterally; but discontinuous in some parts and reaching the apex of carina of the ninth interstria. Punctures of second and third interstriae on disc separated by one or more than one diameter. Punctures of third interstria on disc occupying about 1/10th of the distance between striae II and III. Interstriae with shiny points mixed with the punctures. Apical tubercles on interstriae III-VII (Fig 28C). **Metaventrite** (Fig 28D). With a weak posterior excavation, occupying approximately the metaventral basal fourth (Fig 28D arrow). Disc with conspicuous punctures at 8x magnification. Disc punctures at least half the size of punctures on anterior-lateral area of metaventral process and dispersed separated at least by three diameters. Punctures on anterior-lateral area of metaventral process punctures separated by less than one diameter. Anterior-central area of metaventral process with few and smaller punctures than anterior-lateral ones. **Abdomen**. Width of expansion of the ventrite I, on ventrite III, wider than the distance between clypeal teeth; expansion almost reaching distal margin of ventrite V. Margins of expansion between ventrites II-IV almost parallel, on V forming an acute angle. Apex of expansion truncate. Basal area of expansion with punctures separated by one o less than one diameter. **Legs**. Protibial spur broad and foliaceus. Ventral surface of protibia with a weak carina. Posterior edge of metafemur with two margins. Apex of mesotibia on ventral-internal margin with a large spatulate expansion (Fig 28E, arrow). Metatrochanter with an expansion on distal third. Expansion of metafemur 1.5x as wide as the width of metafemur basal to expansion (Fig 28D). Internal margin of metatibia with small tubercles, occupying the basal half (Fig 28D). **Pygidium**. Punctures separated by one diameter. **Genitalia**. Paramera subtriangular, with dorsal and ventral edges straight in lateral view (Fig 28G). Apex of paramera rounded in dorsal view. Paramera with short and thin apical-dorsal notch. Medial area of endophallus with one endophallite (Fig 28F). Medial endophallite slightly broadened medially. Basal circular shape endophallite with ring very thin and handle strongly broadened medially. Sub-medial area of endophallus with elongate scales (Fig 28F, arrow).

**Female**: Profemur with spur thinner than male and spiniform. Meso- and metafemur not modified. Apex of mesotibia narrower than male and without spatulate expansion. Metasternal disc with posterior excavation smaller than male. Medially ventrite V as wide as ventrite VI. Ventrite VI only slightly narrowed medially.

#### Remarks

This species is currently only known from two specimens, both teneral with the elytra and pygidium poorly sclerotised. The paratype differs from the holotype by the sexual dimorphism and by having the pygidium less sclerotised.

#### Etymology

A patronym, noun in the genitive case, for Bruce Gill, a great and prolific scarabaeiodologist and taxonomist. Collector of the several specimens of *gilli* species-group. See also the “Acknowledgments” section.

**Known distribution** (Fig 26C, blue circle with “H”)

GUYANA. District 8, Mount Wokomung.

### The *granulatum* species-group

#### Diagnosis

For all species known of *Deltohyboma*, males in the *granulatum* species-group can be distinguished by the metafemur, in which the posterior edge has one margin, the dorsal, where the ventral surface is continuous to that dorsal margin (Fig 10B and 10C), additionally bearing a basal weak carina (Fig 19J and 19J’, arrow) (for further information see “Remarks” section of the *barbipes* species-group).

#### Description

Small to Medium-sized species, length 8.5–10.5mm, humeral width 6.3–7.2mm. Clypeal median emargination broadly U-shaped. Clypeal teeth separated by 1.5 times the basal width of a tooth. Anterior margin of the clypeus, between clypeal teeth, concave and expanded posteriorly into triangular shape (Fig 5D). Eyes medium-sized, inter-ocular distance eight to 14 times width of one eye. Internal margin of hypomera enlarged towards anterior angle (Fig 7B, arrows). Tubercles at elytral apex on interstriae III, V-VII with all tubercles well developed and III very large and protruded. Interstriae VI-VII with basal tubercles (Fig 3J, see also Fig 23C, 23D, arrows). Width of first stria twice as wide as second. Striae I-II conspicuous, III-VII narrow and effaced, successively narrower and more effaced (Fig 3J), with VII almost inconspicuous. Metaventrite with a very weak posterior excavation occupying fourth basal. Ventral surface of protibia without tubercles or carina (Fig 9A–9C and 9J). Posterior edge of metafemur with one margin, the dorsal; ventral surface of metafemur continuous to the dorsal margin (Fig 10B and 10C). **Male.** Protibial spur broad and foliaceus. Apex of mesotibia on ventral-internal margin a large spatulate expansion (Fig 18C). Metafemur with a weak carina on basal third (Fig 19J and 19J’, arrow). Internal margin of metatibia with large tubercles (see Fig 18E and 18G). Ventrite I expanded posteriorly (see Fig 21), expansion almost reaching distal margin of ventrite V; width of the expansion of ventrite I, on ventrite III, from four to five times as wide as distance between clypeal teeth. Aedeagus (Fig 14C) with paramera subtriangular with dorsal and ventral edges straight in lateral view. Paramera with short and thin apical-dorsal notch (Fig 14C’). Apex formed by the paramera truncated in dorsal view (Fig 14C’). Medial area of endophallus with one endophallite with shape of long comma “,”.

#### Composition

*Deltochilum granulatum* Bates, 1870 and at least five undescribed species.

#### Geographic distribution

The species in this species-group are known to be distributed (Fig 26D), so far, in the following dominions and provinces (in parentheses): Boreal Brazilian (Pará and Roraima), South Brazilian (Madeira, Rondônia and Ucayali) and South-eastern Amazonian (Xingu-Tapajós).

This species-group can be found in several localities in sympatry with the following species-groups (Table 8): *aspericolle*, *barbipes*, *femorale*, *guyanense*, *irroratum*, *lindemannae*, *septemstriatum sextuberculatum* and *submetallicum*.

**Table 8:** Sympatry of the *granulatum* species-group with other species-groups by province.

Species-group	Province(s)
*aspericolle*	Madeira and Rondônia (several localities)
*barbipes*	Madeira, Rondônia and Ucayali (several localities)
*femorale*	Madeira (few localities)
*guyanense*	Pará, Roraima, Xingu-Tapajós, Madeira (several localities)
*irroratum*	Rondônia (few localities)
*lindemannae*	Rondônia (one locality, Bolivia, Cochabamba, 124km E Cochabamba)
*septemstriatum*	Roraima (one locality, Brazil, Pará Monte Dourado)
*sextuberculatum*	Pará and Xingu-Tapajós (a few localities)
*submetallicum*	Roraima (few localities)

### The *guyanense* species-group

#### Diagnosis

For all species known of *Deltohyboma*, males in the *guyanense* species-group can be distinguished by the unique modification of the posterior femur, it has a strong steep tapering on the posterior margin forming a broadly dentiform structure (Fig 19K); also by the unique shape of the aedeagus (Fig 14D).

#### Description

Medium-sized species, length 10.2–13.3mm, humeral width 6.3–9.1mm. Clypeal median emargination broadly U-shaped. Clypeal teeth separated approximately by twice the basal width of a tooth. Anterior margin of the clypeus between clypeal teeth concave, regular, slightly expanded or expanded posteriorly, but not triangular in shape (Fig 5A–5C). Eyes large, inter-ocular distance seven to ten times width of one eye. Internal margin of hypomera not enlarged towards anterior angle (Fig 7A, arrows). Tubercles at elytral apex on interstriae with following variations: 1) III, V-VII with all tubercles well developed, 2) III, V-VII with III few developed. 3) V-VII with all tubercles well developed. Interstriae VI-VII with basal tubercles (Fig 3K, see also Fig 23C and 23D, arrows). Striae inconspicuous (Figs 3K and 22C), even striae IX and X. Metaventrite with a very weak posterior excavation occupying fourth basal. Ventral surface of protibia with a weak carina (Fig 9D). Posterior edge of metafemur with two margins (Fig 10H and 10I). **Male**. Protibial spur broad and foliaceus. Apex of mesotibia on ventral-internal margin with a large spatulate expansion (Fig 18C). Posterior edge of metafemur with a steep tapering almost medially, forming a broadly dentiform structure (Fig 19K). Internal margin of metatibia with large tubercles (see Fig 18E and 18G). Ventrite I expanded posteriorly (see Fig 21), expansion reaching from distal margin of ventrite IV to distal margin of ventrite V; width of the expansion of ventrite I, on ventrite III, from slightly wider to twice as wide as distance between clypeal teeth. Expansion basally with (Fig 21I, arrow) or without orifice. Aedeagus with parameres subrectangular in lateral view (Fig 14D). Parameres broadened toward apex and apex of paramera truncate in dorsal view (Fig 14D’). Parameres flattened in ventral view (Fig 14D). Medial area of endophallus with one endophallite more or less “L”-shaped (Fig 16I). Sub-medial area of endophallus with or without raspules.

#### Composition

Five described species, four valid *D*. *guyanense* Paulian, 1933, *D*. *crenulipes* Paulian, 1938, *D*. *howdeni* Martínez, 1954, *D*. *laetiusculum* Bates, 1870, *D*. *obenbergeri* Balthasar, 1939 (junior synonym of *D*. *crenulipes*) and at least 20 undescribed species.

#### Geographic distribution

**Fig 29:**
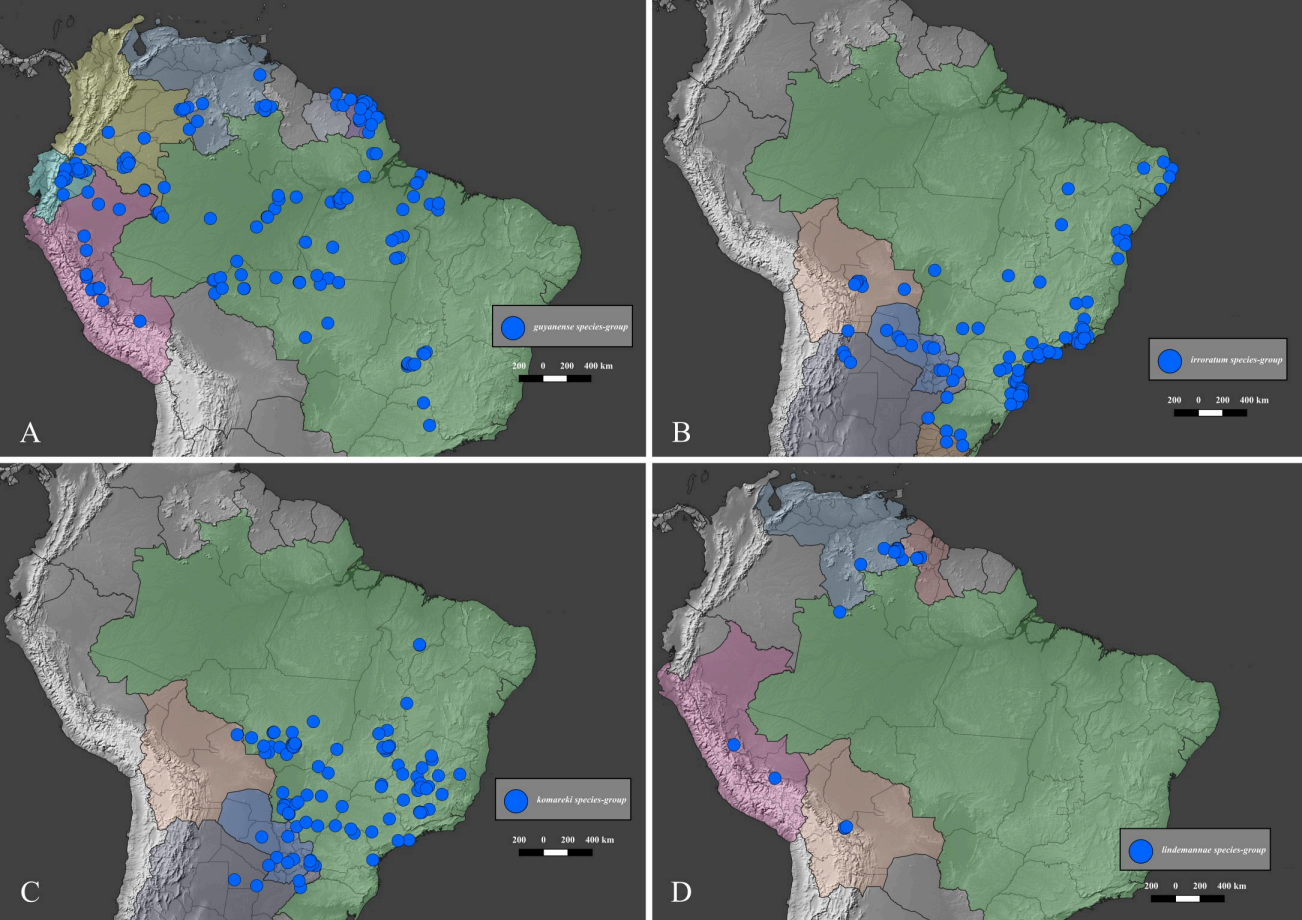
Distribution maps. (A) *guyanense* species-group. (B) *irroratum* species-group. (C) *komareki* species-group. (D) *lindemannae* species-group.

The species in this species-group are known to be distributed (Fig 29A), so far, in the following dominions and provinces (in parentheses): Boreal Brazilian (Pará, Guianan Lowlands, Roraima, Pantepui, Imerí and Napo), South Brazilian (Madeira, Rondônia and Ucayali), South-eastern Amazonian (Xingu-Tapajós), Chacoan (Cerrado). As well as, in the South American transition zone, Paramo province.

This species-group can be found in several localities in sympatry with the following species-groups (Table 9): *aspericolle*, *barbipes*, *femorale*, *genieri*, *granulatum*, *komareki*, *septemstriatum*, *sextuberculatum* and *submetallicum*. Also with *D*. *inesae* **sp. nov.** (*incertae sedis*). Species of *guyanense* and *susanae* species-group may be found in sympatry; species of these species-groups were collected in Colombia, Vichada, Cumaribo, Selva de Matavén, but not in the same locality.

**Table 9:** Sympatry of the *guyanense* species-group with other species-groups or species by province.

Species-group	Province(s)
*aspericolle*	Guianan Lowlands, Roraima, Pantepui, Madeira and Xingu-Tapajós (several localities)
*barbipes*	Imerí, Napo, Ucayali, Rondônia, Madeira and Xingu-Tapajós (several localities)
*femorale*	Imerí, Napo, Paramo and Madeira (several localities)
*genieri*	Imerí, Napo and Paramo (several localities)
*granulatum*	Pará, Roraima, Xingu-Tapajós and Madeira (several localities)
*komareki*	Cerrado (few localities)
*septemstriatum*	Guianan Lowlands and Roraima (some localities)
*sextuberculatum*	Pará, Xingu-Tapajós and Cerrado (few localities)
*submetallicum*	Guianan Lowlands and Roraima (some localities)
*D*. *inesae* **sp. nov.** (*incertae sedis*)	Imerí (few localities)

#### Remarks

In dorsal view, *guyanense* species-group (Fig 3K) can be confused with *femorale* species-group (Fig 3G) and some species in *aequinoctiale*, *gilli*, *irroratum*, *lindemannae* and *parile* species-groups by having inconspicuous striae. From *femorale* species-group can be easily separated by striae IX and X conspicuous, which are inconspicuous in *guyanense* species-group. Also, from *femorale*, *gilli* and *lindemannae* species-groups by the anterior margin of the clypeus, between clypeal teeth, concave and expanded posteriorly into triangular shape in these three species-groups (Fig 5D); regular or slightly expanded posteriorly, but not triangular in shape in *guyanense* species-group (Fig 5A–5C).

Moreover, the *guyanense* species-group shares, with the species that have inconspicuous striae in the *aequinoctiale*, *irroratum*, and *parile* species-groups, the shape of the anterior margin of the clypeus, between clypeal teeth, concave and regular, or slightly expanded, but not triangular in shape (Fig 5A–5C). However, it can be easily distinguished from the species in the aforementioned species-groups by the metafemur with two margins in *guyanense* species-group (Fig 10H and 10I), whereas in those species the metafemur only have one margin (Fig 10E and 10F).

### The *irroratum* species-group

#### Diagnosis

For all species known of *Deltohyboma*, species in the *irroratum* species-group can be distinguished by the metatarsomeres II and III that are each almost as long as broad (Fig 11A), combined with large posterior excavation of metasternal disc (Fig 8C); also, males can be distinguished by the unique endophallus, which lacks endophallites on medial area, combined with large raspules on sub-medial area (Fig 16J).

#### Description

Medium to large species, length 12.1–14.3mm, humeral width 8–9.4mm. Clypeal median emargination broadly U-shaped. Clypeal teeth separated by at least by twice basal width of a tooth. Anterior margin of the clypeus, between clypeal teeth, concave, slightly expanded posteriorly, but not triangular in shape (Fig 5B and 5C). Eyes medium-sized, inter-ocular distance eight to 15 times width of one eye. Internal margin of hypomera enlarged towards anterior angle (Fig 7B, arrows). Tubercles at elytral apex on interstriae III, V-VII; tubercle on III approximately triangular in shape. Interstriae VI-VII with basal tubercles (Fig 23C, arrows); interstria VII with basal tubercle, on interstria VI with a basal hump (no fully developed tubercle) smaller than tubercle on VII, but almost with the same shape (Fig 3L). Striae I-VII conspicuous (Fig 3L) or inconspicuous, but in both stria VIII conspicuous reaching carina of the ninth interstria. If conspicuous narrow or broad, width of the third stria between 1/12th and 1/30th of the distance between striae II and III. If Striae I-VII inconspicuous, area between stria punctures elevated, then elytra with tessellate sculpture. Metaventrite with a large posterior excavation surpassing or reaching the middle of metaventral length. Ventral surface of protibia with tubercles or carina (Fig 9E–9I). Posterior edge of metafemur with one margin, the dorsal; ventral surface of metafemur forming a decline of approximately 45° on posterior edge (Fig 10E and 10F). **Male**. Protibial spur broad and foliaceus. Ventral surface of protibia with tubercles. Mesofemur with a basal sinuation and setae. Apex of mesotibia on ventral-internal margin with a large spatulate expansion (Fig 18C). Insertion of metaspur elongate (Figs 11B and 18E) or not (Figs 11A and 18F), if not elongate spur articulated; if elongate, insertion reaching distal margin of tarsomere I to longer than tarsus and spur either articulated or fused (Fig 18E, arrow). Metaventral excavation larger than wide and surpassing middle of metaventral length. Metafemur more curved than female. Ventrite I expanded posteriorly (see Fig 21), expansion reaching from distal margin of ventrite II to distal margin of ventrite V, or slightly expanded not reaching distal margin of ventrite II. Parameres lightly slender in lateral view (Fig 14E). Parameres narrowed towards apex on internal edge and apex of paramera truncated in dorsal view (Fig 14E’). Paramera with long and thin anteapical-dorsal notch (Fig 14E’). Medial area of endophallus without endophallites and with at least one raspule on sub-medial area (Fig 16J).

#### Composition

Eight described species: *D*. *irroratum* (Castelnau, 1840), *D*. *multicolor* Balthasar, 1939, *D*. *sculpturatum* Felsche, 1907, *D*. *elongatum* Felsche, 1907, *D*. *silphoides* Balthasar, 1939, *D*. *inaequale* Balthasar, 1939, *D*. *viridicupreum* Balthasar, 1939, *D*. *mourei* Pereira, 1949, and at least 13 undescribed species.

#### Geographic distribution

The species in this species-group are known to be distributed (Fig 29B), so far, in the following dominions and provinces (in parentheses): Parana (Atlantic, Parana Forest and Araucaria Forest), Chacoan (Caatinga, Cerrado, Chacoan and Pampean) and South Brazilian (Rondônia).

This species-group can be found in several localities in sympatry with the following species-groups (Table 10): *bidentatum*, *granulatum*, *komareki*, *morbillosum* and *sextuberculatum*.

**Table 10:** Sympatry of the *irroratum* species-group with other species-groups by province.

Species-group	Province(s)
*bidentatum*	Atlantic (few localities)
*granulatum*	Rondônia (few localities)
*komareki*	Atlantic, Parana Forest, Cerrado and Rondônia (a few localities)
*morbillosum*	Atlantic, Parana Forest, (some localities)
*sextuberculatum*	Atlantic, Cerrado and Rondônia (few localities)

### The *komareki* species-group

#### Diagnosis

For all species known of *Deltohyboma*, males in the *komareki* species-group can be distinguished by the metafemur, in which the posterior edge has one margin, the dorsal, where the ventral surface forms a decline of approximately 45° on posterior edge (Fig 10E and 10F), additionally bearing a broad basal sub-quadrate expansion (Fig 19L), can be bifurcated in some specimens (for further information see “Remarks” section of the *aspericolle* species-group).

#### Description

Medium-sized species, length 10.2-12mm, humeral width 7.5-8mm. Clypeal median emargination broadly U-shaped. Clypeal teeth separated by at least twice basal width of a tooth. Anterior margin of the clypeus, between clypeal teeth, concave and expanded posteriorly into triangular shape (Fig 5D). Eyes small, inter-ocular distance from eleven to 20 times width of one eye. Internal margin of hypomera enlarged towards anterior angle (Fig 7B, arrows). Tubercles at elytral apex on interstriae III, V-VII with all tubercles well developed or III-VII 3–7 with IV poorly developed. Interstriae VI-VII with basal tubercles (Fig 4A, see also Fig 23C and 23D, arrows). Striae conspicuous and broad (Fig 4A), width of third stria, in species with the narrowest striae, 1/13th of the distance between striae II and III. Ventral surface of protibia with a weak carina (Fig 9D). Posterior edge of metafemur with one margin, the dorsal; ventral surface of metafemur forming a decline of approximately 45° on posterior edge (Fig 10E and 10F). Metaventrite without posterior excavation. **Male**. Protibial spur broad and foliaceus. Mesofemur slightly sinuate basally. Apex of mesotibia on ventral-internal margin with a large spatulate expansion (Fig 18C). Metafemur modified, posterior edge with a broad basal sub-quadrate expansion (Fig 19L), can be bifurcated in some specimens. Internal margin of metatibia with small or large tubercles. Ventrite I expanded posteriorly (See Fig 21), expansion reaching distal margin of ventrite IV; width of the expansion of ventrite I, on ventrite III, approximately four times as wide as distance between clypeal teeth. Aedeagus with parameres subtriangular in lateral view, dorsal and ventral edges straight in lateral view (Fig 14F). Internal margins of paramera broadened on basal half in dorsal view (Fig 14F’). External margins of paramera broadened toward apex and the apex of paramera almost truncate in dorsal view (Fig 14F’). Apex of paramera flattened in ventral view. Medial area of endophallus with one endophallite (Fig 16K).

#### Composition

*Deltochilum komareki* Balthasar, 1939 and at least five undescribed species.

#### Geographic distribution

The species in this species-group are known to be distributed (Fig 29C), so far, in the following dominions and provinces (in parentheses): Parana (Atlantic, Parana Forest and Araucaria Forest), Chacoan (Cerrado, Chacoan and Pampean) and South Brazilian (Rondônia).

This species-group can be found in several localities in sympatry with the following species-groups (Table 11): *guyanense*, *irroratum*, *morbillosum* and *sextuberculatum*.

**Table 11:** Sympatry of the *komareki* species-group with other species-groups by province.

Species-group	Province(s)
*guyanense*	Cerrado (few localities)
*irroratum*	Atlantic, Parana Forest, Cerrado and Rondônia (a few localities)
*morbillosum*	Parana Forest, Araucaria Forest and Chacoan (a few localities)
*sextuberculatum*	Parana Forest, Cerrado and Rondônia (several localities)

### The *lindemannae* species-group

#### Diagnosis

This species-group shares the following combination of character states with the *aspericolle*, *femorale*, *genieri* species-groups and the *Deltochilum inesae* **sp. nov.** (*incertae sedis*): anterior margin of the clypeus, between clypeal teeth, concave and expanded posteriorly into triangular shape (Fig 5D); posterior edge of metafemur with two margins (Fig 10H and 10I); ventral surface of the protibia without carina or tubercles (Fig 9A–9C and 9J). However, it can be easily distinguished by the internal margin of hypomera that is strongly enlarged towards anterior angle (Fig 7C, arrows); it is regular, not enlarged towards anterior angle (Fig 7A, arrows) in *genieri* species-group. Can also be distinguished by the inter-ocular distance, which is under nine times the width of one eye, in *aspericolle* species-group the inter-ocular distance is over nine times width of one eye.

Otherwise, it can be easily separated from *D*. *inesae* **sp. nov.** (*incertae sedis*) by the pronotum, which have shiny points in *lindemannae* species-group and completely absent in *D*. *inesae* **sp. nov.** (*incertae sedis*). Also, males of *D*. *inesae* **sp. nov.** (*incertae sedis*) the posterior edge of mesofemur has an acute basal expansion, whereas the mesofemur in *lindemannae* species-group is regular, without expansion.

Moreover, it can be easily separated from *femorale* species-group by the body size 6.1–9.1mm in length, whereas it is 7.7–11.2mm in *lindemannae* species-group, as well as, by the inter-ocular distance is under nine times the width of one eye, in *femorale* species-group the inter-ocular distance is over nine times width of one eye. Otherwise, by their secondary sexual dimorphism, in which the posterior femur is regular bear or not setae (Fig 20A and 20B) in *lindemannae* species-group whereas it bears a broad medial serratulate expansion in *femorale* species-group (Fig 19G) (for further information see “Remarks” section of the *barbipes* and *genieri* species-groups).

#### Description

Small to medium-sized species, length 7.7–11.2mm, humeral width 4.8–7.8mm. Clypeal median emargination broadly U-shaped. Clypeal teeth separated by at least a basal width of a tooth. Anterior margin of the clypeus between, clypeal teeth, concave and expanded posteriorly into triangular shape (Fig 5D). Eyes large, inter-ocular distance six to eight times width of one eye. Internal margin of hypomera strongly enlarged towards anterior angle (Fig 7C, arrows). Tubercles at elytral apex on interstriae III, V-VII with all tubercles well developed or with III poorly developed. Interstriae VI-VII with basal tubercles (Fig 4B, see also Fig 23C and 23D, arrows). Striae inconspicuous (Fig 4B) or conspicuous, if conspicuous narrow, width third stria approximately 1/25th of the distance between striae II and III. Metaventrite with a very weak posterior excavation occupying fourth basal. Ventral surface of protibia without tubercles or carina (Fig 9A–9C and 9J). Posterior edge of metafemur with two margins (Fig 10H and 10I). **Male**. Protibial spur broad and foliaceus. Apex of mesotibia on ventral-internal margin with spatulate expansion (Fig 18C) or a denticle (Fig 18B). Internal margin of metatibia with small or large tubercles (see Fig 18E and 18G), and with long setae (see Fig 18H) or not. Metatrochanter modifier or not, if modified with setae. Metafemur modified or not, if modified with long setae on basal third (Fig 20A) or almost reaching the entire femur length (Fig 20B). Ventrite I expanded posteriorly (see Fig 21), expansion reaching from middle of ventrite IV to almost distal margin of ventrite V; width of the expansion of ventrite I, on ventrite III, from almost subequal to twice as wide as distance between clypeal teeth. Expansion basally with (see Fig 21I, arrow) or without orifice. Aedeagus with paramera subtriangular (Fig 14I), slender (Fig 14G) or lightly slender (Fig 14H) in lateral view. Most commonly slender, when slender the apex of paramera curving ventrally in lateral view (Fig 14G). If subtriangular or lightly slender, apex of paramera with short and thin apical-dorsal notch (Fig 14H’ and 14I’). Medial area of endophallus with one (Fig 17A) or two endophallites (Fig 16L). Sub-medial area of endophallus with raspules (Fig 16L) or large scales (Fig 17A).

#### Composition

*Deltochilum lindemannae* Balthasar, 1967, *D*. *bordoni* Halffter & Martínez, 1976 and at least five undescribed species.

#### Geographic distribution

The species in this species-group are known to be distributed (Fig 29D), so far, in the following dominions and provinces (in parentheses): Boreal Brazilian (Pantepui and Imerí) and South Brazilian (Ucayali and Rondônia).

This species-group can be found in a few localities in sympatry with the following species-groups (Table 12): *barbipes*, *gilli*, *granulatum* and *septemstriatum*.

**Table 12:** Sympatry of the *lindemannae* species-group with other species-groups by province.

Species-group	Province(s)
*barbipes*	Rondônia and Ucayali (a few localities)
*gilli*	Pantepui (few localities)
*granulatum*	Rondônia (one locality, Bolivia, Cochabamba, 124km E Cochabamba)
*septemstriatum*	Pantepui (one locality Venezuela, Bolivar, El Dorado)

#### Remarks

One species of *lindemannae* species-group which male does not have setae on posterior edge of metafemur can be confused with males of *aspericolle* species-group with a weak steep tapering on the posterior edge of metafemur, with *susanae* species-group or with specimens bearing a weak expansion on posterior edge of metafemur with *komareki* species-group. That species of *lindemannae* species-group can be distinguished from those specimens of the species within *susanae* and *komareki* species-groups by the posterior edge of metafemur, which has two margins (Fig 10H and 10I) in *lindemannae* species-group, whereas it bears a single margin, the dorsal, where the ventral surface of metafemur forms a declivity of approximately 45° on the posterior edge, in the other two species-groups (Fig 10E and 10F).

### The *morbillosum* species-group

#### Diagnosis

This species-group can be distinguished from all others species-groups (except from *bidentatum* species-group) by having only a carina on elytral base on the seventh interstria (Figs 4C and 23A and 23B, arrow) whereas, for the others, the elytral base have two carinae, one on the interstria VI and the other one on the interstria VII (Fig 23C and 23D, arrows). Can be separated from *bidentatum* species-group by having small not elevated shiny points mixed with punctures on the pronotal disc and on the interstriae (Fig 23B), that shiny points are large and elevated in *bidentatum* species-group (Fig 23A).

#### Description

Small species, length 9.5–10.6mm, humeral width 6.5–7.3mm. Clypeal median emargination broadly U-shaped. Clypeal teeth separated by at least a basal width of a tooth. Anterior margin of the clypeus between, clypeal teeth, concave, slightly expanded posteriorly, but not triangular in shape (Fig 5A–5C). Eyes medium-sized, inter-ocular distance approximately ten times width of one eye. Internal margin of hypomera enlarged towards anterior angle (Fig 7B, arrows). Tubercles at elytral apex on interstriae III-VII with all tubercles well developed. Interstria VII with basal carina (Figs 4C and 23B, arrow). Striae conspicuous and broad (Fig 4C), width of third stria approximately 1/15th of the distance between striae II and III. Metaventrite with a weak posterior excavation occupying fourth basal. Ventral surface of protibia with carina (Fig 9E and 9H). Posterior edge of metafemur with one margin, the dorsal; ventral surface of metafemur forming a decline of approximately 45° on posterior edge (Fig 10E and 10F). **Male**. Protibial spur broad and apically bifid (Fig 9E). Apex of mesotibia on ventral-internal margin with a small denticle (Fig 18B). Metafemur more curved than female. Ventrite I expanded posteriorly (See Fig 21), expansion from almost reaching distal margin ventrite II to reaching distal margin of ventrite III. Paramera subtriangular, with dorsal and ventral edges straight in lateral view (Fig 14J). Paramera with short and thin anteapical-dorsal notch. Ventral membranes between parameres sclerotised medially. Dorsal membranes of parameres lightly sclerotised medially. Apex of paramera acute in dorsal view. Medial area of endophallus with two endophallites (Fig 17B), right endophallite broader basally and apex bent forming a hook. Left endophallite two or three times smaller than right endophallite. Basal circular shape endophallite with ring very thin.

#### Composition

*Deltochilum morbillosum* Burmeister, 1848, *D*. *cristatum* Paulian, 1938 and at least five undescribed species.

#### Geographic distribution

**Fig 30:**
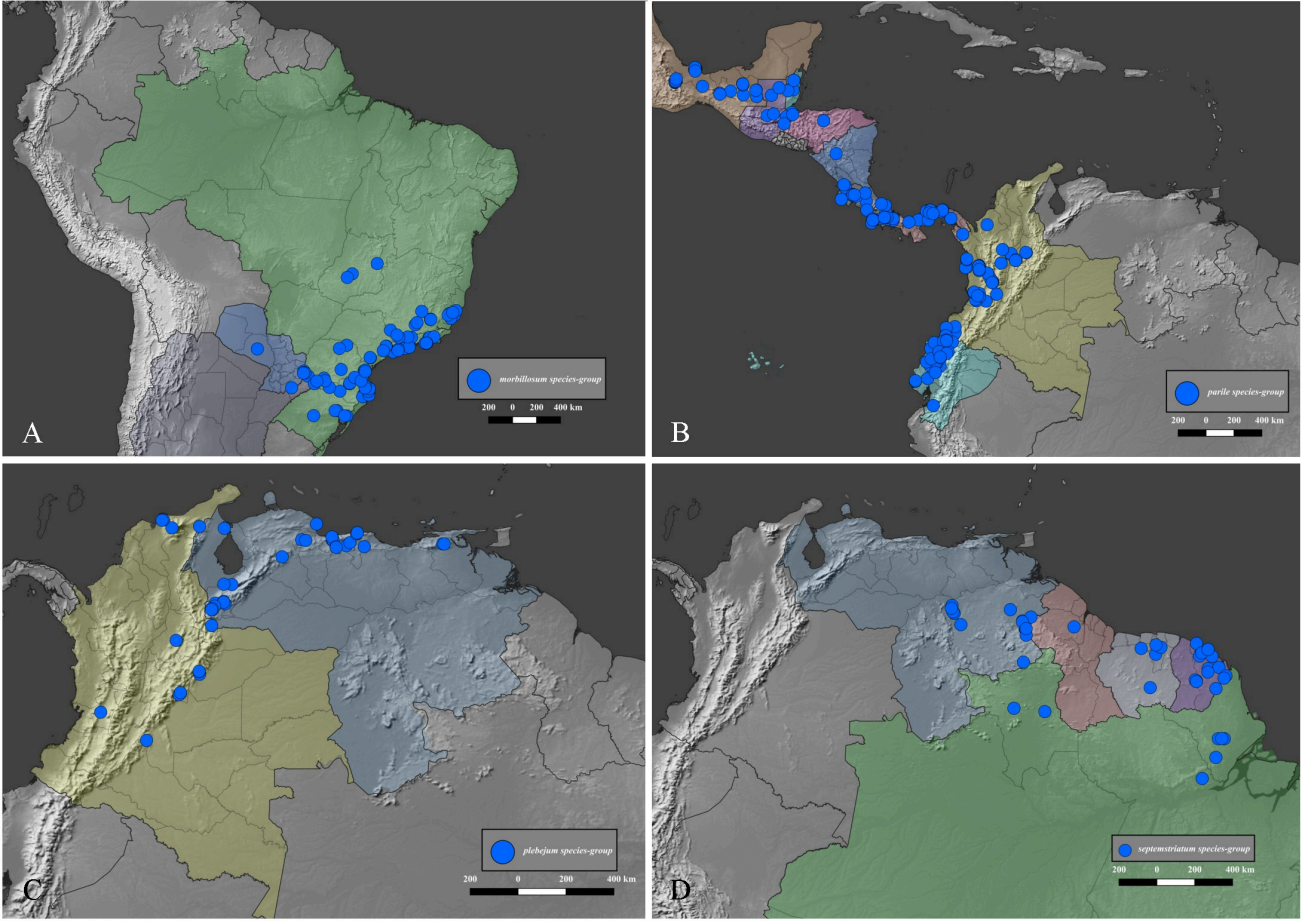
Distribution maps. (A) *morbillosum* species-group. (B) *parile* species-group. (C) *plebejum* species-group. (D) *septemstriatum* species-group.

The species in this species-group are known to be distributed (Fig 30A), so far, in the following dominions and provinces (in parentheses): Parana (Atlantic, Parana Forest and Araucaria Forest), Chacoan (Cerrado and Chacoan).

This species-group can be found in sympatry with *irroratum* species-group in Atlantic, Parana Forest (some localities) and with *komareki* species-group in Parana Forest, Araucaria Forest and Chacoan (a few localities).

### The *parile* species-group

#### Diagnosis

Most of the species of the *parile* species-group can be distinguished for all other known species of *Deltohyboma*, by the males. First ventrite is regular, not expanded posteriorly (Fig 21D) and the aedeagus bears dorsal paired sclerotised structures fused with the paramera (Fig 15A–15B’, arrows). However, in three undescribed species, the first ventrite is expanded posteriorly (see Fig 21) and the aedeagus has not dorsal paired sclerotised structures (Fig 15C); in those three species the aedeagus has the apex of paramera broadened and the apex formed by the paramera truncate in dorsal view (Fig 15C’), also, with a strong basal sinuation in lateral view (Fig 15C’, arrow); this shape of aedeagus is unique within *Deltohyboma*. However, in all species of this species-group the right endophallite is always triangular or sub-triangular in shape (for further information see “Remarks” section of the *aequinoctiale* species-group).

#### Description

Small to large species, length 7.5–14.5mm, humeral width 4.8-9mm. Clypeal median emargination broadly U-shaped. Clypeal teeth separated by at least a basal width of a tooth. Anterior margin of the clypeus, between clypeal teeth, concave, slightly expanded posteriorly, but not triangular in shape (Fig 5A–5C). Eyes large, inter-ocular distance eight to nine times width of one eye. Internal margin of hypomera not enlarged towards anterior angle (Fig 7A, arrows). Tubercles at elytral apex on interstriae III-VII with all tubercles well developed, tubercle on III approximately triangular in shape (Fig 24B and 24C, arrows) or transverse. Interstriae VI-VII with basal tubercles (Figs 4D, 4E and 23D, arrows). Striae inconspicuous (Fig 4D) or conspicuous (Fig 4E), if conspicuous very narrow, width of third stria approximately 1/40th of the distance between striae II and III. Metaventrite with a weak posterior excavation occupying third basal. Ventral surface of protibia with tubercles and/or carina (Fig 9E–9I). Posterior edge of metafemur with one margin, the dorsal; ventral surface of metafemur forming a decline of approximately 45°on posterior edge (Fig 10E and 10F). **Male**. Protibial spur broad and foliaceus. Ventral surface of protibia with tubercles and/or carina. Apex of mesotibia on ventral-internal margin with a small or large spatulate expansion (Fig 18C). Mesofemur modified or not, if modified with a sinuation on basal third, or a basal tubercle. Metafemur slightly more curved than female and in few species with small expansion. Internal margin of metatibia modified or not, if modified with large tubercles (see Fig 18E and 18G). Metatibia with spur insertion elongate (Figs 11B and 18E) or not (Figs 11A and 18F). Ventrite I expanded (See Fig 21) or not (Fig 21D) posteriorly, if expanded, expansion almost or reaching distal margin of ventrite V; width of the expansion of ventrite I, on ventrite III, from five to six as wide as distance between clypeal teeth. Aedeagus with paramera subtriangular (Fig 15A–15C), with ventral edge straight (Fig 15B) or concave in lateral view (Fig 15A and 15C). Apex of paramera with dense setae (Fig 15A–15C). Paramera with (Fig 15A’ and 15B’, arrows) or without (Fig 15C’) dorsal paired sclerotised structures on internal edge, if present attached to the internal edge of paramera in different parts, can be attached on apical 2/3 to almost on the apex; apex of paramera rounded in dorsal view. If without paired sclerotised structures, apex of paramera broadened and truncate in dorsal view (Fig 15C’) and with a strong basal sinuation in lateral view (Fig 15C, arrow). Medial area of endophallus with following variation: 1) three endophallites (Fig 17C), 2) two endophallites and one raspule (Fig 17D and 17E, arrow showing the raspule), 3) one endophallite and one raspule. However, the right endophallite always with triangular or sub-triangular in shape. Sub-medial area of endophallus without (Fig 17C and 17D) or with a large raspule (Fig 17E).

#### Composition

*Deltochilum parile* Bates, 1887, *D*. *pseudoparile* Paulian, 1938, *D*. *violetae* (Martínez, 1991) and at least 20 undescribed species.

#### Geographic distribution

The species in this species-group are known to be distributed (Fig 30B), so far, in the following dominions and provinces (in parentheses): Pacific (Magdalena, Cauca, Western Ecuador, Pacific, Guatuso-Talamanca and Puntarenas-Chiriquí), and Mesoamerican (Mosquito, Pacific Lowlands and Veracruzan). As well as, in the Mexican transition zone, Chiapas Highlands and Sierra Madre del Sur provinces. This is the only species-group with species distributed in Central America.

This species-group can be found in a few localities in sympatry with *plebejum* species group in the Magdalena province (one locality, Colombia, Santander, El Carmen de Chucurí, Vereda La Belleza) and with *aequinoctiale* species-group in the Magdalena and Cauca provinces (several localities).

### The *plebejum* species-group

#### Diagnosis

This species-group shares the following combination of character states with the *aequinoctiale*, *bidentatum*, *irroratum*, *morbillosum*, *parile* and *sextuberculatum* species-groups: anterior margin of the clypeus, between clypeal teeth, concave, slightly expanded, but not triangular in shape (Fig 5A–5C); posterior edge of metafemur with one margin, where the ventral surface of metafemur forms a decline of approximately 45° on posterior edge (Fig 10E and 10F); ventral surface of protibia with carina or tubercles (Fig 9E–9I). However, it can be easily separated from *bidentatum* and *morbillosum* species-groups by the humeral region, which bears one carina (Figs 3F, 4C and 23B) (interstria VII), whereas there are two carinae (Fig 4F–4G, see also Fig 23C and 23D) (interstriae VI and VII) in *plebejum* species-group (for further information see “Remarks” section of the *aequinoctiale* species-group).

Otherwise, from *sextuberculatum* species-group can be distinguished by the broad striae (Fig 4I and 22E), width of third stria, in species with the narrowest striae, 1/15th of the distance between striae II and III, whereas the striae are narrow (Figs 4F, 4G and 22F), width of third stria, in species with the broadest striae, 1/20th of the distance between striae II and III in *plebejum* species-group, as well as via distribution.

Males of these two species groups can also be separated by their secondary sexual dimorphism, in which the metafemur has a medial denticle in *sextuberculatum* species-group (Fig 20D) whereas it does not have this modification, it is only more curved than females, in *plebejum* species-group. Also, from *irroratum* species-group can be distinguished by the metatarsomeres II and III that each is almost as long as broad (Fig 11A), whereas each is longer than broad (Fig 11B) in *plebejum* species-group.

#### Description

Medium-sized species, length 8.4–12.8mm, humeral width 5.3–8.7mm. Clypeal median emargination broadly U-shaped. Clypeal teeth separated by approximately twice basal width of a tooth. Anterior margin of the clypeus, between clypeal teeth, concave, slightly expanded posteriorly, but not triangular in shape (Fig 5B and 5C). Eyes large, inter-ocular distance seven to nine times width of one eye. Internal margin of hypomera not enlarged towards anterior angle (Fig 7A, arrows). Tubercles at elytral apex on interstriae with following variations: 1) III, V-VII with all tubercles well developed. 2) III-VII with all tubercles well developed or with IV poorly developed. 3) II-VII with II only slightly elevated. But in all those variations the tubercle on interstria III is elongate (Fig 24D–24F, arrows). Interstriae VI-VII with basal tubercles (Fig 4F and 4G, see also Fig 23C and 23D, arrows). Striae almost inconspicuous (Fig 4F) or narrow (Figs 4G and 22F), width of third stria, in species with the broadest striae, 1/20th of the distance between striae II and III. Metaventrite with a weak posterior excavation, occupying approximately metaventral basal fourth. Ventral surface of protibia with tubercles or carina (Fig 9E–9I). Posterior edge of metafemur with one margin, the dorsal, ventral surface of metafemur forming a decline of approximately 45° on posterior edge (Fig 10E and 10F). **Male**. Mesofemur lacking or with a tubercle on basal third. Apex of mesotibia on ventral-internal margin with a large spatulate expansion (Fig 18C). Metafemur more curved than female. Internal margin of metatibia with large tubercles (see Fig 18E and 18G) or strong carina (see Fig 18F). Metatibia with spur insertion elongate (Figs 11B and 18E) or not (Figs 11A and 18F) and spur articulated or fused (Fig 18E, arrow); if the spur insertion elongate, spur reaching second tarsomere to almost as elongate as tarsus. Ventrite I expanded posteriorly (See Fig 21), expansion reaching from middle of ventrite III to middle of ventrite V; width of the expansion of ventrite I, on ventrite III, from slightly wider to seven times as wide as distance between clypeal teeth. Aedeagus with apex of paramera without setae and with a large or small lateral-ventral denticle in lateral view (Fig 15D and 15E). Medial area of endophallus one (Fig 17G) or two endophallites (Fig 17F), if with two, the left endophallite six times smaller than right endophallite (Fig 17F).

#### Composition

*Deltochilum plebejum* Balthasar, 1939, *D*. *abdominalis* Martínez, 1947 and at least seven undescribed species.

#### Geographic distribution

The species in this species-group are known to be distributed (Fig 30C), so far, in the Pacific dominion: Sabana, Venezuelan, Guajira, Magdalena and Cauca provinces; as well as, in the South American transition zone, Paramo province.

This species-group can be found in a few localities in sympatry with the following species-groups (Table 13): *aequinoctiale*, *genieri* and *parile*.

**Table 13:** Sympatry of the *plebejum* species-group with other species-groups by province.

Species-group	Province(s)
*aequinoctiale*	Sabana and Paramo (few localities)
*genieri*	Sabana and Paramo (few localities)
*parile*	Magdalena (one locality, Colombia, Santander, El Carmen de Chucurí, Vereda La Belleza)

### The *septemstriatum* species-group

#### Diagnosis

For all species known of *Deltohyboma*, species in the *septemstriatum* species-group can be distinguished because they are the smallest species (less than 8mm in length) with the following combinations of character states: striae I-VII conspicuous and broad (Figs 4H and 22D), width of each stria approximately 1/10th of the width of each interstria; anterior margin of the clypeus, between clypeal teeth, concave and regular (Fig 5A), even not slightly expanded; posterior edge of metafemur with two margins (Fig 10H and 10I). Also, the males can be distinguished by the unique modification of the posterior femur, it bears a broad medial expansion (Fig 20C) (for further information see “Remarks” section of the *aspericolle* species-group).

#### Description

Small species, length 6.1–7.3mm, humeral width 3.9–4.5mm. Clypeal median emargination broadly U-shaped. Clypeal teeth separated approximately by twice basal width of a tooth. Anterior margin of the clypeus, between clypeal teeth, concave and regular, not expanded posteriorly (Fig 5A). Eyes medium-sized, inter-ocular distance ten to 12 times width of one eye. Internal margin of hypomera strongly enlarged towards anterior angle (Fig 7C, arrows). Tubercles at elytral apex on interstriae V-VII, some cases with V poorly developed. Interstriae VI-VII with basal tubercles (Fig 4H, see also Fig 23C and 23D, arrows) Striae I-VII conspicuous and broad (Figs 4H and 22D), width of each stria approximately 1/10th of the width of each interstria. Metaventrite with a weak posterior excavation, occupying approximately metaventral basal fourth. Ventral surface of the protibia without tubercles or carina (Fig 9A–9C and 9J). Posterior edge of metafemur with two margins (Fig 10H and 10I). **Male**. Protibial spur broad and foliaceus. Apex of mesotibia on ventral-internal margin with a small denticle (Fig 18B, arrow). Internal margin of metatibia with large tubercles (see Fig 18E–18H). Posterior-ventral margin of metafemur with a broad medial expansion (Fig 20C). Ventrite I expanded posteriorly (see Fig 21); width of expansion of ventrite I, on ventrite III, from slightly narrower to slightly wider than distance between clypeal teeth; expansion surpassing the middle of ventrite V, but not almost reaching distal margin of that ventrite or almost reaching distal margin of ventrite V. Paramera subtriangular, with dorsal and ventral edges straight in lateral view (Fig 15F). Apex of paramera slightly emarginate in dorsal view (Fig 15F’). Medial area of endophallus with one endophallite comma “,”-shaped (Fig 17H).

#### Composition

*Deltochilum septemstriatum* Paulian, 1938 and at least four undescribed species.

#### Geographic distribution

The species in this species-group are known to be distributed (Fig 30D), so far, in the Boreal Brazilian dominion, Guianan Lowlands, Roraima and Pantepui provinces.

This species-group can be found in several localities in sympatry with the following species-groups (Table 14): *aspericolle*, *gilli*, *granulatum*, *guyanense*, *lindemannae* and *submetallicum*.

**Table 14:** Sympatry of the *septemstriatum* species-group with other species-groups by province.

Species-group	Province(s)
*aspericolle*	Guianan Lowlands (some localities)
*gilli*	Guianan Lowlands (one locality Guyana, Cuyuni-mazaruni, Takutu Mountain)
*granulatum*	Roraima (one locality, Brazil, Pará Monte Dourado)
*guyanense*	Guianan Lowlands, Roraima (some localities)
*lindemannae*	Pantepui (one locality Venezuela, Bolivar, El Dorado)
*submetallicum*	Guianan Lowlands, Roraima (some localities)

### The *sextuberculatum* species-group

#### Diagnosis

For all species known of *Deltohyboma*, species in the *sextuberculatum* species-group can be distinguished because they are the smallest species (less than 10mm in length) with the following combinations of character states: striae I-VII conspicuous and wide (Figs 4I and 22E), width of third stria, in species with the narrowest striae, 1/15th of the distance between striae II and III; anterior margin of the clypeus, between clypeal teeth, concave and slightly expanded (Fig 5B and 5C); posterior edge of metafemur with one margin, the dorsal, where the ventral surface of metafemur forming a decline of approximately 45° on posterior edge (Fig 10E and 10F). Also, the males can be distinguished by the unique modification of the posterior femur, it bears a medial denticle (Fig 20D).

#### Description

Small species, length 9–9.8m, humeral width 5.7–6.9mm. Clypeal median emargination broadly U-shaped. Clypeal teeth separated by at least twice basal width of a tooth. Anterior margin of the clypeus, between clypeal teeth, concave, slightly expanded posteriorly, but not triangular in shape (Fig 5B and 5C). Eyes small, inter-ocular distance 12 to 20 times width of one eye. Internal margin of hypomera enlarged towards anterior angle (Fig 7B, arrows). Tubercles at elytral apex on interstriae II-VII with all tubercles well developed or II few developed, or III-VII with all tubercles well developed. Interstriae VI-VII with basal tubercles (Fig 4I, see also Fig 23C and 23D, arrows). Striae conspicuous and broad (Fig 4I and 22E), width of third stria, in species with the narrowest striae, 1/15th of the distance between striae II and III. Metaventrite with a weak posterior excavation occupying fourth basal. Ventral surface of protibia with tubercles or carina (Fig 9E–9I). Posterior edge of metafemur with one margin, the dorsal; ventral surface of metafemur forming a decline of approximately 45° on posterior edge (Fig 10E and 10F). **Male**. Protibial spur broad and foliaceus. Ventral surface of protibia with tubercles. Apex of mesotibia on ventral-internal margin with large spatulate expansion (Fig 18C). Metafemur on posterior edge of with a medial denticle (Fig 20D). Internal margin of metatibia with large tubercles (see Fig 18E and 18G). Ventrite I expanded posteriorly (see Fig 21), expansion almost reaching distal margin ventrite V. Paramera subtriangular, with dorsal and ventral edges straight in lateral view (Fig 15G). Paramera with internal edge broadened on basal half in dorsal view (Fig 15G’). Apex formed by the paramera acute in dorsal view (Fig 15G’). Medial area of endophallus with two endophallites (Fig 17I), right endophallite with triangular shape and left endophallite very small. Basal circular shape endophallite with ring very thin and handle broadened medially. Sub-medial area of endophallus with large scales.

#### Composition

*Deltochilum sextuberculatum* Bates, 1870 and at least ten undescribed species.

#### Geographic distribution

**Fig 31:**
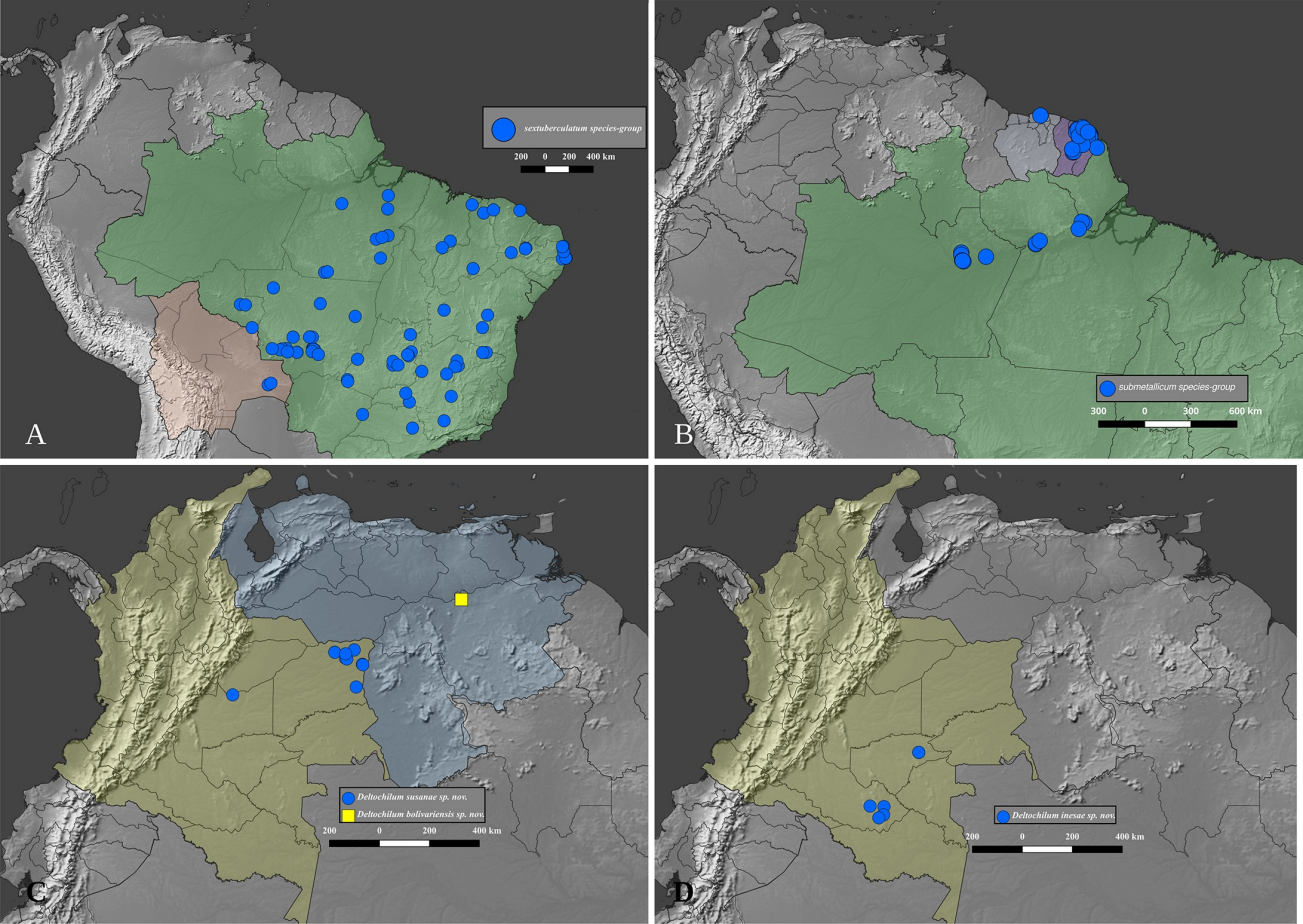
Distribution maps. (A) *sextuberculatum* species-group. (B) *submetallicum* species-group. (C) *susanae* species-group, blue circles = *Deltochilum susanae* **sp. nov.**, yellow square = *Deltochilum bolivariensis* **sp. nov.** (D) *Deltochilum inesae* **sp. nov.** (*incertae sedis*).

The species in this species-group are known to be distributed (Fig 31A), so far, in the following dominions and provinces (in parentheses): Parana (Atlantic and Parana Forest), Chacoan (Caatinga and Cerrado), Borela Brazilian (Pará), South-eastern Amazonian (Xingu-Tapajós) and South Brazilian (Madeira and Rondônia).

This species-group can be found in several localities in sympatry with the following species-groups (Table 15): *barbipes*, *granulatum*, *guyanense*, *irroratum* and *komareki*.

**Table 15:** Sympatry of the *sextuberculatum* species-group with other species-groups by province.

Species-group	Province(s)
*barbipes*	Xingu-Tapajós and Madeira (few localities)
*granulatum*	Pará and Xingu-Tapajós (a few localities)v
*guyanense*	Pará, Xingu-Tapajós and Cerrado (few localities)
*irroratum*	Atlantic, Cerrado and Rondônia (few localities)
*komareki*	Parana Forest, Cerrado and Rondônia (several localities)

### The *submetallicum* species-group

#### Diagnosis

For all species known of *Deltohyboma*, males in the *submetallicum* species-group can be distinguished by the metafemur, in which the posterior edge has one margin, the posterior-dorsal, the ventral surface of metafemur is continuous to that margin (Fig 10B and 10C), additionally bearing a basal denticle (Fig 20E and 20E’) (for further information see “Remarks” section of the *barbipes* species-group).

#### Description

Medium-sized to large species, length 11.7-13mm, humeral width 7.5–8.1mm. Clypeal median emargination broadly U-shaped. Clypeal teeth separated approximately by 2.5 times basal width of a tooth. Anterior margin of the clypeus, between clypeal teeth, concave and expanded posteriorly into triangular shape (Fig 5D). Eyes large, inter-ocular distance eight or nine times width of one eye. Internal margin of hypomera strongly enlarged towards anterior angle (Fig 7C, arrows). Tubercles at elytral apex on interstriae with following variations: 1) III, V-VII with all well-developed; 2) III, V-VII with III poorly developed; 3) V-VII with V poorly developed. Interstriae VI-VII with basal tubercles (Fig 4J, see also Fig 23C and 23D, arrows). Striae I-VII conspicuous and narrow (Fig 4J), width of each stria approximately 1/28th of the width of each interstria. Ventral surface of the protibia without tubercles or carina (Fig 9A–9C and 9J). Posterior edge of metafemur with one margin, the dorsal; ventral surface of metafemur continuous to the dorsal margin (Fig 10B and 10C). **Male**. Protibial spur broad and foliaceus. Apex of mesotibia on ventral-internal margin with a large spatulate expansion (Fig 18C). Internal margin of metatibia with large tubercles (see Fig 18E, 18G and 18H). Metafemur with a denticle on basal third of posterior margin (Fig 20E and 20E’). Ventrite I expanded posteriorly (see Fig 21), expansion reaching or surpassing distal margin of ventrite IV; width of expansion on ventrite III variable, subequal to wider than distance between clypeal teeth. Paramera subtriangular, with dorsal and ventral edges straight in lateral view (Fig 15H). Paramera with short and thin anteapical-dorsal notch (Fig 15H’). Apex formed by the paramera rounded in dorsal view (Fig 15H’). Medial area of endophallus with one endophallite (Fig 17J). Basal circular shape endophallite with the ring very small and thin, poorly sclerotised (Fig 17J’). Basal part of plate-shape endophallite “boot”-shaped (Fig 17J, arrow).

#### Composition

*Deltochilum submetallicum* (Castelnau, 1840), *D*. *diringshofeni* Pereira & Martínez, 1956 and two undescribed species.

#### Geographic distribution

The species in this species-group are known to be distributed (Fig 31B), so far, in the Boreal Brazilian, Guianan Lowlands and Roraima provinces.

This species-group can be found in a few localities in sympatry with the following species-groups (Table 16): *aspericolle*, *granulatum*, *guyanense* and *septemstriatum* species-groups.

**Table 16:** Sympatry of the *submetallicum* species-group with other species-groups by province.

Species-group	Province(s)
*aspericolle*	Guianan Lowlands and Roraima (some localities)
*granulatum*	Roraima (few localities)
*guyanense*	Guianan Lowlands and Roraima (some localities)
*septemstriatum*	Guianan Lowlands and Roraima (some localities)

### The *susanae* species-group

#### Diagnosis

For all species known of *Deltohyboma*, species in the *susanae* species-group can be distinguished because they are the smallest species (less than 10mm in length) with striae conspicuous as well as with the anterior margin of the clypeus, between clypeal teeth, concave and expanded posteriorly into triangular shape (Fig 5D) (for further information see “Remarks” section of the *aspericolle* species-group).

#### Description

Small-sized species, length 7.4–9.4mm, humeral width 4.6–6.2mm. Clypeal median emargination broadly U-shaped. Clypeal median emargination broadly U-shaped. Clypeal teeth separated by two or 2.5 times basal width of a tooth. Anterior margin of the clypeus, between clypeal teeth, concave and expanded posteriorly into triangular shape (Fig 5D). Eyes medium-sized, inter-ocular distance nine to 11 times eye width. Internal margin of hypomera strongly enlarged towards anterior angle (Fig 7C, arrows). Tubercles at elytral apex on interstriae III, V-VII, with III and VII few developed. Interstriae VI and VII with basal tubercles (Fig 4K, see also Fig 23C and 23D, arrows). Carina of ninth interstria almost reaching middle of elytral length. Striae I-VIII conspicuous and wide (Fig 4K), width third stria approximately 1/21th of distance between striae II and III. Metaventrite with a weak posterior excavation, occupying approximately metaventral basal fourth. Ventral surface of the protibia with carina (Fig 9E and 9H). Posterior edge of metafemur with one margin, the dorsal; ventral surface of metafemur forming a decline of approximately 45° on posterior-ventral edge (Fig 10E and 10F). **Male**. Protibial spur broad. Apex of mesotibia on ventral-internal margin with a large spatulate expansion (Fig 18C, arrow). Internal margin of metatibia with large tubercles (see Fig 18E, 18G and 18H). Ventrite I expanded posteriorly (see Fig 21), expansion reaching or surpassing middle of ventrite V. Width of expansion on ventrite III, three or four times as wide as distance between clypeal teeth. Paramera subtriangular, dorsal and ventral edges straight in lateral view (Fig 15I). Apex formed by the paramera truncate in dorsal view (Fig 15I’). Paramera with short and thin apical-dorsal notch (Fig 15I’). Medial area of endophallus with one endophallite (Fig 17K) comma “,”-shaped, basally broadened and bent and apically bent forming a hook.

#### Composition

*Deltochilum susanae* **sp. nov**. and *D*. *bolivariensis* **sp. nov.**

#### Geographic distribution

The species in this species-group are known to be distributed (Fig 31C), so far, in the Boreal Brazilian dominion, Imerí province and Pacific dominion, Sabana province. This species-group may be found in sympatry in Imerí province with *guyanense* species-group. Species of these species-groups were collected in Colombia, Vichada, Cumaribo, Selva de Matavén, but not in the same locality.

#### *Deltochilum susanae* sp. Nov

urn:lsid:zoobank.org:act:797FAD1C-AF29-4A40-80FC-0D5238306E7E

(Figs 31C blue circles, 32A, 32B, 32E, 32F and 32I)

*Deltochilum* sp. 12H [71]⁠: 230

### Material examined

#### Holotype

**♂**, **COLOMBIA: Vichada:** Cumaribo, Selva de Matavén. Sabana, 04°31'56"N, 68°05'28"W, 240m, 2007.iii.17-19, Franco L.E., Trampa de caída con excremento humano T19 (IAvH) [IAvH-E-90829]. [aedeagus and endophallus extracted].

#### Paratypes

**COLOMBIA: Vichada:** Cumaribo, Ctgo. Santa Rita, Centro de visitas. Parque Nacional Natural El Tuparro, 5°20'N, 67°51'W, 450m, **♀** (IAvH), Cumaribo, Ctgo. Santa Rita. PNN El Tuparro. Sabana, 5°21'N, 67°52'W, 135m, **♀**, 2004.ii.3-5, Quintero I. & González E., T. Exc. H. (IAvH), Cumaribo, Selva de Matavén. Sabana, 04°31'56"N, 68°05'28"W, 240m, **♀**, 2007.iii.17-19, Franco L.E., Trampa de caída con excremento humano T19 (IAvH), **♀**, 2007.iii.17-19, Franco L.E., Trampa de caída con excremento humano T5 (IAvH), 2**♂♂**, 2007.iii.17-19, Franco L.E., Trampa de caída con excremento humano T7 (IAvH), Cumaribo. PNN El Tuparro. B. cerro, 5°20'N, 67°52'W, 150m, 2**♀♀**, 2002.ix.17, Quintero I., Pitfall excremento humano (IAvH), La Primavera, Ventana La Florida. Morichal, 05°47'4.8"N, 68°51'50.1"W, 66m, 3**♂♂**, 2016.v.28, Martínez D.E., T. Exc. H. (48h) E2-V1-T26 (IAvH), **♀**, 2016.v.29, Martínez D.E., T. Exc. H. (72h) E2-V1-T26 (IAvH), La Primavera, Ventana La Florida. Morichal, 05°46'50.7"N, 68°51'50.3"W, 73m, **♂**, 2016.v.29, Martínez D.E., T. Exc. H. (72h) E2-V1-T4 (IAvH), Puerto Carreño, Mi Familia. Borde Morichal Sabana, 5°51'58.9"N, 68°9'42.5"W, 72m, **♂**, 2016.1.19–20, Medina C. & Castro C., Pitfall (IAvH), Puerto Carreño, Mi Familia. Morichal, 5°52'0.9"N, 68°9'43.9"W, 66m, **♂**, 2016.1.19–20, Medina C. & Castro C., Pitfall (IAvH), **♂**, 2016.1.19–21, Medina C. & Castro C., Pitfall (IAvH), Puerto Carreño, Rampa Vieja. Sabana sustrato rocoso, 05°43'44.3"N, 68°28'25.4"W, 68m, 3**♀♀**, 2016.vi.3, Martínez D.E., T. Exc. H. (48h) E2-V2-T18 (IAvH), Puerto Carreño, Vereda La Esmeralda, Finca El Tomo. Bosque de Tierra Firme, 05°34'24"N, 68°28'51"W, 81m, **♀**, 2017.iv.6, Lopera A. & Cárdenas J., T. Exc. H. TT8-24 (IAvH), Puerto Carreño, Vereda La Esmeralda, Finca El Tomo. Morichal, 05°33'41"N, 68°26'45"W, 86m, **♀**, 2017.iv.3, Lopera A. & Cárdenas J., T. Exc. H. MT9-48 (IAvH), Puerto Carreño, Vereda La Esmeralda, Finca El Tomo. Morichal, 05°33'43"N, 68°26'52"W, 87m, **♀**, 2017.iv.4, Lopera A. & Cárdenas J., T. Exc. H. MT5-72 (IAvH), Puerto Carreño, Vereda La Esmeralda, Finca El Tomo. Sabana no inundable, 05°32'28"N, 68°25'43"W, 70m, **♂**, 2017.iv.2, Lopera A. & Cárdenas J., T. Exc. H. ST8-24 (IAvH), Puerto Carreño, Vereda La Esmeralda, Finca El Tomo. Sabana no inundable, 05°32'31"N, 68°25'41"W, 71m, **♂**, 2017.iv.4, Lopera A. & Cárdenas J., T. Exc. H. ST2-72 (IAvH).

#### Non-type material

**COLOMBIA: Meta:** Cafam Llanos Remolino, [4°14'56.81"N], [72°32'29.91"W], 200m, **♀**, 1-2.iv.1996, S. Amézquita coll. (BDGC).

#### Diagnosis

**Fig 32:**
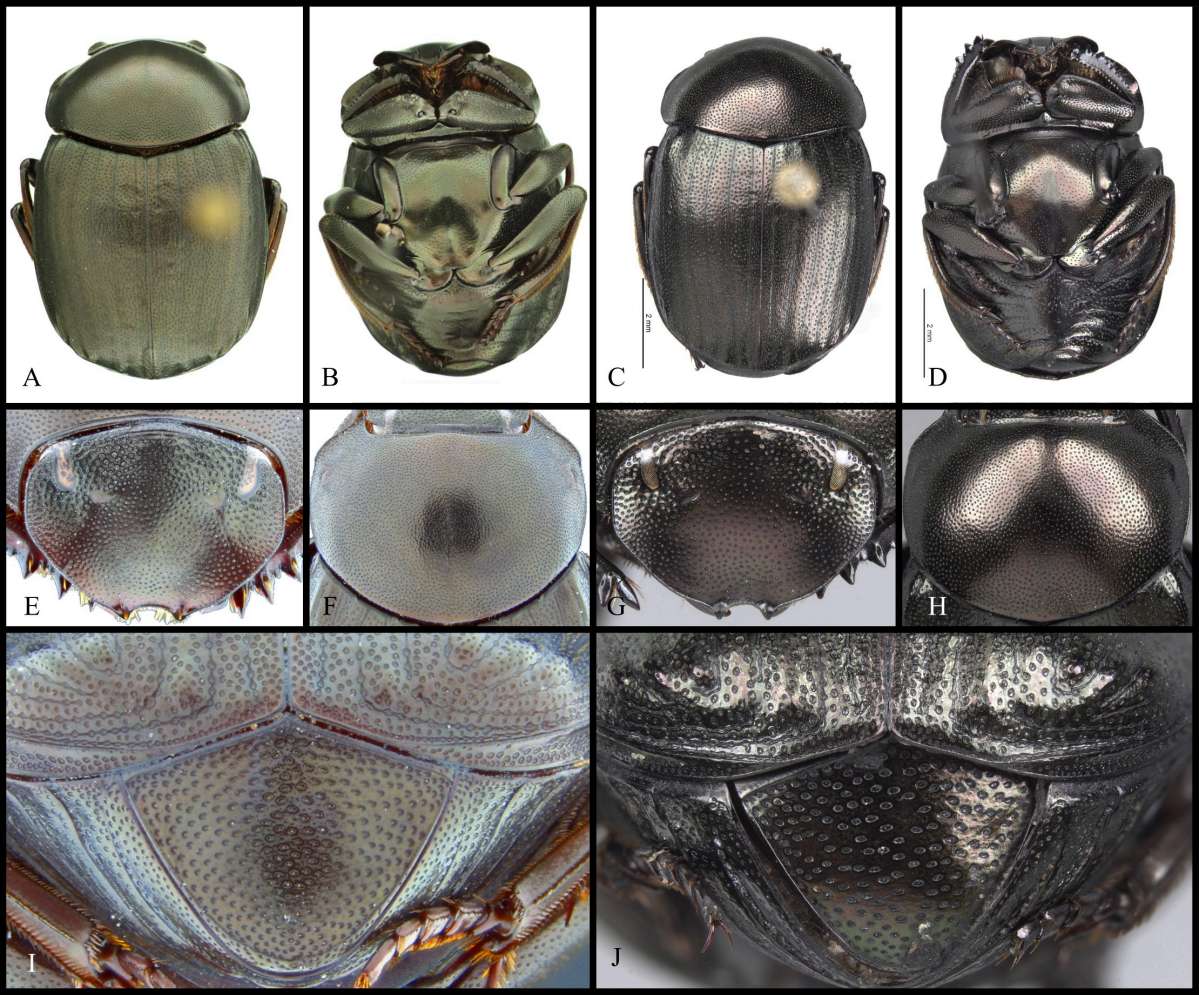
External morphology of the *susanae* species-group. Holotype *Deltochilum susanae* **sp. nov.** (A-B, E-F, I), Holotype *Deltochilum bolivariensis* **sp. nov.** (C-D, G-H, J). (A, C) habitus. (B, D) ventral view. (E, G) head. (F, H) pronotum. (I-J) caudal view.

The pronotal disc with irregular shiny points mixed with the punctures (Fig 32F), the smallest and densest punctures on the pygidium (Fig 32I) and the lateral punctures of the pygidium not fully closed, with horse-shoe shape, distinguish this species (Fig 32I).

#### Description

Holotype male, length 9.4mm, humeral width 6.2mm. Green with copper reflections dorsally and ventrally (Fig 32A and 32B). **Head** (Fig 32E). Dorsal inter-ocular distance approximately nine times width of one eye. Clypeal teeth separated approximately by 2.5 times basal width of a tooth. Edge thickness between clypeal teeth, the triangular expansion, three times broader than edge between genal suture and tooth. Front punctures separated by one or two distances between internal and external margin of each puncture. Disc puncture separated by one diameter of each puncture. Front punctures 1.5 times larger than disc punctures. **Pronotum** (Fig 32F). With irregular shiny points mixed with the punctures. Disc irregular shiny points subequal to disc punctures. Disc punctures separated by one or less than one diameter of each puncture. Basal punctures separated by two or three distances between internal and external margin of each puncture. Medial-lateral angle rounded, but with a slightly projection. **Elytra** (Fig 32A). Punctures of third interstria on disc occupying about 1/9th of the distance between striae II and III. **Abdomen** (Fig 32B). Width of expansion of ventrite I, on ventrite III, three times as wide as distance between clypeal teeth; expansion surpassing middle of ventrite V. **Pygidium** (Fig 32I). Discal punctures occupying approximately 1/29th the width on middle of pygidium, and separated by two distances between internal and external margin of each puncture. Lateral punctures not fully closed, with horse-shoe shape. **Genitalia**. As described in the *susanae* species-group (Figs 15I and 17K).

#### Remarks

Apart from the sexual dimorphism, little variation was observed. Only a few specimens are slightly larger or slightly smaller than the holotype. A female from Colombia Meta (BDGC) has not been included in the type series because more material is required to confirm the identification.

#### Etymology

A patronym, noun in the genitive case, after Susana Cruz, great friend of the first author, who sadly passed away, too young, in 2016.

#### Known distribution (Fig 31C, blue circles)

COLOMBIA. **Vichada**: Cumaribo, Selva de Matavén. Puerto Carreño. La Primavera.

#### *Deltochilum bolivariensis* sp. Nov

urn:lsid:zoobank.org:act:4696EB42-FA1F-4F41-9819-6F8A849B26F9

(Figs 31C yellow square, 32C-32D, 32G-32H and 32J)

### Material examined

#### Holotype

**♂**, **VENEZUELA: Bolívar:** Puente Cocuizas, 70 km Ciudad Bolivar. forested ravine in woodland, [7°41'35"N], [64°0'18"W], 19-3.vi-viii.1987, S. & J. Peck coll., flight interception trap (CMNC) [WSD00041151]. [aedeagus and endophallus extracted].

#### Paratype

**♀ VENEZUELA: Bolívar:** Puente Cocuizas, 70 km Ciudad Bolivar. forested ravine in woodland, [7°41'35"N], [64°0'18"W], 19-3.vi-viii.1987, S. & J. Peck coll., flight interception trap (CMNC).

#### Diagnosis

The pronotal disc without irregular shiny points mixed with the punctures (Fig 32H), the largest and most dispersed punctures on the pygidium (Fig 32J) and the lateral punctures of the pygidium fully closed distinguish this species (Fig 32J).

#### Description

Holotype male, length 7.4mm, humeral width 4.6mm. Copper with green reflections dorsally and ventrally (Fig 32C and 32D) **Head** (Fig 32G). Dorsal inter-ocular distance approximately 11 times width of one eye. Clypeal teeth separated approximately by twice basal width of a tooth. Edge thickness between clypeal teeth, the triangular expansion, four times broader than edge between genal suture and tooth. Front punctures separated by two or more than two distances between internal and external margin of each puncture. Disc puncture separated by one or more than one diameter of each puncture. Front punctures twice larger than disc punctures. **Pronotum** (Fig 32H). Without irregular shiny points mixed with the punctures. Disc punctures separated by one or more than one diameter of each puncture. Basal punctures separated by one distance between internal and external margin of each puncture. Medial-lateral angle rounded. **Elytra** (Fig 32C)

Punctures of third interstria on disc occupying about 1/12th of the distance between striae II and III. **Abdomen** (Fig 32D). Width of expansion of ventrite I, on ventrite III, four times as wide as distance between clypeal teeth; expansion reaching middle of ventrite V. **Pygidium** (Fig 32J). Discal punctures occupying approximately 1/20th the width on middle of pygidium, and separated by more than two distances between internal and external margin of each puncture. Lateral punctures fully closed. **Genitalia**. As described in *susanae* species-group (Figs 15I and 17K).

#### Remarks

The paratype differs from the holotype by the sexual dimorphism.

#### Etymology

Combination of Bolívar + -*ensis* “of or from a place” as reference to the state of Bolívar and Ciudad Bolívar, state and city (70 km from that city) where all the specimens known of this species were collected.

#### Known distribution (Fig 31C, yellow square)

VENEZUELA. **Bolívar**: Puente Cocuizas, 70 km Ciudad Bolívar.

#### *Deltochilum inesae* sp. nov. (*incertae sedis*)

urn:lsid:zoobank.org:act:907C1D9B-11F5-4E03-8EB1-6E9B75F95F1A

(Figs 31D blue circles, 4L, 15J, 17L, 20F and 33)

### Material examined

#### Holotype

**♂**, **COLOMBIA: Caquetá:** Puerto Solano, Río Cuñare-Amú. PNN La Serranía de Chiribiquete. Transición Bosque inundable, de tierra firme y arenoso, 0°13'25.6"N, 72°26'12.8"W, 250m, 2001.ii.24, González E. & Ospina M., Trampa caída con pescado (IAvH) [IAvH-E-13344].

#### Paratypes

**COLOMBIA: Caquetá:** Puerto Solano, Río Mesay. PNN La Serranía de Chiribiquete. Bosque de Tierra Firme, 0°14'32"N, 72°56'15"W, 250m, **♀**, **♂**, 2000.ii.4, Quevedo F., Trampa excremento humano (IAvH), **♀**, **♂**, 2000.ii.4, Quevedo F., Trampa Interceptación al vuelo (IAvH), Puerto Solano, Río Mesay. PNN La Serranía de Chiribiquete. Bosque Inundable, 0°14'24"N, 72°56'2"W, **♀**, 2000.i.25, Quevedo F., Trampa excremento humano (IAvH), Puerto Solano, Río Mesay. PNN La Serranía de Chiribiquete. Transición Bosque Tierra firme Bosque Inundable, 0°14'54"N, 72°56'5"W, 250m, **♂**, 2000.i.28, Quevedo F., Trampa excremento humano (IAvH), Puerto Solano, Taquita, río Mesay. PNN La Serranía de Chiribiquete. Bosque de Tierra Firme, 0°14'32"N, 72°56'15"W, 250m, **♀**, 2000.ii.4, Quevedo F., T. Exc. H. 93 (IAvH), Puerto Solano, Taquita, río Mesay. PNN La Serranía de Chiribiquete. Bosque de Tierra Firme-inundable, 0°14'54"N, 72°56'5"W, 250m, 2**♂♂**, 2000.i.28, Quevedo F., T. Exc. H. 95 (IAvH), Solano, Est. Puerto Abeja. PNN Chiribiquete. Bosque de Coluviones, 0°04'27"N, 72°27'0.5"W, 200m, **♂**, 1999.vii, Alvarez M. & Mejía G., T.Ex.E 5 (IAvH), Solano, Est. Puerto Abeja. PNN Chiribiquete. Sabanas de Gongylolepis, 0°04'27"N, 72°27'0.5"W, 200m, **♂**, 1999.vii, Alvarez M. & Mejía G., T.Ex.E 1 (IAvH), Solano, Río Mesay. PNN Chiribiquete. B. complemen, 0°15'38"N, 72°56'15"W, 300m, **♀**, 2000.ii.10, Gast F., T. Ex. T1.T6 (IAvH), Solano, Río Sararamano. PNN Chiribiquete. B. Verde militar, 0°10'48"N, 72°37'24"W, 300m, **♀**, 2000.iv.4-6, González E., T. int. (IAvH); **Guaviare:** R. Nukak M. Cr. Moyano Sta. Martha. Banqueta, 02°10'35"N, 71°10'58"W, 200m, **♀**, **♂**, ii.1996, F Escobar coll., Carroña (BDGC), Río Inirida, Caño Cocuy, Cerro Moyano, Sta. Martha. RN Nukak Maku. Tierra Firme, 02°10'35"N, 71°10'58"W, 250m, 17**♀♀**, 5**♂♂**, 1996.ii, Escobar F., Trampa excremento humano Carroña (IAvH), Río Inirida, Caño Cocuy, Cerro Moyano. RN Nukak Maku. Banqueta (Arenal), 02°10'35"N, 71°10'58"W, 250m, **♂**, 1996.ii, Escobar F., Trampa excremento humano (IAvH), Río Inirida, Caño Cocuy, Cerro Moyano. RN Nukak Maku. Bosque cerro, 02°10'35"N, 71°10'58"W, 300m, **♀**, **♂**, 1996.ii, Escobar F., T. Excremento humano (IAvH), Río Inirida, Caño Cocuy, Cerro Moyano. RN Nukak Maku. Morichal, 02°10'35"N, 71°10'58"W, 2**♂♂**, 1996.ii, Escobar F., Trampa excremento humano (IAvH).

#### Diagnosis

For all species known of *Deltohyboma*, *D*. *inesae* **sp. nov.** (*incertae sedis*) can be distinguished by it is the only species that having the pronotum without shiny points mixed with punctures, where the male posterior edge of meso- and metafemur bear a short basal expansion, most acute on mesofemur and the female have the distal margin of fifth ventrite acutely expanded medially. Also, the unique shape of the aedeagus (Fig 15J), with the paramera subequal in size to phallobase, in the other species-groups the paramera is shorter than phallobase or if are subequal in size the paramera are slender.

This species shares the following combination of character states with the *aspericolle*, *femorale*, *genieri* and *lindemannae* species-groups: anterior margin of the clypeus, between clypeal teeth, concave and expanded posteriorly into triangular shape (Fig 5D); posterior edge of metafemur with two margins (Fig 10H and 10I); ventral surface of protibia without carina or tubercles (Fig 9A–9C and 9J). It can be separated easily by conspicuous striae, which are inconspicuous in *femorale* species-group (Figs 3G and 22A). By the inter-ocular distance is under eight times the width of one eye, whereas in *aspericolle* species-group the inter-ocular distance is over nine times width of one eye.

#### Description

**Fig 33:**
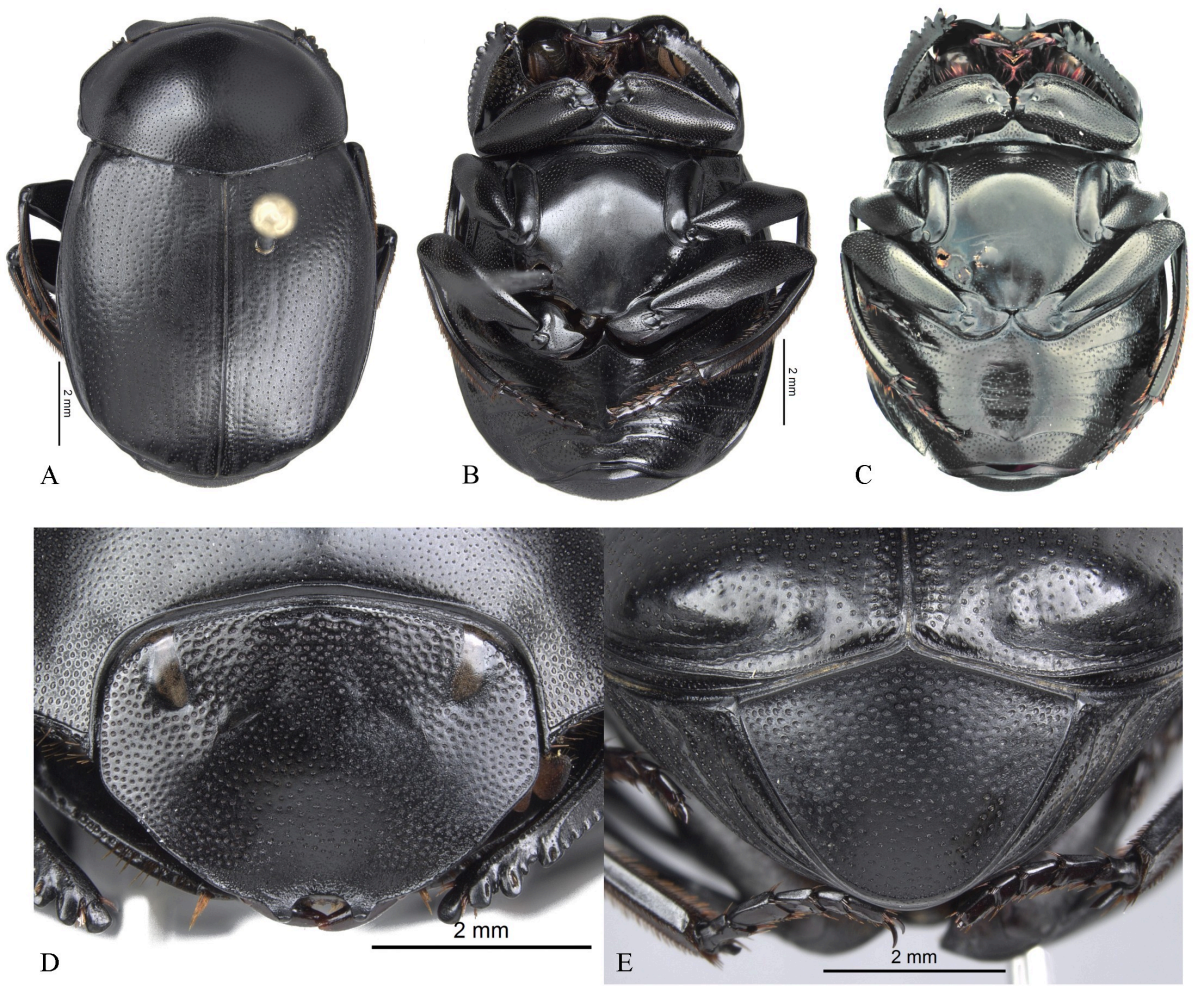
External morphology of the *Deltochilum inesae* sp. nov. Male paratype (A-B, D-E) from Colombia: Guaviare, R. Nukak; female paratype (C) from Colombia: Guaviare, R. Nukak. (A) habitus. (B-C) ventral view. (D) head. (E) caudal view.

Holotype male, length 11mm, humeral width 6.7mm. Black dorsally and ventrally (Figs 4H, 33A and 33B). **Head** (Fig 33D). Inter-ocular distance seven times width of one eye. Clypeal median emargination broadly U-shaped. Clypeal teeth separated by twice basal width of a tooth. Anterior margin of the clypeus, between clypeal teeth, concave and expanded posteriorly into triangular shape (see Fig 33C). Punctures on frons separated by less than one diameter of each puncture almost contiguous and 1.5x larger than punctures on head disc. Disc punctures separated by one diameter of each puncture. Punctures from disc towards anterior area successively larger, but without punctures between clypeal teeth. Genal punctures slightly smaller than disc punctures and separated by less than one diameter. **Pronotum** (Fig 33A). Edge between anterior and medial-lateral angle almost straight. Medial-lateral angle of pronotum rounded. Disc punctures half size to anterior-lateral ones. Basal punctures larger than anterior-lateral ones. Disc without shiny points. **Hypomera.** Internal margin of hypomera strongly enlarged towards anterior angle (see Fig 7C, arrows). **Elytra** (Fig 33A). Carina of the ninth interstria reaching middle of elytral length. Interstriae VI and VII with basal tubercles almost identical in size with approximately 3 times smaller than ninth carina. Elytral apex with tubercles poorly developed on interstriae V-VII and interstriae III with a small but well developed tubercle. Striae I-VII conspicuous and very thin, width third stria on disc approximately 1/60th of the distance between striae II and III. Punctures on first stria densest. Stria VIII conspicuous apical and laterally and reaching carina of the ninth interstria, but discontinuous near of ninth carina. Stria VIII laterally twice wide to seventh stria. Punctures on interstriae separated by one diameter, on interstria II slightly denser. Punctures of third interstria on disc occupying about 1/8th of the distance between striae II and III. **Metaventrite** (Fig 33B). Disc with few deep posterior excavation, occupying 1/3 of metaventral length and slightly elongate than width. Disc with few and small punctures, inconspicuous punctures at 8x magnification. Disc punctures almost ten times smaller than punctures on anterior-lateral area of metaventral process. Anterior-central and anterior-lateral areas of metaventral process with punctures almost contiguous. **Legs** (Fig 33B). Protibial spur broad an foliaceus. Ventral surface of the protibia without carina or tubercles. Meso- and metatrochanter not modified. Femora with punctures separated by one or less than one diameter. Mesofemur with an expansion on basal third of posterior-ventral margin, forming a large denticle. Apex of mesotibia on ventral-internal margin with a large spatulate expansion. Posterior edge of metafemur with two margins. Metafemur with a wide expansion on basal third of posterior-ventral margin, expansion wider than mesofemur expansion. Metatibial spur articulated with almost same size to first metatarsomere. Internal margin of metatibia with large tubercles occupying almost all metatibial length. **Abdomen** (Fig 33B)

Ventrite I expanded posteriorly, expansion reaching distal margin of ventrite IV. Width of expansion of the ventrite I, on ventrite III, slightly narrower than distance between clypeal teeth. Margins of expansion between ventrites II-III ventrite almost parallel, on ventrite IV forming an acute angle. Apex of expansion rounded. Distal margin of ventrite V slightly expanded medially. Medially ventrite VI as wide as V and laterally twice wider. **Pygidium** (Fig 33E). Discal punctures slightly extended transversely and separated by less than one diameter. **Genitalia**. Paramera subtriangular, dorsal edge lightly concave and ventral edge lightly convex in lateral view (Fig 15J). Paramera lightly smaller than phallobase (Fig 15J). Apex formed by the paramera truncated in dorsal view (Fig 15J’). Medial area of endophallus with one endophallite (Fig 17L) “T”-shaped. Sub-medial area of endophallus with large scales. Basal circular shape endophallite with thin ring.

#### Female (Fig 33C)

Protibial spur thinner than male. Apex of mesotibia narrower than male and on ventral-internal margin without spatulate expansion Meso- and metafemur not modified. Metasternal posterior excavation wider than length. Distal margin of ventrite V expanded medially forming an obtuse angle, expansion more acute than male. Ventrite VI slightly narrow medially.

#### Etymology

A patronym, noun in the genitive case, for first author’s grandmother María Inés Velasco.

#### Geographic distribution (Fig 31D blue circles)

This species known to be distributed, so far, in the Boreal Brazilian dominion, Imerí province, in Colombia: Caquetá, Puerto Solano, Parque Natural La Serranía de Chiribiquete and in Guaviare, Reserva Natural Nukak Maku. This species-group can be found in sympatry with *barbipes*, *femorale* and *guyanense* species-groups.

#### Remarks

The unique shape of the aedeagus (Fig 15J), in which the paramera are subequal in size to the phallobase, combined with the anterior margin of the clypeus, between clypeal teeth, concave and expanded posteriorly into a triangular shape and the mesofemur modified, is not found in any other species. Commonly, the modified mesofemur bearing an expansion or tubercles is found in species within *aequinoctiale*, *parile* and *plebejum* species-groups. However, in those species-groups the anterior margin of the clypeus, between clypeal teeth, is not expanded posteriorly into a triangular shape, the metafemur bears one margin and the medial area of endophallus has at least two endophallites. *D*. *inesae* **sp. nov.** (*incertae sedis*) bears only one medial endophallite and the metafemur bears two margins. By having two margins on the metafemur, the anterior margin of the clypeus, between clypeal teeth, expanded posteriorly into a triangular shape, the size of the eyes, the very thin striae and the body size *D*. *inesae* **sp. nov.** (*incertae sedis*) may appear to be similar to species within the *guyanense* specie-group (despite having the striae inconspicuous). However, in the latter species-group the mesofemur is unmodified and the shape of aedeagus is completely different. Due to the aforementioned, we decided not to include this species in any of the species-group and therefore leave it as *incertae sedis*.

## Discussion

Paulian [8], in the first taxonomic revision of the subgenus *Deltohyboma*, considered the number and disposition of the apical tubercles of the elytra, combined with the microsculpture of the elytra, head and pronotum, as important characters for species identification. The current study corroborates the importance of the microsculpture however, it was found that the number and disposition of the apical tubercles of the elytra varies considerably, evenly intraspecifically.

The aedeagus and the endophallus in *Deltohyboma* were found to bear critically important characters for species-group identification as well as for species identification since, in a few species-groups, such as *femorale*, *aequinoctiale*, *parile* and *plebejum*, some species can only be identified via the aedeagus. For one species-group (*irroratum*), as well as for some species-complexes within *aequinoctiale*, *parile*, *guyanense* species-groups, the examination of the endophallus is necessary for species identification. These characters are especially important when considering three geographic regions and five species-groups. The *irroratum* species-group has sympatric species in the Brazilian Mata Atlantica which are very difficult to separate otherwise; the same is true for the *barbipes* and the *guyanense* species-groups which have sympatric species in southwesternmost Amazonia and for the *guyanense* and *septemstriatum* species-groups which include species that are very difficult to separate and/or probably have cryptic species in the Guianan Lowlands, Roraima and Pantepui provinces.

The species-groups here proposed are based on characters described for first time for *Deltohyboma*, namely, the anterior margin of the clypeus (area between clypeal teeth), internal margin of hypomera, ventral face of the protibia, posterior margin of the metafemur, as well as with the first detailed comparative morphological study of the aedeagus and the endophallus. The ventral face of the protibia and the posterior margin of the metafemur were previously only described for *D*. *abdominalis* and *D*. *howdeni* by Martínez [72, 73]⁠ however, he did not use these characters in his species identifications, diagnoses or for comparative purposes. Martínez [73]⁠ also considered the aedeagus as an important character for species identification, mainly for this subgenus, and that there are some very similar species-groups.

Recently, Rossini and Vaz-de-Mello [74]⁠ discussed the relevance of the endophallic structures for taxonomy, reconstruction of evolutionary scenarios and systematics of Scarabaeinae, as well as how these structures aid in a more accurate morphological characterisation of species-groups in *Dichotomius* Hope, 1838 and which also help to propose, at least initially, hypotheses of systematic relationships. Based on the new characters and on the study of the aedeagus and the endophallus it is possible to hypothesise on potential relationships between the species-groups. The *aequinoctiale*, *parile* and *plebejum* species-groups, which are mainly distributed in the Andes, with some species in central America, appear to be closely related. The *morbillosum* and *bidentatum* species-groups share the shape of the aedeagus and they are the only two species-groups with only one humeral carina. Those five species-groups share: 1) anterior margin of the clypeus not expanded into triangular shape, 2) metafemur with one margin, where the ventral surface of metafemur forms a decline of approximately 45° on posterior-ventral edge, 3) ventral surface of protibia with tubercles and/or carina and 4) endophallus with two medial endophallites (with few exceptions in *parile* and *plebejum* species-groups). These four character states are also present in the *sextuberculatum* species-group. The *irroratum* species-group has character states 1–3 however, it does not have medial endophallites and the shape of the meso- and metatarsus is different to aforementioned species-groups. Furthermore, it is interesting that in the *irroratum* species-group there are species with the elongated metatibial insertion as in some species of the *aequinoctiale* and *plebejum* species-groups. Furthermore, the female terminalia of the *irroratum* species-group is very similar to those of the *bidentatum* species-group.

There are twelve species-groups, distributed mainly in the Amazon, that only have one medial endophallite (with few exceptions within the *barbipes* species-group). Within those species-groups, the *aspericolle*, *barbipes*, *genieri*, *gilli*, and *lindemannae* share some character states, namely, 1) anterior margin of the clypeus expanded into triangular shape, 2) metafemur with two margins, 3) ventral surface of protibia without tubercles and/or carina or with a weak carina. The *septemstriatum* and *guyanense* species-groups have most of those character states, with the exception of the anterior margin of the clypeus which is not expanded into a triangular shape. The *komareki* and *susanae* species-groups differ only by some metafemur characters states, where it bears a single margin, the ventral surface of metafemur forms a decline of approximately 45° on posterior-ventral edge, but also has one medial endophallite and the anterior margin of the clypeus is expanded into triangular shape. However, the *komareki* species-group appears to be close to *guyanense* species-group. Génier [31] considered *D*. *komareki* to be close to *D*. *crenulipes* (*guyanense* species-group) by the shape of the aedeagus and the male metafemur, to which can be added the shape of the endophallites and the base of the duct of the spermatheca (sclerotised and inverted U-shaped).

The *granulatum* and *submetallicum* species-groups are the only species-groups bearing a single margin on the posterior edge of the metafemur, where the ventral surface is continuous to the dorsal margin. Furthermore, these species-groups have one medial endophallite, the anterior margin of the clypeus is expanded into triangular shape and the ventral surface of protibia does not bear tubercles and/or carina. However, by the shape of the aedeagus and the endophallites and the distance between clypeal teeth the *granulatum* species-group appears to be close to the *barbipes* species-group. Furthermore, the *submetallicum* species-group (to which the type species of the subgenus belongs) bears a unique shape in the basal and plate shaped endophallites within *Deltohyboma*.

The *femorale* species-group, distributed in the Amazon, with the anterior margin of the clypeus expanded into triangular shape, the metafemur with two margins and the ventral surface of protibia without tubercles and/or carina, has two medial endophallites and the paramera bear an apico-lateral cleft (unique within *Deltohyboma*) and appears to be close to the others species-groups distributed in the Amazon.

In 1939, Balthasar already believed *Deltochilum* was at least twice as speciose [30], having considered the almost 80 species known at the time. In the 1950s, over a decade after Paulian’s [8] revision, a few Latin-American authors stressed the necessity of a taxonomic revision for the genus [34–38]⁠. The subgenus *Deltohyboma*, with its current 47 valid species (of which five have been newly described in the current work) and a further approximately 165 new manuscript species that have already been separated in collections (in prep.), has greatly surpassed the number of species that was initially thought to exist. This result means that *Deltochilum*, which will likely reach the 300 species mark, will therefore become the most speciose genus of the tribe Deltochilini, surpassing the currently most diverse *Canthon* with its 157 valid species [75–77]⁠, even if several new *Canthon* species are described in the near future.

Until the taxonomic revision of *Deltohyboma* is concluded, the keys for the species-groups presented here should be used for species separation in ecological and conservation studies as well as check lists, as it is infrequent that sympatric species are found within these species-groups. It should, however, be kept in mind that the *irroratum* species-group has sympatric species in the Brazilian Mata Atlantica; that the *barbipes* and the *guyanense* species-groups have sympatric species in southwesternmost Amazonia; and that the *aequinoctiale* and *plebejum* species-groups have a few sympatric species.
